# Reconstruction of Monocyte Transcriptional Regulatory Network Accompanies Monocytic Functions in Human Fibroblasts

**DOI:** 10.1371/journal.pone.0033474

**Published:** 2012-03-13

**Authors:** Takahiro Suzuki, Mika Nakano-Ikegaya, Haruka Yabukami-Okuda, Michiel de Hoon, Jessica Severin, Satomi Saga-Hatano, Jay W. Shin, Atsutaka Kubosaki, Christophe Simon, Yuki Hasegawa, Yoshihide Hayashizaki, Harukazu Suzuki

**Affiliations:** 1 Omics Science Center (OSC), RIKEN Yokohama Institute, Yokohama, Kanagawa, Japan; 2 Division of Genomic Information Resources, Supramolecular Biology, International Graduate School of Arts and Sciences, Yokohama City University, Yokohama, Kanagawa, Japan; Università degli Studi di Milano, Italy

## Abstract

Transcriptional regulatory networks (TRN) control the underlying mechanisms behind cellular functions and they are defined by a set of core transcription factors regulating cascades of peripheral genes. Here we report SPI1, CEBPA, MNDA and IRF8 as core transcription factors of monocyte TRN and demonstrate functional inductions of phagocytosis, inflammatory response and chemotaxis activities in human dermal fibroblasts. The Gene Ontology and KEGG pathway analyses also revealed notable representation of genes involved in immune response and endocytosis in fibroblasts. Moreover, monocyte TRN-inducers triggered multiple monocyte-specific genes based on the transcription factor motif response analysis and suggest that complex cellular TRNs are uniquely amenable to elicit cell-specific functions in unrelated cell types.

## Introduction

A transcriptional regulatory network (TRN), in part, defines the functional properties of a cell-type [1]. Therefore, the ability to reengineer cellular TRNs would provide vast opportunities to treat patients suffering from impaired function of physiologically important cell types such as pancreatic β-cells [2]. However, methods to identify and induce cell-specific networks are still immature and further exploration into gene regulation is much required.

Numerous attempts to induce cell reprogramming have been reported in recent years [3], [4], [5], [6], [7], [8]. Cell reprogramming, however, does not project a natural biological process, and approaches to identify a set of defined factors often deemed *hit-or-miss* or lack supporting evidence for selecting the candidate genes to induce reprogramming. Additionally, it is unknown whether every cell type can be reprogrammed. In contrast, cells reengineered to implement cell-specific function(s) would be of great value since rectifying a specific cellular function is often preferred compared to a complete replacement of impaired cells [9].

We previously described a TRN of differentiating THP-1 (human acute myeloid leukemia) cell line [1]. Utilizing two transcriptome analyses, sequencing-based CAGE [10] and microarray technologies, we characterized the dynamic regulatory activities of transcription factor binding sites and inferred the motif occupancy during a cellular differentiation. Our analysis suggested that functional characteristics of a cell-type are maintained by its specific TRN. This study also led us to believe that TRNs are driven by multiple transcription factors (TFs), and therefore various combinations of TRN elements are required to induce cellular functions.

In the present study, in order to artificially reconstruct a cellular TRN in human fibroblasts, we adopted a monocyte system mainly due to several supporting evidence of monocyte TRN [1], [11] and therapeutically applicable functions such as phagocytosis, inflammatory response and chemotaxis, which are critical for host defense and targeting cancer cells [12]. Despite the intricate connectivity of regulatory networks, TRNs are known to form a chain-of-command hierarchy [13] where a core set of TFs drive the expressions of down-stream peripheral genes and act as key modulators to induce cell-specific functions. To identify these core TFs for the monocyte network, we first integrated gene expression profiling and a text-mining strategy. Furthermore, utilizing the lentivirus system and performing a perturbation matrix analysis we then further narrowed down to *SPI1*, *CEBPA*, *IRF8* and *MNDA* as core inducers of monocyte TRN (the most up-stream elements). Ectopic expression of these four factors activated monocytic functions and revealed key monocyte-specific pathways in dermal fibroblasts. This work demonstrates that reconstruction of functional TRN can be achieved by introducing core TRN elements into unrelated cell types, such as human fibroblasts.

## Results

### Isolation of Core Transcriptional Regulatory Network Elements of Monocytes

**Figure 1:**
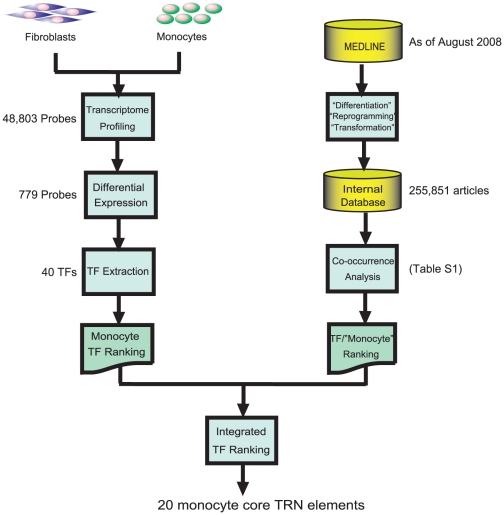
Core TRN elements isolation workflow. Key TFs were selected based on differential gene expression analysis and text mining. Left flow represents steps of the differential gene expression analysis. Gene expression profiling of primary human monocytes and that of human dermal fibroblasts were archived by using Human WG-6 v3.0 Expression beadschips (n = 3). Right flow represents the text mining. The text mining ranked the co-occurrence of “Monocyte” and “TF Name”. Finally, those two methods were integrated and isolated top 20 TFs as core monocyte TRN elements.

Based on a hierarchical-gene model [13], TRNs are thought to be driven by a defined set of core elements which represent the most upstream TFs. Core TFs control and maintain the expression of downstream peripheral genes which make up the rest of the TRN. To reconstruct the monocyte TRN, we set out to identify the core elements of monocyte TRN in two steps. First, we carried out a gene expression profiling together with literature-based text mining in order to isolate a broader set of monocyte core TRN elements. We profiled mRNA expression profiles of human CD14^+^ monocytes and human dermal fibroblasts using the Illumina microarray system. A pair-wise analysis revealed 49 highly expressed TFs in monocytes as compared to fibroblasts (2-fold or greater, *p-value*<0.05; Figure 1, left flow). Then, we implemented a literature-based text mining system to weigh the contextual relevancy of every transcription factor in monocytes (Figure 1, right flow; Table S1). We initially text mined for key biological-processes such as “differentiation”, “reprogramming” and “transformation” in all available abstracts in MEDLINE. Among the extracted abstracts, we searched for co-existence of the word “monocytes” against all functionally annotated TFs (Table S2) [14] and ranked the genes based on the highest number of co-occurrences (Table 1). Finally, we integrated both the microarray data and the text-mined gene list by adding the inverse of ranks and selected top 20 TFs as core TRN-elements of monocytes.

**Table 1:** The monocyte core TRN elements.

			Rank		Motif Activity	
PROBE_ID	SYMBOL	IS	Rfc	Rco	Fibroblast	Monocyte
ILMN_1669523	FOS	1.500	1	2	0.000986	−0.03624
ILMN_1764709	MAFB	0.511	2	90	−0.00494	−0.03165
ILMN_2086077	JUNB	0.396	16	3	0.000986	−0.03624
ILMN_1751607	FOSB	0.343	7	5	0.000986	−0.03624
ILMN_1743199	EGR2	0.338	3	218	−0.00575	−0.00267
ILMN_1666594	IRF8	0.272	4	45		
ILMN_1696463	SPI1	0.268	8	7	−0.00535	0.038682
ILMN_1727402	HCLS1	0.203	5	291		
ILMN_2392043	SPI1	0.193	20	7		
ILMN_1770085	BTG2	0.170	6	291		
ILMN_1715715	CEBPA	0.118	9	147	0.010685	0.025339
ILMN_1738992	MNDA	0.111	10	90		
ILMN_1719543	MAF	0.094	12	97		
ILMN_1753547	STAT5A	0.083	25	23	−0.01595	−0.02842
ILMN_1680624	CREG1	0.080	13	291		
ILMN_2216582	LYL1	0.075	14	291		
ILMN_1800078	LMO2	0.070	15	291	−0.00162	0.007074
ILMN_2214678	MXD1	0.065	18	105		
ILMN_1720829	ZFP36	0.058	19	174		
ILMN_1782305	NR4A2	0.047	23	291		

Rfc = differential expression fold change rank.

Rco = co-occurrence rank.

IS = Importance Score (1/Rfc+1/Rco).

### Identification of Monocyte TRN Inducers

**Figure 2:**
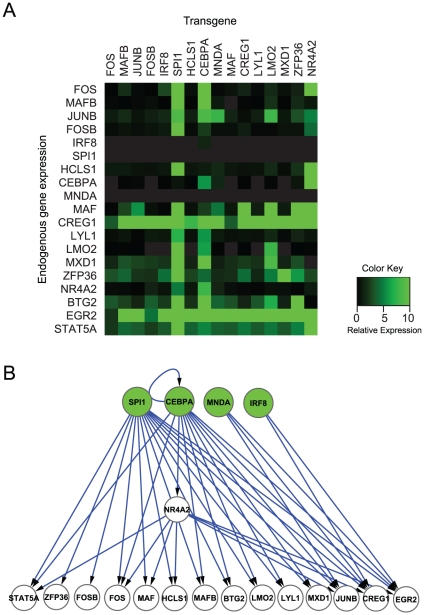
Regulatory relationship of monocyte's core TRN elements. (A) Relative expressions to monocyte are represented in colors. Higher relative expression is depicted as intensifying green color. The value of each relative expression is the average of biological replicates (n = 3). (B) Illustration of hierarchical network of monocyte core TRN elements. Each node (circle) indicates monocyte core TRN elements and green nodes represent the identified TRN inducers. When the TRN inducers or NR4A2 upregulate the gene expression to more than 5% of that of monocyte, an edge was drawn. An edge from an upper node to lower node indicates positive regulation.

In order to investigate the hierarchical structure of the broader 20-monocyte TRN-elements, we performed a lentivirus-based perturbation-matrix analysis [11]. Briefly, full-length cDNAs of the selected 16 out of 20 TRN-elements were recombined into a lentivirus vector encoding Venus (enhanced yellow fluorescent protein) (Figure S1A). We excluded *EGR2*, *BTG2*, and *STAT5A* due to a technical difficulty of lentivirus vector construction and one duplicated *SPI1* transcript variant. Each TRN-element or TF-lentivirus was then transduced onto human dermal fibroblasts for eight days and cells expressing high levels of Venus were isolated using FACS sorter. Total RNA from cells expressing unique TRN-element was used to profile endogenous transcripts of the all 19-monocyte TRN-elements (Figure 2A and Figure S2). This perturbation-matrix analysis revealed that within the investigated 16 TRN-elements, *SPI1*, *CEBPA* and *NR4A2* induced the highest numbers of other TRN-elements including *EGR2*, *BTG2*, and *STS5A*, suggesting that these three factors represent the top-line elements of the monocyte TRN structure. We also took notice that *NR4A2*, which induced the expression of eight TRN-elements, was upregulated by *CEBPA* suggesting that *NR4A2* is a down-stream gene of *CEBPA*. Moreover, the endogenous expression of *SPI1*, *IRF8* and *MNDA* could not be sufficiently expressed by any of the exogenously transduced TRN-elements, indicating that these three factors are not down-stream of the examined TRN-elements. Taken together, the perturbation-matrix analysis revealed that *SPI1*, *CEBPA*, *MNDA* and *IRF8* represent as the top-line elements of the monocyte TRN (Figure 2B) referred hereinafter as “TRN-inducers”.

### Transduction of Four TRN-Inducers Enhances Monocytic Functions

**Figure 3:**
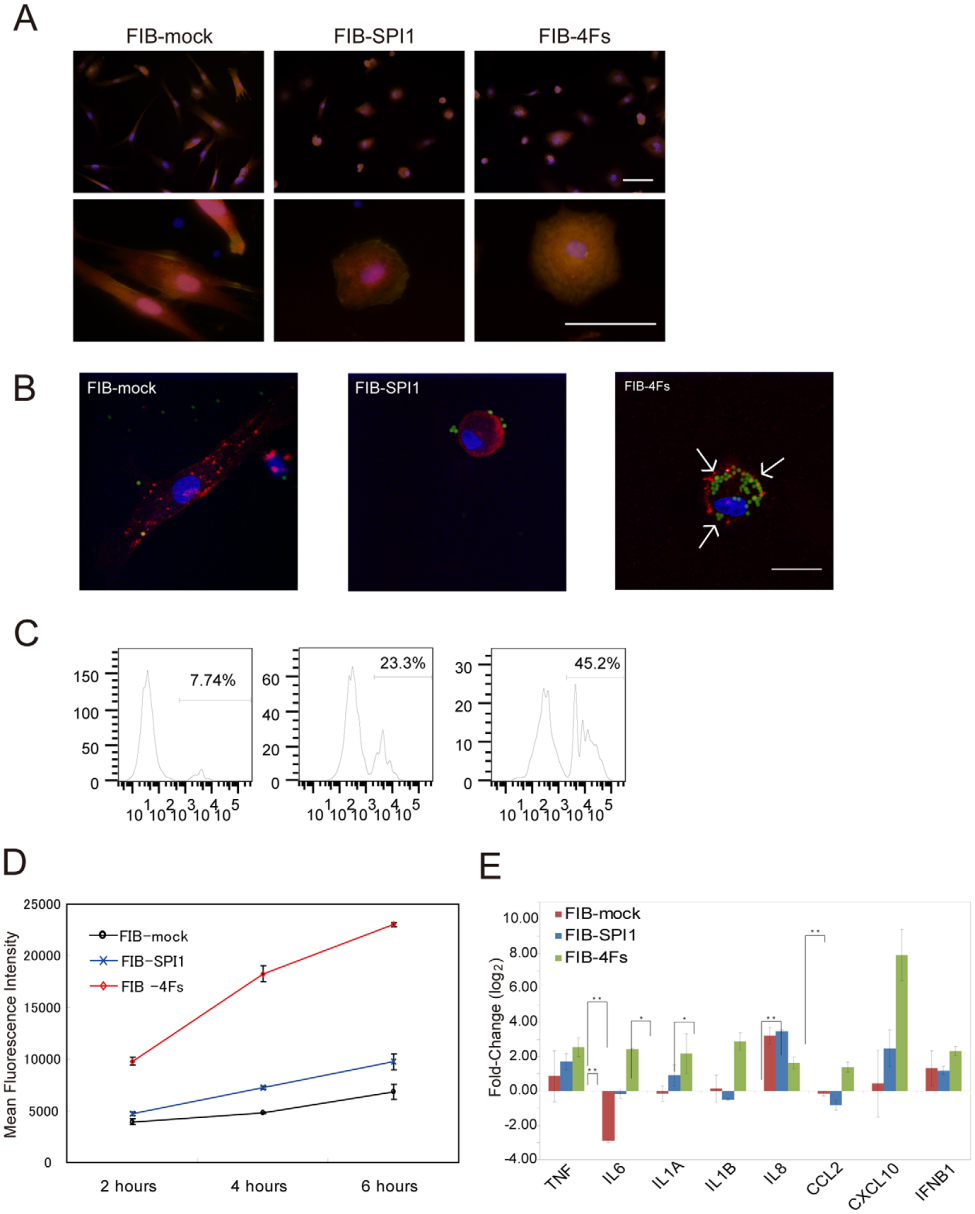
Cell feature assessments in reconstructed fibroblasts. (A) Morphological changes were visualized by microscopy. The cells were stained with Pallodin-Rhodamin (Yellow), Hoechst33342 (Blue), and Whole Cell Stains (Red). (B–D) Phagocytic latex beads were visualized (B) and the mean intensity of the ingested beads were confirmed by flow cytometry (C–D). (B) Beads-ingested cells are indicated by white arrows. Red color represents DID cell membrane staining, blue color represents nuclei, and green color represents the latex beads. (C) Beads ingested cells was quantified based on the beads fluorescence by flow cytometry in FIB-mock, FIB-SPI1 and DIB-4Fs 2 houes after beads addition.. The cells were cultured with 0.002 v/v%. Vertical and horizontal axes represent cell count and fluorescent intensity, respectively. Beads ingested cells were gated. The analysis was performed in triplicates, showing the similar results. (D) The flow cytometric analysis of the phagocytosis was summarized. Black, blue, and red lines represent FIB-mock, FIB-SPI1, and FIB-4Fs, respectively. The mean fluorescent intensity of ingested beads was measured by flow cytometry. The vertical axis and horizontal axis represent mean beads fluorescent intensity and incubation time, respectively. Error bars represent standard deviation (s.d.) (E) The expression change of *TNF*, *IL6*, *IL1A*, *IL1B*, *IL8*, *CCL2*, *CXCL10* and *IFNB1* induced by LPS treatment were measured by qRT-PCR in FIB-mock, FIB-SPI1, and FIB-4Fs. The cells were treated with LPS for 24 hours at the final concentration of 10 µg/µl. The bar represents the relative expression of LPS-treated cells as compared to untreated cells and error bar represents s.d. These experiments were repeated three times. * represents *P*-value≤0.05, ** represents *P*-value≤0.01 (t-test). The scale bar is 50 µm.

To functionally characterize the role of TRN-inducers, we constructed *SPI1*, *CEBPA*, *IRF8* and *MNDA* into DsRed, Venus, BFP (Blue fluorescence protein), and blasticidine-resistant lentivirus vectors, respectively (Figure S1A), and transduced the pooled virus into human dermal fibroblasts. Since *SPI1* is a well-known master gene of monocytes [15] and our perturbation-matrix analysis also indicated a large-scale regulation by *SPI1*, we also transduced *SPI1* alone into human fibroblasts (FIB-SPI1). In combination with fluorescent proteins and blasticidine selection, we purified the four-factor transduced fibroblasts (FIB-4Fs) using FACS (Figure S1B). Four factors or SPI1 alone remarkably induced cell morphology changes as compared with mock-lentivirus transduced fibroblasts (FIB-mock) (Figure 3A). FIB-mock maintained their spindle shape and visible F-actin fibers representative of fibroblasts-like structures, whereas, FIB-SPI1 and FIB-4Fs showed circular shapes and lacked F-actin fibers, suggesting that FIB-SPI1 and FIB-4Fs retained different cellular features than FIB-mock control.

Since phagocytosis has been implicated as one of the key functions of monocytes/macrophages [16], we cultured FIB-SPI1, FIB-4Fs and FIB-mock in the presence of 2.0 µm red fluorescent latex beads for 2, 4 and 6 hours at the final concentration of 0.002v/v%. A confocal microscopy imaging revealed strong phagocytosis activity in FIB-4Fs cells than FIB-mock, whereas FIB-SPI1 showed only a weak phagocytosis activity (Figure 3B). Further flow cytometric analysis validated the result of the confocal microscopy imaging by showing a higher signal-to-background ratio of ingested beads in FIB-4Fs cells than FIB-mock over the time course. In contrary, FIB-SPI1 showed slightly higher intensity as compared to FIB-mock (Figure 3C and D). A similar result was obtained by changing the beads concentration to 0.001v/v% (data not shown).

Next we investigated the inflammatory response to bacterial lipopolysaccharide (LPS) as another functional characteristic of monocytes/macrophages [17]. We treated FIB-mock, FIB-SPI1 and FIB-4Fs cells with or without LPS at the final concentration of 10 µg/ml for 24 hours followed by qRT-PCR to compare the expression changes of eight inflammatory responsive genes (*TNF*, *IL6*, *IL1A*, *IL1B*, *IL8*, *CCL*2, *CXCL10*, *IFNB1*; Figure 3E). The qRT-PCR analysis revealed that IL6 gene was significantly upregulated in both FIB-SPI1 and FIB-4Fs in response to LPS, however, *TNF*, *IL8* and *IFNB1* did not show any significant difference among the three samples (Figure 3E). Interestingly, in response to LPS, four genes (*IL1A*, *IL1B*, *CCL2* and *CXCL10*) were significantly upregulated only by FIB-4Fs cells and not by FIB-SPI1 and FIB-mock cells, suggesting that the inflammatory response was activated by the additional three factors and not by *SPI1* alone. Fibroblasts are found in a variety of connective tissues and have the role to synthesize the extracellular matrix and collagen [18]. However, depending on the tissue origin, fibroblasts can respond to LPS and/or possess phagocytosis activity [19], [20]. Here, we could not detect any phagocytosis or LPS response and hence we can conclude that monocytic functions were newly adapted, rather than enhanced, in these fibroblasts.

**Figure 4:**
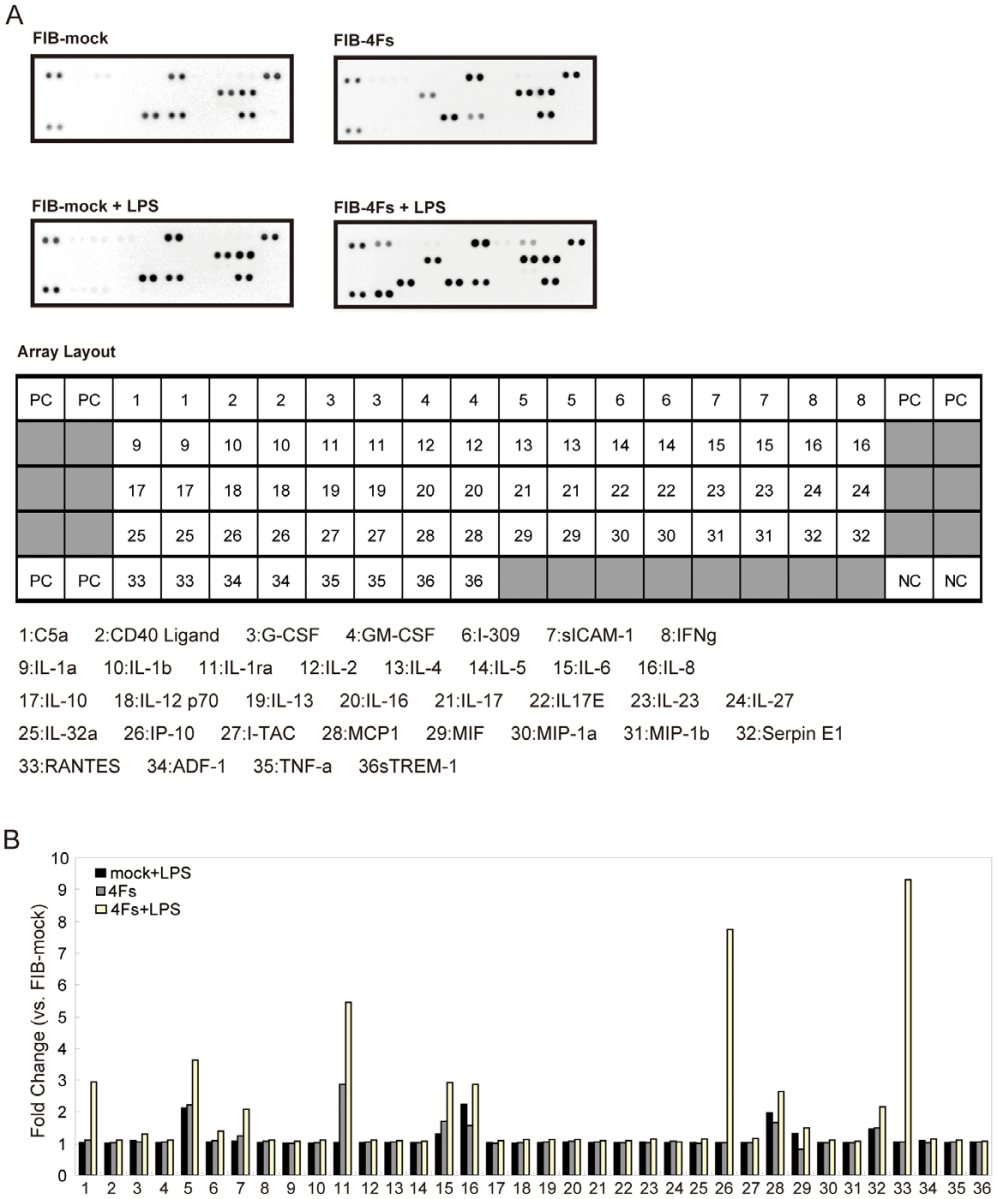
Four TRN-inducers adopted inflammatory cytokine secretion in response to LPS. FIB-mock and FIB-4Fs were treated or untreated with 10 µg/ml LPS. The cytokine levels of supernatant medium were assessed using the Proteome Profiler Human Cytokine Array, Pannel A. Array images were collected by LAS-3000 imaging system (A). The intensity of each spot was determined by Multi Gauge softwere (B).

To further confirm monocyte functions in reconstructed cells, we also carried out cytokine secretion analysis by using the proteome profiler (Figure 4). Although GROα (CXCL1) and IL-8 were slightly increased in FIB-mock (2.12- and 2.23-fold increase, respectively), C5a, IP-10 (CXCL10) and RANTES (CCL5) were strongly upregulated in FIB-4Fs in response to LPS (2,66, 7.45, and 8.85 fold increase, respectively). Although, the protein expression of GROα, sICAM-1 (CD54), IL-1ra, IL-6, IL-8, MCP-1 (CCL2) and Serpin E1 (PAI-1) were not induced in response to LPS, these cytokines were already secreted in FIB-4Fs as compared to FIB-mock, suggesting that the four factors can increase the basal level of inflammatory cytokines. These results suggest that the FIB-4Fs adopted the enhanced inflammatory response in comparisons to FIB-mock.

**Figure 5:**
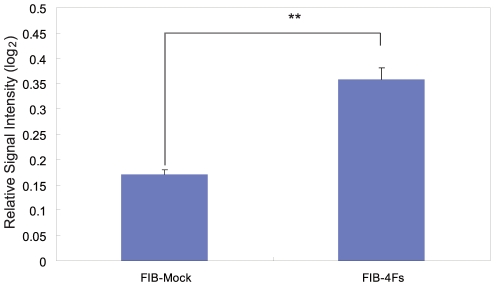
Four TRN-inducers adopted chemotaxis activity towards CCL2. Chemotaxis activity was measured by performing a transwell assay. FIB-mock and FIB-4Fs were cultured in transwells and incubated in the lower-chamber containing 5 µM of CCL2. The cells that migrated to the bottom of transwell were stained with Calcein-AM. Relative signal intensities were calculated by comparing fluorescent intensities of CCL2 treated to untreated cells (n = 3). ** represents *P-value*≤0.01 (t-test).

In order to investigate differential chemotactic response to CCL2 (also known as MCP-1) in FIB-4Fs, we seeded the cells in FluoroBlok transwell inserts and added the CCL2 protein into lower chambers at the final concentration of 5 µM and cultured for 16 hours. We stained the cells with Calcein-AM and measured fluorescent signal intensity at the bottom of the transwell inserts. The relative signal intensity was calculated by setting the samples without CCL2 as the basal chemotaxis activity (Figure 5). While we observed no chemotactic response of cells transduced with mock control, the FIB-4Fs cells displayed a significant chemotactic response (*p-value*<0.01, Figure 5). Taken together, the chemotactic response to CCL2, differential gene expression characteristics of functional monocytes, together with their morphology and marker profile, support their adaptation of monocyte TRN in dermal fibroblasts.

### Validation of the Monocyte Network Reconstruction

**Figure 6:**
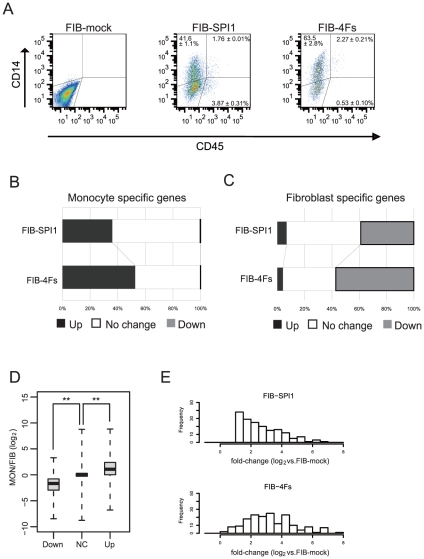
Four TRN-inducersfactors activated wider portion of monocyte TRN than SPI1 alone. (A) Flow cytometric analysis shows expression of CD14 and CD45 and no expression of CD11b in both FIB-SPI1 and FIB-4Fs. All experiments were done in duplicates. (B–C) Bar plots represent the change of up-regulated or down-regulated genes in monocyte specific genes (B) and fibroblast specific genes (C). Total length of the bar represents total number of monocyte specific genes or fibroblast specific genes (100%). The black part is 2^2^-fold grater and *P–value*<0.05 (t-test) and gray part represents 2^2^-fold less and *P-value*<0.05 (t-test), respectively, comparing to the FIB-mock. (D) All genes were categorized into either Up-regulated (Up), Down-regulated (Down) or No Change (NC) by applying the same cut-off (B–C). Medians for each dataset are indicated by black centerlines, upper quartiles are indicated by upper edges of the box, and lower quartiles are indicated by lower edges of the box. Maximum and minimum values are marked as end of lines extending from the boxes. ** represents *P-value*≤0.01 (Wilcoxson rank sum test). (E) Histogram shows distribution of the fold-change of monocyte specific and up-regulated by *SPI1* (2-fold or greater and *P-value*<0.05 (t-test)). Horizontal axis represents the log value of the fold-change as compare to FIB-mock, and vertical axis represents frequency. Upper histogram is for FIB-SPI1, and lower histogram is for FIB-4Fs.

We assessed the dynamics of monocyte marker expression by flow cytometry after transduction with FIB-4F, FIB-SPI1 and FIB-mock (Figure 6A). The analysis revealed that both FIB-4Fs and FIB-SPI1 significantly increased the percentage of CD14 (conventional monocyte marker), and the hematopoietic marker, CD45 positive cells (65.8±3.0% or 2.8±0.31% and 43.3±1.1% or 5.6±0.3%, respectively). However, the difference between FIB-SPI1 and FIB-4Fs were not statistically significant, suggesting that SPI1 also regulated monocytic genes although SPI1 alone was not sufficient to induce genes that are indispensable for the monocytic functions. In addition, we also tested CD115, CD16 and CCR2 monocyte surface markers but did not detect any positive cells (data not shown), suggesting that even FIB-4Fs is a partial conversion towards monocytes.

In order to examine the expression profiles of reconstructed monocyte TRN, we performed a global transcriptome analysis of FIB-4Fs and FIB-SPI1 using the Illumina microarrays. Based on the aforementioned dataset, we first defined the monocytes- and fibroblasts-specific genes by selecting the transcripts which expressed 2-fold or greater and *p-value*<0.03 (t-test) in monocytes (477 genes) and genes which expressed 2-fold or less and *p-value*<0.03 (t-test) in fibroblasts (488 genes), respectively. To evaluate the genes regulated in FIB-4Fs and FIB-SPI1 samples, we selected genes which were differentially expressed 2-fold or greater and *p-value*<0.05 (t-test) as compared to fibroblasts. Gene-association analysis revealed that FIB-SPI1 and FIB-4Fs induced 36.1% and 56.6% of the monocyte-specific genes, respectively (Figure 6B). The most upregulated monocyte-specific genes included well-known monocyte markers such as *CD14* (Rank:1), *IL1B* (Rank:2), *MX1* (Rank:3), *MAFB* (Rank:5) or *CD163* (Rank:12) and expression profile of well-known monocyte markers were shown in Table 2. These results suggested that TRN-inducers reconstructed the monocyte TRN to a greater degree than *SPI1* alone, although the monocyte expression profile was not completely mimicked. The analysis also showed that TRN-inducers repressed nearly 60% of fibroblast-specific genes whereas SPI1 alone repressed close to 40% (Figure 6C), suggesting an antagonistic interplay between reconstructed monocyte TRN and original fibroblast TRN. To further investigate the differential gene regulation by TRN-inducers, we categorized the genes either into up-regulated, down-regulated or unchanged based on a 2-fold cutoff (Figure 6D). Interestingly, expression of genes categorized in the up-regulated group was significantly higher in monocyte. On the other hand, expression of genes categorized in the down-regulated group were significantly lower in monocyte (*p-value*<0.001, Wilcoxon rank sum test), suggesting that differential regulation by the TRN-inducers were not random but clearly biased towards monocyte-like profile. To investigate the effect of additional factors to the *SPI1* target genes, we observed the expression distribution of SPI1 target genes. Interestingly, additional three factors (*CEBPA*, *MNDA* and *IRF8*) further up-regulated the expression of the SPI1 target genes (*p-value*<0.001, Wilcoxon signed-rank test) (Figure 6E).

**Table 2:** Expression of monocyte markers.

PROBE_ID	SYMBOL	Average signal intensity				
		Fibroblast	Monocyte	FIB-mock	FIB-SPI1	FIB-4Fs
Up regulated by 4 factors						
ILMN_1740938	APOE	62.19	4001.03	54.93	195.68	5866.35
ILMN_2396444	CD14	104.22	29343.31	106.33	7638.15	22105.52
ILMN_1678833	CCR1	62.83	2602.66	55.52	65.95	166.77
ILMN_1784863	CD36	58.72	8131.47	57.61	60.86	863.00
ILMN_2359907	CD68	550.04	10846.88	458.13	2368.70	3534.46
ILMN_1775501	IL1B	65.38	2448.35	93.12	215.54	9635.38
ILMN_2184373	IL8	216.15	21770.36	606.17	370.42	8960.96
ILMN_1728106	TNF	57.29	950.09	58.29	67.12	231.96
ILMN_1722981	TLR5	57.32	1092.46	53.31	57.93	227.43
ILMN_1654560	TLR6	63.43	1084.65	66.16	86.49	273.07
Unchanged by 4 factors						
ILMN_2413808	CD53	57.44	1629.00	59.68	75.73	72.17
ILMN_2320888	CXCR4	50.97	886.40	45.90	49.31	51.57
ILMN_2261600	FCGR1B	61.86	1044.75	63.55	69.31	72.32
ILMN_1796316	MMP9	55.14	20227.04	54.12	52.63	69.09
ILMN_2392043	SPI1	65.74	828.85	60.00	60.98	65.71
ILMN_1731048	TLR1	59.51	508.74	55.45	68.45	74.31
ILMN_1706217	TLR4	54.60	1077.68	58.77	64.74	94.18

### Functional Relevancy of Differentially Expressed Genes

We next asked whether the TRN-induced cells had acquired functional expression properties of monocytes. To achieve this we performed Gene Ontology (GO) analysis for biological process category using DAVID [21] and KEGG pathway database. The DAVID analysis revealed that both FIB-4Fs and FIB-SPI1 significantly induced genes categorized to immune system process (GO: 0002376), a major function of monocytes (Table 3). Interestingly, statistical analysis indicated that genes in this category were additionally enhanced in FIB-4Fs as compared to FIB-SPI1 (*p-value* = 6.20×10^−3^; Fisher exact test; Table 2). Furthermore, KEGG pathway analysis revealed that 13 genes induced by *SPI1* were mapped to Toll-like receptor signaling pathway involved in LPS response as compared to 21 genes by FIB-4Fs. In addition, these 13 genes induced by *SPI1* were also mapped to the endocytosis pathway in which phagocytosis process is one of the sub-categories, as compared to 20 by FIB-4Fs. Further comparison between FIB-SPI1 and FIB-4Fs revealed that lysosome pathway genes, which are involved in the post-phagocytosis process, were significantly enriched in FIB-4Fs as compared to FIB-SPI1 (*p-value*<0.05, Fisher exact test). Taken together, these results suggested that the three additional factors further enhanced the biological functions typical for monocytes in fibroblasts.

**Table 3:** Enriched Gene Ontology.

Term	P-Value (Fisher exact test)
Monocytes specific genes	
immune system process	2.00E-56
response to stimulus	5.20E-33
multi-organism process	1.40E-08
locomotion	2.20E-06
death	1.50E-04
biological adhesion	2.10E-04
biological regulation	5.50E-04
FIB-SPI1 up-regulated gene	
immune system process	1.20E-15
multi-organism process	2.30E-08
response to stimulus	3.20E-08
death	1.50E-03
locomotion	2.00E-02
developmental process	2.70E-02
localization	4.40E-02
FIB-4Fs up-regulated genes	
immune system process	2.10E-21
response to stimulus	1.70E-15
multi-organism process	1.60E-11
death	3.30E-05
locomotion	1.70E-03
localization	6.50E-03
establishment of localization	1.40E-02
biological regulation	4.10E-02
FIB-4Fs vs. FIB-SPI1	
immune system process	6.20E-03
multi-organism process	2.10E-02
response to stimulus	2.50E-02
death	3.90E-02

### Motif Activity Analysis Confirms Reconstruction of Monocyte Network

**Figure 7:**
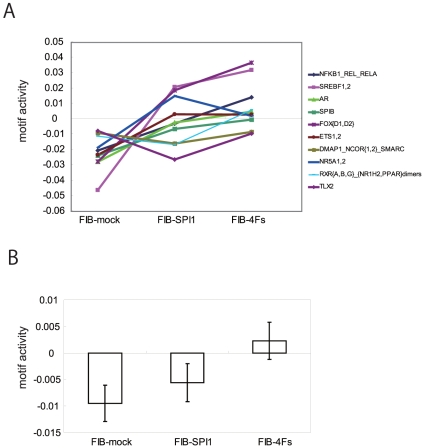
Motif activity revealed reconstructed monocyte TRN. (A) The activity of 10 monocyte-associated motifs were shown as a line chart in FIB-mock, FIB-SPI1, and FIB-4Fs. Vertical axis represents the motif activity. (B) SPI1 motif activity was shown as bar plot. Each bar represent an average of biological replicates and error bar represent s.d. (n = 3).

The average regulatory effects of each TF can be represented by computing the TF binding motif activities in a given cell type or a condition [1]. To investigate the propagation of gene expression regulated by TRN-inducers, top ten monocyte-associated motifs showed increased representations in FIB-SPI1 and more so in FIB-4Fs (Figure 7A). This result suggested that the three factors could additionally induce or enhance the monocyte TRN as compared to *SPI1* alone, possibly due to a more complete reconstruction of monocyte TRN. Interestingly, the activity of the SPI1 motif was significantly higher in FIB-4F than that in FIB-SPI1 (Figure 7B), confirming that *SPI1* works in concert with other TFs to elicit transcriptional activities at the promoter regions of target genes.

## Discussion

We have found that key biological functions of monocytes can be accompanied in human dermal fibroblasts. This was achieved by reconstructing the monocyte TRN using the four TRN-inducers and the induced cells displayed marker profile, morphology, LPS response and chemotaxis activities similar to that of monocytes. The expression profile analysis of induced cells also revealed the synergistic role of 4 TRN inducers by greatly enhancing the expression and the motif activity involved in monocyte functional pathways as compared to *SPI1* alone.

Since only a limited number of the upstream elements can induce a substantial number of the downstream genes in a hierarchical–gene model, we aimed to identify a broader set of the most upstream genes by relying not only on differential expression analysis but also on text mining for selecting TRN elements. In general, the transcript expressions of TRN-elements are not necessarily specific to a single cell-type [22], [23], and the expression levels do not always correlate strongly with the actual activity of the translated protein because of post-transcriptional modifications. Therefore, this approach is often beneficial since expression profiling alone may fail to reveal potential candidates that are highly associated to monocytes and their functions. In fact, *STAT5A*, involved in GM-CSF signaling in monocyte differentiation [24], was selected as one of the top 20 TRN-elements by text mining. We also found that *NR4A2*, another element selected by text mining, revealed a large-scale regulation of many core elements possibly a downstream gene of *CEBPA* (Figure 2A and 2B).

In the second step to identify top-line elements, we investigated the relationship among the 20 core TRN elements by perturbation-matrix analysis, which resulted in four most up-stream genes: *SPI1*, *CEBPA*, *MNDA* and *IRF8*. Interestingly, previous reports revealed that *SPI1* and *CEBPA* could convert mouse fibroblasts into macrophage-like cells [25] and mouse neural stem cells to monocyte by *SPI1* alone [26], confirming and supporting our gene selection strategy. In addition, Novershtern et. al recently analyzed the transcriptional circuit in human hematopoiesis and suggested that *SPI1*, *CEBPA* and *MNDA* may function as a monocyte/granulocytes module [27]. Especially, since *MNDA* was not previously characterized as a hematopoietic differentiation related gene, our TRN-based approach can identify the TRN-inducers even if it have not been well characterized before, suggesting that it can also be applied to other cellular functions.

*SPI1* or *PU.1* has been implicated in transdifferentiation to the myeloid lineage from fibroblasts (in conjunction with *CEBPA* [25]) and adult neural stem cells [26]. In the present study, the monocytic functions were not detected by the transduction of *SPI1* alone, a TF governing most of core TRN elements (Figure 2A and B). The reconstruction of the monocyte TRN by the four identified TRN-inducers revealed major monocyte functions, such as phagocytosis and inflammatory response to LPS. The results suggested that a set of genes associated with cellular functions is not sufficiently regulated by single TF but rather by the concerted action of multiple TFs, a mechanism often observed in cellular differentiation [1]. This mechanism is quite reasonable in terms of cellular robustness, as ectopic expression of single TF does not easily perturb important cellular features. The motif activity of *SPI1* showed an increase in their activity by four TRN-inducers whereas *SPI1* alone could not enhance it sufficiently (Figure 7B), suggesting that although this is directly explained by increase in differential expressional ratio of genes regulated by *SPI1* in FIB-4Fs (Figure 6D), a combinatorial effect may also be critical. In fact, our previous report suggested the combinatorial regulation of *SPI1* and *IRF8* were important factors during monocytic differentiation [28].

A global gene expression analysis and monocyte surface marker analysis revealed that even four factors failed to completely mimic the monocyte expression profile. This indicates that many unknown variables may contribute to the native (endogenous) monocyte TRN. The epigenetic modification may be one of the main reasons as a barrier to prevent the induction of the downstream genes of the monocyte TRN cascade. Indeed, in DNA methylation profiling, the promoter and the enhancer regions of *SPI1* were hyper-methylated in FIB, FIB-SPI1 and FIB-4Fs but not in monocytes (data not shown). While the SPI1 gene is known to be activated by an auto-regulatory feedback loop [27], [29], the endogenous expression of *SPI1* was not observed by exogenous transductions (FIB-4Fs and FIB-SPI1). This result strongly suggests that DNA methylation may prevent the SPI1 protein binding to their endogenous *SPI1* regulatory regions.

We designed and generated functional cells from human dermal fibroblasts, reconstructing the monocyte TRN by the identified four factors. In the monocytic lineage, macrophages and dendritic cells are important functional cells for host defense. Macrophages remove foreign invader such as microorganisms or apoptotic cells and dendritic cells have been reported to be applied to cancer therapy as a “dendritic cell vaccine” [30]. Hence, our TRN-based approach to generate functional cells possesses wide possibilities to consider for the cell therapies in the near future.

## Materials and Methods

### Core TRN Elements Isolation

To profile the gene expression of human CD14^+^ monocytes (Lonza Japan, Lot no. 080324B) and human neonatal fibroblasts (NB1RGB, Riken Cell Bank), Human WG-6 v3.0 Expression beadschips (Illumina) was used. The probe based signal intensity was normalized by quantile normalization with GenomeStadio softwere (Illumina). To remove un-expressed genes, genes which showed detection *P-value*<0.01 were removed. TFs were extracted based on the human TF list and ranked based on the fold-change. We registered all Illumina microarray data to Gene Expression Omnibus (http://www.ncbi.nlm.nih.gov/geo/) at NCBI (accession number GSE27304). For the text-mining, the abstracts of literatures which have words, “differentiation”, “development”, or “transformation” were collected from MEDLINE (as of August, 2008) to construct an internal database. Based on this internal database, the number of abstracts in which “monocyte” and “TF name” were co-occur was counted and ranked. To integrate the two rankings, inverse of the differential expression fold change rank (Rfc) and inverse of the co-occurrence rank (Rco) were added (importance score, IS). Based on the IS, top 20 TFs were isolated as core monocyte TRN elements.

### Cells and Cell Culture Conditions

NB1RGB is a normal human skin fibroblast established in Riken Cell Bank (RCB) from male 3 days old neonate was provided from RCB and was cultured in MEM-α (Wako) supplemented with 10% FBS and penicillin/streptomycin (100 U/ml, 100 µg/ml) (Life Technologies). Human CD14 positive monocytes (Lonza, Lot Number; 080324B) were purchased from Takara Bio bio and were cultured in RPMI1640 (Wako) supplemented with 10% FBS, 50 µM 2-mercaptoethanol (Life Technologies), 1 mM sodium pyruvate (Life Technologies), 10 mM HEPES (Life technologies), penicillin/streptomycin (100 U/ml, 100 µg/ml) (Life Technologies). a human embryonic kidney cell line, 293T cells for lentivirus production, were provided from Riken Cell Bank (RCB) and was cultured in Dulbecco's modified Eagle's medium (DMEM) (Wako) supplemented with 10% FBS and penicillin/streptomycin (100 U/ml, 100 µg/ml) (Life Technologies).

### Plasmid Construction and Preparation of Viral Vectors

The third generation Self inactivating (SIN) lentiviral vectors, CSII-EF-RfA and CSII-EF-RfA-IRES2-Venus, the packaging plasmids pCAG-HIVgp and pCMV-VSV-G-RSV-Rev were kindly provided by H. Miyoshi (RIKEN BRC). BFP fragment was amplified by using KOD-plus DNA polymerase (TOYOBO) from pREST-BFP (Life Technologies) with the primers (Forward; 5′- AATATGGCCACAACCATGGTGAGCAAGGGCGAGGAGC -3′, Reverse; 5′- TAGAGTCGCGGCCGCTTACTTGTACAGCTCGTCCATGCCGAG -3′), and Bsd fragment was amplified by the KOD-plus polymerase from pCMV-Bsd (Life Technologies) with the primers (Forward; 5′- AATATGGCCACAACCATGGCCAAGCCTTTGTCTCAAGAAG-3′, Reverse; 5′- TAGAGTCGCGGCCGCTTAGCCCTCCCACACATAACCAG-3′). These PCR products were joined with the linearized pIRES2 plasmid which was generated with the primers (Forward; 5′-GCGGCCGCGACTCTAGATCATAATC-3′, Reverse; 5′-GGTTGTGGCCATATTATCATCGTGTTTTTC-3′) to amplify the whole sequence of pIRES2-DsRed-Express2 (Clontech) except the DsRed-Express2 sequence by using the In-Fusion technology, resulting pIRES2-BFP, and pIRES2-Bsd. IRES2-BFP, IRES2-DsRed-Express2, and IRES2-Bsd fragments were amplified from these pIRES2 plasmids including the original pIRES2-DsRed-Express2 with the following primers ((IRES2-BFP) Forward; 5′-CCGCGGATCCTCTAGAGCTAGCGCTACCGGACTCAGATC-3′, Reverse; 5′-CGATGTTAACTCTAGATTACTTGTACAGCTCGTCCATGCCGAG-3′, (IRES2-DsRed-Express2) Forward; 5′-CCGCGGATCCTCTAGAGCTAGCGCTACCGGACTCAGATC-3′, Reverse; 5′-CGATGTTAACTCTAGACTACTGGAACAGGTGGTGGCG-3′, (IRES2-Bsd) Forward; 5′-CCGCGGATCCTCTAGAGCTAGCGCTACCGGACTCAGATC-3′, Reverse; 5′-CGATGTTAACTCTAGATTAGCCCTCCCACACATAACCAG-3′). These fragments were inserted into Xba I site of CSII-EF-RfA, resulting CSII-EF-RfA-IRES2-BFP (DsRed-express2, or Bsd).

Human transcription factor genes were sub-cloned from the entry clones into CSII-EF1α-RfA-IRES2 lentiviral expression vectors.

### Lentivirus Preparation

293T cells were seeded on to a poly-L-lysine courted 10 cm dish (BD) 24 hours before transfection at 4×10^6^ cells in 8 ml Opti-MEM glutaMAX (Life Technologies) supplemented with 5% FBS. One hour before transfection, the medium was replaced to fresh Opti-MEM glutaMAX medium (5% FBS, 25 µM chloroquine). The lentiviral expression vector (17 µg) and the packaging constructs, pCMV-VSV-G-RSV-Rev (10 µg) and pCAG-HIVgp (10 µg) were mixed with 1200 µl Opti-MEM (Life Technologies). Then 100 µl FuGENE HD Transfection reagent (Roche) was added to the mixture and the solution was vortex briefly, incubated for 15 min at RT. The Transfection complex was added dropwise onto the 293T cell culture. Medium was replaced to fresh Opti-MEM 24 hours after transfection. Virus containing supernatant was collected 24 hours after the medium replacement and stored at 4°C, fresh 8 ml Opti-MEM was add to the dish. The supernatant was collected 24 hours after the first medium collection and combined with the supernatant collected at first collection. Combined virus containing supernatant was filtered with 0.45 µm syringe filter (NIPPON Genetics). Filtered supernatant was ultra-centrifuged at 50000 g (19400 rpm in a Beckman SW28 rotor) for two hours at 20°C. Pelletized virus particles were resuspended in 100 µl HBSS and stored at −80°C until used.

### Lentivirus Titration

Concentrated SIN lentivirus vector was diluted 300 times with HBSS. Lentivirus RNA was extracted by using NucleoSpin® RNA Virus (MACHEREY-NAGEL) according the manufacture's instruction. Elution was done with 50 µl Elution buffer and extracted viral RNA was treated with 4 µl DNase I (Takara Bio) for 30 min at 37°C. One-step qRT-PCR was performed by using One Step SYBR PrimeScript RT-PCR Kit II (Takara Bio) with the primers (Forward; 5′-CCGTTGTCAGGCAACGTG-3′, Reverse; 5′-AGCTGACAGGTGGTGGCAAT-3′) according the manufacture's instruction. The virus titer was calculated from the Ct value based on that of the control virus whose infectivity was preliminary determined via flow cytometry.

### Flow Cytometry

Flow cytometry and cell sorting were performed with a FACSAria II SORP (BD Bioscience) and analyzed with FlowJo software. For the surface marker analysis, 1×10^6^ cells were detached with 0. 5 mM EDTA/PBS and resuspended in 1 ml washing buffer (PBS supplemented 2% FBS), incubated for 1 hour at 4°C with Alexafluor700 conjugated anti human CD14 antibody (Biolegend, Cat#; 325614, Lot#; B112226), and Alexafluor647 conjugated anti human CD45 antiybody (Biolegend, Cat#; 304018, Lot#; B116410). The cells were washed twice with the washing buffer and resuspended in 1 ml washing buffer. The combination of excitation laser and detector were Red (633 nm)-660/20 for Alexafluor647, and Red (633 nm) - 730/45 for Alexafluor700. All experiments were done in duplicates.

### Gene Transduction and Cell Selection

Human fibroblasts were seeded onto a 100 mm dish at 4×10^5^ cells with MEM-α 24 hours before infection. The medium was changed to 8 µg/ml polybrene containing medium and added lentivirus vectors at 5 or 10 MOI. The medium was replaced to fresh medium 24 hours after the infection. Cell sorting was achieved by FACS Aria II SORP. For the matrix over expression analysis, cells were cultured eight days after the gene transduction. Cells were detached and resuspended in washing buffer at 1×10^6^ cells/ml, sorted based on Venus fluorescence. For the combinatorial four factors transduction, IRF8-BFP and MNDA-Bsd were transduced into the fibroblasts and six days after the transduction, *MNDA* transduced cells were selected by adding blasticidine at the final concentration of 8 µg/ml for 10 days. After the blasticidine selection, *IRF8* transduced cells were sorted based on BFP fluorescence. The sorted cells were transduced with SPI1-DsRed and CEBPA-Venus. Eight days after the second transduction, the cells were sorted based on both DsRed and Venus fluorescence. The combination of excitation laser and detector were Violet (405 nm)-450/40 for BFP, Blue (488 nm)-530/30 for Venus, and Blue (488 nm)-610/20 for DsRed-Express2,

### Microarray Analysis

Total RNAs were extracted with FastPure RNA kit (Takara Bio) and observed their quality by using an Agilent 2100 Bioanalyzer. From 500 ng of the total RNA, biotinylated cRNA were produced with the Illumina RNA Amplification Kit (Ambion) according to the manufacturer's instructions. The concentration of the cRNA solution was determined by spectrophotometric measurement, and the size distribution of cRNA was evaluated using the Agilent 2100 Bioanalyzer. cRNA was hybridized using Human WG-6 v3.0 Expression beadschips (Illumina) according to the manufacturer's instructions. Hybridization was done in triplicate, and the signal intensity of each data set was normalized by quantile normalization. The data were analyzed with BeadStudio (Illumina), GeneSpring, and R. We registered all Illumina microarray data to Gene Expression Omnibus (http://www.ncbi.nlm.nih.gov/geo/) at NCBI (accession number GSE27304).

### Quantitative Reverse Transcription-Polymerase Chain Reaction (qRT-PCR)

Total RNA was extracted by using FastPure RNA kit (Takara Bio). Reverse transcription of total RNA was achieved with PrimeScript™ Reverse Transcriptase (Takara Bio) and random hexamers in accordance with the manufacturer's protocol. Glyceraldehyde-3-phosphate dehydrogenase (GAPDH) mRNA was used as a control (primers *5′GAAGGTGAAGGTCGGAGT-3′* and *5′GAAGATGGTGATGGGATTTC-3′* ) for data normalization. The PCR primers used for each gene in this analysis are given in Table S3. PCR amplification was performed on an ABI PRISM® 7500 Sequence Detection System (Applied Biosystems). For amplification, SYBR Premix Ex *Taq*™ II (Takara Bio) was used as instructed in the manual. The PCR conditions were an initial step of 10 seconds at 95°C, followed by 40 cycles of 3 sec at 95°C and 20 sec at 62.5°C. Changes of gene expression were determined using the 2^−ΔΔCt^ method.

### Flow Cytometric Phagocytosis Assay

Cells were incubated at 37°C at 5% CO_2_ with 0.002v/v% red fluorescent latex beads (SIGMA, diameter; 2.0 µm) for 2, 4, and 6 hours. Cells were washed five times with fresh medium and resuspended in 2% FBS-PBS to analyze the beads ingestion using the flow cytometer. The analysis was done in triplicates.

### Confocal Microscopy Imaging of Phagocytosis

Cells were incubated at 37°C, 5% CO_2_ with 0.001v/v% red fluorescent latex beads (SIGMA, diameter; 2.0 µm) for four hours in eight well Lab-Tek glass chamber slides (Nunc). The cell membrane was stained with DiD (Life technologies) and the nucleus was stained with Hoechst33343 (Life technologies), followed by confocal microscopy imaging (Leica, DMI4000B).

### Inflammatory Response Analysis

FIB-mock, FIB-SPI1, and FIB-4Fs were treated with LPS (sigma) at the final concentration of 10 µg/ml for 24 hours. The LPS treated and untreated cells were collected and their RNA was extracted. Extracted RNAs were performed qRT-PCR as described above for *TNF*, *IL6*, *IL1A*, *IL1B*, *IL8*, *CCL2*, *CXCL10* and *IFNB1*. The primer sequences were shown in Table S3. The fold-change of those genes was computed comparing with LPS untreated cells.

### Proteome profiler protein array

FIB-mock and FIB-4Fs were treated with LPS as described above on 6-well plates. The culture media were collected from the LPS-treated and -untreated wells after 16 hours. From one ml of the collected media, secreted cytokines were measured using Proteome Profiler Array Human Cytokine Array Panel A (R & D Systems) and ECL Prime Detection Reagent (GE Healthcare), following the manufacture's instruction. The chemiluminescence was detected by LAS-3000 (Fujifilm). The detected pixel density was analyzed by Multi Gauge software (Fujifilm).

### Chemotaxis activity assay

FIB-mock and FIB-4Fs were plated to trans-well inserts at 1×10^5^ cells and cultured for 24 hours with MEM-α (10% FBS). The cells were washed and cultured with Chemotaxis Assay Buffer (HBSS, 0.1% BSA). Human recombinant CCL2 (Sigma) were added to the companion plate (lower chamber) at the final concentration of 5 µM and cultured for additional 16 hours in 37°C CO_2_ incubator. The cells were stained with Calcein-AM (Life technologies) and the fluorescence intensity of the transwell inserts were measured using the ARVO plate reader system (PerkinElmer).

### Motif Activity Analysis

The motif activities were calculated as described previously [1] using position-weight matrices obtained from Swiss Regulon [31]. We searched for transcription factor binding site using the MotEvo algorithm [32] in the −300 to +100 base pair region with respect to the 5′ end of the Refseq transcript associated with each microarray probe, using a prior probability of 10^−5^.

## Supporting Information

Figure S1 **(A) Lentiviral expression vectors. (B) Strategy to collect multiple TFs transduced cells.**
(EPS)

Figure S2 **Heatmap representation of relative expressions to GAPDH.** Higher relative expression is depicted as light green color. The value of each relative expression is the average of biological replicates (n = 3).
(EPS)

Table S1 **Full result of text-mining.**
(PDF)

Table S2 **Human transcription factor list.**
(PDF)

Table S3 **Primer sequences for qRT-PCR.**
(PDF)
