# Supplementary material for: Reconstruction of Monocyte Transcriptional Regulatory Network Accompanies Monocytic Functions in Human Fibroblasts
Source: PLoS One. 2012 Mar 13;7(3):e33474. doi: 10.1371/journal.pone.0033474 (PMC3302774; doi:10.1371/journal.pone.0033474)
Supplement: Table S1 — Full result of text-mining. (PDF) [file pone.0033474.s003.pdf]

Table S1 TF/Monocyte co-occurrence

gene_name	Number of co-occurrence
TNF	1,859
IL6	1,068
CCL2	931
IL1B	720
IL4	618
IL2	595
IL10	575
CD4	559
JUN	550
CD14	536
NDUFA2	536
FOS	526
CD40	456
MAPK1	427
JUNB	395
JUND	394
FOSB	392
GRAP2	361
CSF3	351
MAPK14	351
JTV1	351
AHSA1	351
CD8A	340
CD34	329
IL3	318
SYT1	286
GORASP1	286
HSAJ2425	286
ITGAM	261
CCL5	242
CD86	224
LBR	219
LAMC2	207
TH1L	198
IMPACT	189
CD80	175
CD40LG	172
ARHGEF7	171

ITGB2	170
CD46	165
CAPG	160
C1orf132	160
TAT	154
PKC	149
TLR4	149
PTPRC	147
IFNA1	147
SPI1	143
TNFSF11	143
APC	142
KIT	139
SET	138
CD19	129
CAMP	129
CD1C	128
CD2	125
CD1B	125
IL13	125
CD1A	124
CD83	119
PSG1	117
DAND5	117
SP1	117
CSF1R	115
CD68	112
ITGAX	111
CD1D	111
FCGR3A	110
PTGS2	109
FCGR3B	109
MYC	108
ITGAL	107
STAT1	106
ISG20	105
IL2RA	104
CD1E	104
FCER2	102
TLR2	102

CD33	101
IL5	100
KRT20	98
MS4A1	98
FANCB	95
CXCL10	95
STAT3	94
HSPA9	93
CSH1	93
ERCC8	93
BCL2	91
APCS	89
MMP9	89
SRC	87
PAFAH1B1	86
YWHAE	86
CD36	86
ICAM1	85
IL9	84
NR3C2	83
CCR2	83
NCAM1	81
GPI	79
CD28	79
ADCY10	78
SCYL1	74
RPSA	74
TP63	73
CD9	72
ARHGEF2	71
PSMD7	71
EBNA1BP2	71
LANCL1	71
RABEPK	71
LOC440258	71
LOC440264	71
MGC33556	71
CCR5	71
IL8RA	71
IL15	70

PECAM1	69
MIPEP	69
CAT	68
DDX41	68
TNPO1	68
MIP	68
FAS	67
CXCR4	67
LTBR	67
TRAF2	64
CD38	63
DECR1	62
FUT4	61
EPO	60
FAT	60
ANPEP	60
TNFRSF1A	60
SLURP1	60
REL	59
IL7	58
CYP27B1	58
IL18	57
VDR	57
TNFRSF11A	56
ACP5	53
TDRD7	53
CD44	52
SOD1	52
NRSN1	52
MYB	51
MLL	51
TPPP	51
FCGR1A	51
CR2	50
HBA1	50
ANG	50
FLT3	49
CD69	49
AICDA	48
TP53	47

CEL	47
UBASH3B	47
MME	46
CD5	46
CEBPB	46
S100A9	44
CISH	44
FPR1	44
S100B	43
FCGR1B	43
FCGR1C	43
JAG1	42
PSIP1	42
NLRP1	42
SPN	41
TNFRSF1B	41
GLI2	41
STAT5A	40
TFRC	40
PARP9	40
CUX1	40
MYD88	40
RELA	40
NA	40
SOS1	39
S100A8	39
PTK2B	39
KIAA0101	39
C11orf6	38
PIGS	38
CDKN1A	38
PIK3CG	38
PIK3CA	38
PIK3CB	38
ARID1B	38
ARID3A	38
CDK5R1	38
ERG	38
CCL7	38
HAL	37

AKR1B10	37
LIF	37
STAB2	37
FSCN1	37
PIK3R3	37
PSMD12	37
IL8	37
ABL1	36
NOL3	36
CR1	36
IRF1	36
NFATC1	36
SERPINE1	36
HGF	35
TNFSF13B	35
C5AR1	35
TPO	34
TCEAL1	34
D4S234E	34
KDR	34
FCGRT	34
CSRP1	34
CRP	34
PPIAP10	34
SIGLEC7	34
THPO	33
FASTK	33
STAT5B	33
FUT1	32
BCL2L1	32
MPO	32
CD58	32
RELB	32
IL12A	32
ACE	32
CCR1	32
ARMC9	31
JAK2	31
ELK3	31
EPHB1	31

MLC1	31
DYM	31
KRIT1	31
DST	31
HMOX1	31
TIMP1	31
SLC6A2	30
ARL4C	30
ALPK1	30
STAT6	30
RARA	30
FCN2	30
GCHFR	30
UPK3B	30
PLAU	30
SPEN	29
DUOXA1	29
HSD11B1	29
FCGR2A	29
APOE	29
CD7	28
MIF	28
CTLA4	28
ATM	28
TRIM63	28
RAB40B	28
ADIPOQ	28
ATN1	27
TNFRSF8	27
IL11	27
PTPN11	27
MMRN1	27
AMH	27
LNPEP	27
HIF1A	27
CXCL9	27
HPD	27
CD55	27
VSX1	27
WISP3	27

CCL22	27
VEGFA	27
FCGR2B	27
TLR3	27
CX3CL1	27
CDKN2A	26
IFI27	26
PSMD9	26
SSSCA1	26
GNPDA1	26
NEUROD1	26
LYN	26
MBOAT4	26
TNFSF10	26
MAX	26
CFP	25
IMAGE	25
AIR	25
HLA-A	25
RHOD	25
RHO	25
TLR9	25
ADAM11	25
TRAF6	25
AGTR1	25
TBCC	24
DNTT	24
REST	24
B3GAT1	24
FASLG	24
BTK	24
CCR7	24
GALNS	24
GAST	24
BRD4	24
SERPINB6	24
PTPLA	24
SORBS1	24
CAP1	24
CTAA1	24

TNFRSF11B	24
HSPA4	24
CCR3	24
FCGR2C	24
BCR	23
FOXP3	23
NOS2A	23
CD59	23
SAA1	23
EIF4E	23
ACTB	23
IL17A	23
IL17F	23
IL1A	23
PLAUR	23
SMCP	22
MCS	22
CIITA	22
SAA	22
PAG1	22
IRF8	22
OPN1LW	22
SOCS3	22
EGF	21
PROM1	21
FBXW7	21
SYK	21
COX8A	21
RAF1	21
BCGF1	21
TEK	21
CREBBP	21
HSP90B2P	21
BTF3L1	21
ATP8A2	21
CD209	21
RAGE	21
AGER	21
PRKCDBP	20
MET	20

IL6ST	20
PDC	20
IV	20
KLK3	20
CYBB	20
EDA	20
PRAP1	20
CD163	20
MSC	19
SLC25A37	19
FLNB	19
SLTM	19
RNMT	19
LRPPRC	19
NM	19
SELL	19
IGHG3	19
SETD2	19
LCS1	19
JAK1	19
HCK	19
RHOA	19
USP9Y	19
SP3	19
SERPINB2	19
IRAK1	19
SLC33A1	19
CCL8	19
MYST3	19
REG1A	18
ABCB6	18
ITGB1	18
NOS1	18
IL23A	18
STMN1	18
IL3RA	18
SEA	18
PHB2	18
S100A10	18
ABCA1	18

TLR7	18
FGR	18
LY96	18
EHHADH	18
LBP	18
RUNX1	17
WASF1	17
LCK	17
TAG	17
TNFAIP3	17
POMC	17
LCP1	17
RNASEH2A	17
VCAM1	17
TLR1	17
NR1I2	17
TFAP2A	17
RET	17
IKBKB	17
ING1	17
MUT	17
MPL	16
CD22	16
BAX	16
TNFSF13	16
MUC1	16
CASP1	16
ERVWE1	16
AKT1	16
GCLC	16
UGCG	16
PNKD	16
TBX21	16
FUSE	16
ITGA4	16
SOCS1	16
RARS	16
SULT2A1	16
CCL4	16
FKBP4	16

TYK2	16
CD63	16
PGF	16
STAT2	16
MMP3	16
ATF7IP	16
SCT	15
SHE	15
ITGA2B	15
EGR3	15
CNTN2	15
RASA1	15
ANP32B	15
ADA	15
CXCL12	15
EXOSC1	15
CHL1	15
IGKV1-27	15
AMACR	15
WAS	15
ST13	15
EP300	15
CD52	15
CPAMD8	15
VIP	15
NOVA2	15
SMAD3	15
CCL20	15
SH2B2	15
ELA2	15
IRF9	15
MSR1	15
CDK9	15
NCF1	15
MDDC	15
ELOVL6	14
MKI67	14
RGS6	14
MYH14	14
KIDINS220	14

WDTC1	14
MVD	14
HGS	14
VWF	14
GRB2	14
ADRA1D	14
CYP2B6	14
SLCO6A1	14
S100A1	14
MYP1	14
LPL	14
SPP1	14
CD48	14
CRH	14
F9	14
CCL3	14
DPEP1	14
GABPA	14
CLEC11A	14
NFE2L2	14
MAP4K4	14
TLR5	14
PLEK	14
NSFL1C	14
IGH	13
FOXD3	13
FANCE	13
ALK	13
QRSL1	13
MTX1	13
REG3A	13
RPS4X	13
MAP6	13
SFRS5	13
HARS	13
SEC14L2	13
NUDT6	13
GUCY2D	13
CLTA	13
DNAH8	13

APP	13
HSPG2	13
PPBP	13
DPP4	13
FLT1	13
IL1RL1	13
INDO	13
CD81	13
P11	13
EXOSC6	13
XRCC1	13
RPE	13
AXPC1	13
QPCT	13
HMGB1	13
TANK	13
MAPK8	13
C1QL1	13
CAD	13
DFFB	13
B4GALNT2	13
AIRE	13
MAP3K14	13
IL16	13
IGAN	13
TAL1	12
PTPN6	12
CDKN1B	12
MBL2	12
CD27	12
FBXO8	12
FBRS	12
SHC1	12
SH2D1A	12
JAK3	12
STUB1	12
INPP5D	12
MIXL1	12
FYN	12
CCL21	12

PARP1	12
TNFRSF13B	12
CYLD	12
PML	12
IRF6	12
PIK3R1	12
CXCR3	12
CCR6	12
SELPLG	12
LMNA	12
ZHX2	12
GOPC	12
TNFRSF25	12
MS4A2	12
EPHA3	12
MAPK3	12
DYNC1H1	12
CCL19	12
CCL17	12
MAP2K1	12
ARC	12
LRP1	12
NOD2	12
CHAF1A	12
TREH	12
TRNAE1	12
RAB3GAP2	12
PIK3R4	12
CTR9	12
CMD1B	11
ABCB1	11
RRAD	11
BAD	11
CD24	11
CDKN2D	11
INVS	11
COMP	11
NR4A3	11
POU2F2	11
HTATIP	11

CYCS	11
PPP1R13B	11
PIK3R2	11
ISYNA1	11
IPS	11
HSPD1	11
MRXS5	11
HLCS	11
ST2	11
PTK2	11
IL8RB	11
PDGFB	11
MAPK9	11
FOSL1	11
IGHA1	11
CX3CR1	11
CCL1	11
IL13RA2	11
NR3C1	11
CASP3	10
FUS	10
IAPP	10
SPEF1	10
PDZK1	10
SLPI	10
PCNA	10
MTTP	10
NFE2	10
UCHL1	10
PRDX2	10
TAM	10
MITF	10
PSMA5	10
SDS	10
AHR	10
PROC	10
INS	10
COL11A2	10
STAT4	10
CDC42	10

ITGA5	10
CDK2	10
RERE	10
SPG7	10
ABL2	10
NCOA5	10
ASF1A	10
MBNL1	10
INTU	10
C10orf10	10
SART3	10
CSF1	10
NLRP3	10
EGR1	10
TAR	10
TGFB2	10
HLA-G	10
ZMYM2	10
LDLR	10
CD207	10
PDGFA	10
C9orf3	10
ALOX5	10
CTGF	10
CCL18	10
SCARB1	10
LILRB1	10
FCAR	10
TREM1	10
CCL15	10
DDIT3	9
KRT7	9
SCLY	9
ITGB3	9
NGF	9
MARCKS	9
CES2	9
PRG2	9
MBP	9
WT1	9

ENO2	9
MTG1	9
COL9A3	9
SUB1	9
CBL	9
TEC	9
TOC	9
TEC	9
PTH	9
SBDS	9
GDNF	9
BHLHB5	9
NOS3	9
DNAI1	9
NRAS	9
NT5C2	9
DC2	9
IFNG	9
TNFRSF10A	9
PAM	9
HPSE	9
SOD2	9
PTEN	9
MEFV	9
E2F1	9
THBS1	9
TNFRSF9	9
SAGE1	9
GAPDH	9
GJB6	9
PICALM	9
CADPS	9
CAPS	9
SLC3A2	9
ARCN1	9
COPD	9
EIF2AK2	9
MAFB	9
IL17D	9
TYROBP	9

CALCR	9
PLA2G2A	9
ODF1	9
CXCL5	9
TFPI	9
ALDH7A1	9
EPD	9
TCHH	9
CCDC6	9
BSG	9
SELE	9
CD53	9
TLR8	9
MNDA	9
ADAM17	9
EML1	9
ANC	8
NAAA	8
MT1E	8
AGXT	8
PRL	8
CIB1	8
CYP27A1	8
MT1JP	8
CDKN2B	8
HRAS	8
SCN8A	8
COL9A2	8
COL9A1	8
SPNS1	8
RAC1	8
RPL29	8
HHIP	8
KLRB1	8
CD47	8
BRCA1	8
PHGDH	8
MAF	8
PYCARD	8
PGD	8

KIAA1754	8
GFI1	8
RIPK1	8
CPM	8
CA8	8
UTS2R	8
CCR4	8
SLC14A2	8
RALBP1	8
CNOT6	8
C10orf97	8
ANKRD1	8
PCBD1	8
SMG1	8
ENPEP	8
TNFRSF10B	8
CSF2	8
ALOX5AP	8
CD37	8
PSMC6	8
PKLR	8
SETBP1	8
PTCH1	8
TPX2	8
MYCBP2	8
OAS3	8
TIRAP	8
SND1	8
VIPR1	8
LTA	8
PPA1	8
NPY6R	8
PPYR1	8
PAH	8
ICAM3	8
TAS2R38	8
LTB	8
AXL	8
CCL13	8
SMC2	8

NODAL	7
IDDM2	7
THY1	7
ENG	7
ST3GAL4	7
ALPP	7
AKT1S1	7
RUNX2	7
MARK1	7
ETFA	7
CXCL13	7
TOP	7
THOP1	7
NT5E	7
ETS1	7
PRNP	7
MT1B	7
NPC1	7
MAP3K8	7
STS	7
TERF1	7
SLAMF1	7
SCLC1	7
LAT	7
ORC3L	7
HLA-B	7
LAT2	7
PSMC5	7
C2	7
SLC25A10	7
IL22	7
ZBTB8OS	7
KRT31	7
DES	7
SPIB	7
IL27	7
IL7R	7
SMAD2	7
SQSTM1	7
CDKL1	7

NUP43	7
CCRK	7
CYSLTR2	7
MXD1	7
NME1	7
RBL2	7
IFNAR1	7
SARDH	7
ICOSLG	7
TICAM1	7
NR2C2	7
CXCL1	7
CHI3L1	7
CENPJ	7
CYP1A1	7
PPP2R4	7
TRIM69	7
MAP3K7	7
ABCD1	7
CXCL11	7
SERPINA1	7
EPHB6	7
MZF1	7
TLR6	7
POLD3	7
AIFM1	7
ATP7B	7
CSTB	7
NFKBIA	7
MAP2K6	7
ICAM2	7
TPT1	7
LITAF	7
GFAP	6
ESR1	6
NOTCH1	6
HECA	6
HDC	6
KITLG	6
TSPYL2	6

PIGA	6
CFC1	6
IKZF1	6
PLXNA3	6
ERBB2	6
AGRP	6
EPRS	6
HDAC9	6
ADHFE1	6
PTGDS	6
MRPL28	6
NXT1	6
NTRK1	6
SERPINB3	6
DDEF2	6
FAM126A	6
ITK	6
HCC	6
SMN1	6
RBBP8	6
CFLAR	6
RIMS1	6
PTGES3	6
PROS1	6
HRB	6
MFT	6
TNMD	6
CARD8	6
CA5A	6
SACS	6
CD244	6
C8orf4	6
SLC22A3	6
TMPRSS13	6
PSMB5	6
KRAS	6
CRYGEP1	6
CRYGC	6
FN1	6
CD99	6

AR	6
IRF2	6
ARNT	6
TSC1	6
SMS	6
CDA	6
BEST1	6
MBTPS1	6
ITGA6	6
ABCC1	6
TMEM132D	6
LAMP3	6
IL1R1	6
CSF2RA	6
MARCKSL1	6
GPR172A	6
TMPRSS11D	6
SCD	6
SMAD4	6
NR4A1	6
PADI4	6
DSP	6
GPR172B	6
CNTNAP1	6
YY1	6
TSC2	6
WDR48	6
DHX40	6
PCSK7	6
BAG3	6
CD6	6
AK3	6
MERTK	6
PRKCA	6
PSS	6
NOLC1	6
RAB3GAP1	6
PC-3	6
IVNS1ABP	6
ABCG1	6

SMAD7	6
ADM	6
AMPD1	6
CLIP1	6
ARHGEF5	6
MAP2K4	6
IL31RA	6
DLEU1	6
MARK2	6
MMP2	6
HRX	6
UMOD	6
DUSP1	6
TFDP1	6
CCL23	6
DDX5	6
CXCL2	6
NCF2	6
TNFRSF14	6
PDE4A	6
PARVA	6
CHUK	6
NAMPT	6
CCR8	6
CD93	6
CD82	6
ENO3	6
EZR	6
NOD1	6
SCYE1	6
F10	6
MLLT10	6
CEBPD	6
VAV1	5
ZAP70	5
PAX5	5
OPN1SW	5
CCL27	5
ERAL1	5
PDLIM3	5

ATRNL1	5
MYOD1	5
NAT10	5
ASRGL1	5
ATHS	5
TBC1D9	5
PF4	5
KIR3DL1	5
PPP1R13L	5
SHC3	5
RNH1	5
GEM	5
BANK1	5
ANTXR2	5
RUNX1T1	5
MT1A	5
HLA-DRB1	5
RAPH1	5
MT1L	5
PGPEP1	5
APEX1	5
HOXB4	5
LCP2	5
PDAP1	5
ACPP	5
TUSC2	5
DDEF1	5
C4BPA	5
MRPS30	5
PAPOLA	5
METAP2	5
USO1	5
CTNND1	5
PTHLH	5
TYR	5
HM13	5
C21orf63	5
MSMB	5
OSM	5
PRKG1	5

PRKAR1A	5
SULT1E1	5
LMLN	5
F8	5
MST1	5
PLAG1	5
NPEPPS	5
PI3	5
NGFR	5
PSAT1	5
BAK1	5
SLC17A5	5
SILV	5
MAGT1	5
ALPI	5
RPAIN	5
CDC2	5
SCFV	5
MGC29506	5
FGFR1	5
ADCYAP1	5
PLXNA2	5
PHYH	5
MAFK	5
ICOS	5
GTF3A	5
MOS	5
DNAH5	5
CILD2	5
MCAM	5
NANOS2	5
HPRT1	5
NF1	5
DAG1	5
CD3EAP	5
CA2	5
ERC2	5
CAST	5
HHEX	5
FZD4	5

TNFRSF18	5
LPAR3	5
TIE1	5
EFNA2	5
TBP	5
LOC441931	5
MRGPRX1	5
OXER1	5
GPR151	5
GPRC6A	5
MRGPRX3	5
MRGPRX4	5
LGR6	5
GPBAR1	5
GPR166P	5
KHDRBS1	5
CYP19A1	5
MKL1	5
FGF1	5
HAMP	5
CSK	5
ENTPD1	5
PLA2G6	5
MSX2	5
LPA	5
MAL	5
C1QBP	5
LCT	5
PPIG	5
MAG	5
JTB	5
PLA2G1B	5
LAP3	5
NFKB1	5
LAP	5
PDLA	5
YES1	5
CLIP2	5
EPHA1	5
IKBKG	5

RSS	5
PSMB9	5
MX1	5
NME2	5
WNK1	5
PITX3	5
SFTPA1	5
CEACAM8	5
AMDP1	5
AMD1	5
PMAIP1	5
SLC25A21	5
TAOK2	5
ODC1	5
WIPF2	5
C12orf57	5
MIA	5
CXCL6	5
CD97	5
LAIR1	5
PTX3	5
COX2	5
CXCL16	5
MAPK11	5
VIT	5
ZSCAN1	5
PSMB8	5
MRC1	5
CYSLTR1	5
GJA8	5
TJP1	5
SLC45A2	5
BCKDHA	5
ISG15	5
IFNA2	5
IFNA17	5
IFN1	5
PAQR7	5
IL29	5
MAPK13	5

AGTR2	5
MYST4	5
NDC80	5
SS18L1	4
CD79A	4
CDR3	4
ABCC8	4
MKKS	4
MKS1	4
ABO	4
GATA1	4
EGFR	4
KCNK3	4
BDNF	4
HTC2	4
DMD	4
RAG1	4
HES1	4
MT1IP	4
FRAP1	4
MT1G	4
MTNR1A	4
MT1M	4
MT1F	4
MT1H	4
MT1X	4
CSRP3	4
GYPA	4
CEACAM5	4
ITFG1	4
METTL8	4
TIPRL	4
MYCN	4
GP1BA	4
CXCR5	4
PTPRU	4
NXF1	4
SRY	4
POLD4	4
POLE4	4

XIAP	4
NPPA	4
IGFALS	4
CNTN3	4
SELP	4
KCNE1	4
PCS	4
CASR	4
UBL4A	4
G6PD	4
MINK1	4
S100A12	4
HOXA9	4
FGF13	4
KLRD1	4
FGF2	4
TRIM13	4
GTF2I	4
SIRPA	4
CXADR	4
NR1I3	4
CXADRP1	4
A1CF	4
CD74	4
MOCOS	4
ASPA	4
ASPM	4
NFATC2	4
CGA	4
C3	4
ATG5	4
RPS6KA3	4
ROPN1L	4
ASIP	4
ACVRL1	4
NR0B2	4
PNMA2	4
SAG	4
HMHA1	4
RNASE3	4

NCKIPSD	4
PRKAA1	4
PRKAB1	4
UBE2I	4
RNF7	4
DDX4	4
PRKAA2	4
AD5	4
S100A6	4
CEBPA	4
LYST	4
FOXO1	4
SLC12A9	4
MSH3	4
POLD1	4
FGF4	4
PMP22	4
BCS1L	4
CGB5	4
TNFRSF13C	4
CNR2	4
PITX2	4
CD274	4
HSPA5	4
GRDX	4
IGF2BP2	4
MAP3K1	4
DMN	4
TPM3	4
ACD	4
AAAS	4
TXNRD1	4
HHAT	4
MST1R	4
CTAG1B	4
UCN3	4
STIP1	4
NUP62	4
MYBL2	4
DR1	4

GPR177	4
ACCS	4
ATF2	4
DNPEP	4
DAP	4
GMPS	4
SOCS2	4
FBXL20	4
HOXA10	4
SEMA4D	4
ACSS2	4
LYPLA3	4
IRF7	4
SMARCA2	4
FLNA	4
PR	4
F11R	4
CD84	4
MED1	4
NLRP2	4
TRADD	4
NCAPG2	4
EXOSC3	4
PVRL1	4
SST	4
TIPARP	4
CIC	4
CCL16	4
SIT1	4
P2RX7	4
CEACAM6	4
PRKCB1	4
MLXIPL	4
TRAF1	4
PRDX6	4
CTNNBL1	4
NPS	4
CDK5	4
MMP14	4
LEPR	4

IL10RA	4
KLF2	4
M195	4
HSPA8	4
GRHL3	4
SAFB	4
LILRB4	4
TNFSF12	4
TXNRD3	4
CD180	4
STX1A	4
CCRL1	4
ELF3	4
MAP2K7	4
TSPAN31	4
MTRR	4
NANS	4
HOXB6	4
CEACAM4	4
MN1	4
PTGES	4
FGF9	4
IGKV2D-29	4
MGCR	4
DHS	4
ENAH	4
PLA2G10	4
EMR2	4
APOB	4
LTB4R	4
ITLN1	4
ABCC11	4
LILRB2	4
ACAA1	4
CXCL14	4
CSE	4
OSCAR	4
MAPK12	4
IER3	4
P2RY2	4

PTN	4
PROCR	4
IRAK2	4
NRTN	4
ALX4	4
TRO	4
C1S	4
F3	4
LILRA5	4
IRAK3	4
FPR2	4
CLEC4D	3
FH	3
EFS	3
EAF2	3
PDLIM7	3
RRBP1	3
EXTL3	3
ADAMTS13	3
PTS	3
SCN2A	3
FXYD5	3
ZFP36	3
KRT124P	3
SRI	3
BCL2L11	3
SH3BP4	3
AAVS1	3
BMP4	3
EVI1	3
FGFR3	3
YWHAQ	3
CEACAM3	3
GAL	3
CEACAM7	3
PSG2	3
MYOG	3
CLOCK	3
C21orf33	3
ATXN1	3

PDGFRB	3
TWIST1	3
FLT4	3
RPS19	3
PRB1	3
TNFRSF4	3
YARS	3
ACHE	3
TNFAIP1	3
BID	3
RBM45	3
NDUFB3	3
MALT1	3
GABRQ	3
POLE	3
ID2	3
HSP90AA1	3
MAPT	3
BCAR1	3
CSE1L	3
CPA1	3
IL17C	3
IL21	3
DLL1	3
CALCA	3
EEF1A2	3
BMP1	3
FES	3
MPZ	3
TBX5	3
DLD	3
TNFRSF17	3
DNAJC5	3
IL2RB	3
CD70	3
MLYCD	3
MCKD1	3
TNIP1	3
SLC19A2	3
NPM1	3

TCN1	3
GIF	3
CDK2AP2	3
SF3B14	3
RP11-336K24.9	3
RPP14	3
NBN	3
AMBP	3
ALLC	3
PRCP	3
CDKN2C	3
CRK	3
EEF1E1	3
TPPP2	3
C2orf28	3
ZNF197	3
SPIN1	3
NDUFAB1	3
TYRO3	3
ODZ1	3
NR0B1	3
SLC11A1	3
PDIA3	3
CPAT1	3
RAB35	3
LATS1	3
FADD	3
RIPK2	3
RIEG2	3
COLEC2	3
NOTCH4	3
SLC16A1	3
GAB2	3
IRF3	3
SH3YL1	3
SLC26A3	3
DDR1	3
PTBP1	3
PTBP2	3
MCAT	3

GNRH1	3
MAS1	3
EIF2C2	3
CCBP2	3
MCPH1	3
RPS6KB1	3
NOTCH3	3
FOXC1	3
AKR1B1	3
CPE	3
SNRPE	3
ABCC6	3
AVP	3
PBX1	3
USF2	3
PVR	3
PC	3
NOL1	3
SCN5A	3
GYPE	3
NSUN5	3
CCDC62	3
EDNRA	3
ESR2	3
BRD8	3
RAPGEF5	3
TYRP1	3
ARPP-21	3
TAS1R1	3
SGPL1	3
PLXNB1	3
CAMK2G	3
HSF1	3
HSPB1	3
SEPP1	3
POU2F1	3
GLI3	3
BMX	3
MDM4	3
IFI44	3

MMEL1	3
HINT1	3
VHL	3
PEBP1	3
ETS2	3
ACR	3
PTGER2	3
NR1H4	3
MMD	3
SPAG11B	3
PSMD10	3
GSTO1	3
GOSR1	3
BBS9	3
DNALI1	3
GHR	3
CEACAM1	3
POU1F1	3
GYPB	3
GRB10	3
DIAPH2	3
CLTC	3
HERPUD1	3
NCOR1	3
CCL3L1	3
INHBA	3
TRAM1	3
HSPB2	3
ZAK	3
C4A	3
CCHCR1	3
FBXL15	3
PDLIM4	3
C4B	3
CXCR6	3
SERPINA2	3
SARS2	3
SARS	3
OPN1MW	3
AIF1	3

SMOX	3
CTSA	3
MAD1L1	3
GLA	3
TSPAN4	3
CCL3L3	3
ANGPT1	3
FIGF	3
ATIC	3
KIR3DL2	3
KCNMA1	3
CLCF1	3
ETV4	3
CLC	3
ACAT1	3
LY9	3
CTF1	3
MAT1A	3
PELP1	3
CD226	3
NAT8	3
PIGU	3
CRISP2	3
GAB1	3
DADB-112B14.11	3
MYBBP1A	3
PPARG	3
TGFBI	3
NFYA	3
PTGDR	3
TNFSF18	3
DPYD	3
TEP1	3
HOXB8	3
REEP5	3
FBXW11	3
MLLT3	3
PTGS1	3
TBPL1	3
SLC25A4	3

CRX	3
PTGER1	3
TM7SF2	3
UQCRFS1	3
FEV	3
CD8B	3
RPGR	3
RGS18	3
TRAF5	3
SCGN	3
ACTN4	3
CRABP1	3
TICAM2	3
IL6R	3
BGLAP	3
RGS2	3
BMPR2	3
HLA-DRA	3
CD151	3
PEG10	3
MGP	3
TUFM	3
CTBP1	3
RNGTT	3
PFDN5	3
EMR1	3
RORC	3
TFAP4	3
IL17B	3
TNFSF15	3
IL24	3
NBPF1	3
GHRH	3
TNIK	3
TFPI2	3
CXCR7	3
RARB	3
IL10RB	3
LY86	3
PTPN12	3

ADAM8	3
NOX4	3
SIGLEC5	3
TNFSF14	3
TES	3
IL20	3
RTN3	3
MEF2D	3
TPPP3	3
CAMKV	3
SNAP91	3
HELT	3
PPARA	3
PALM	3
CYP24A1	3
CENPK	3
NCOA2	3
TNFAIP2	3
PAOX	3
SPHK1	3
HLN2	3
VSIG4	3
MAP3K7IP2	3
AOC3	3
IL20RA	3
USP18	3
ADAMTS4	3
UBR4	3
TSN	3
SNORD50A	3
SNORD50B	3
ICEBERG	3
TNIP3	3
ZC3H12A	3
NKX2-5	2
RAG2	2
SDC1	2
EPOR	2
CHM	2
NR2E3	2

KLRK1	2
ALCAM	2
PTPRN	2
CDR1	2
NAT13	2
RAB8A	2
NANOGP8	2
NANOG	2
PGC	2
PSMA7	2
RIT2	2
CD79B	2
AKR1C4	2
CDK4	2
IRF4	2
ELSPBP1	2
CD160	2
TCF7L2	2
LOH19CR1	2
PPY	2
NP	2
MLN	2
RAP1A	2
MAP2	2
PKD2L1	2
RBPJ	2
TERF2IP	2
COTL1	2
HLA-C	2
CALML3	2
PTPN22	2
PLN	2
LGALS4	2
SRF	2
BMP2	2
SMAD5OS	2
SLC2A1	2
LOC730415	2
PGP	2
CD72	2

BTG3	2
ZMYND10	2
RB1	2
RAC2	2
IGL	2
BRCA2	2
NOTCH2	2
TSPO	2
GATA2	2
DDC	2
MEF2C	2
MCL1	2
ID1	2
PIM1	2
MT2A	2
MTNR1B	2
COPE	2
FOXO3	2
KLRC1	2
IL32	2
KIAA1279	2
ITGAE	2
MEIS1	2
MSLN	2
UCP2	2
PRDM1	2
TCF7	2
ITGAV	2
DHDDS	2
TRNA	2
RAB27A	2
IL2RG	2
TRD	2
NPY	2
CMPK1	2
SMPD1	2
MTA2	2
PMCH	2
CSHL1	2
MICA	2

XXbac-BPG181B23.1	2
WNT5A	2
PIP	2
PCAF	2
MOG	2
NCR1	2
SDHD	2
ANKHD1	2
PTPRA	2
IGLL2	2
BPNT1	2
DUSP2	2
RP6-213H19.1	2
DSG1	2
HMI	2
MIA3	2
DEFA1	2
CTSL1	2
TSLP	2
STIL	2
TGFB3	2
DGCR2	2
CCNH	2
NCR3	2
NTRK2	2
TFCP2	2
HNRNPC	2
SFRS1	2
TSC22D3	2
PAEP	2
CFH	2
ID3	2
IDS	2
COL4A5	2
SLC2A10	2
PRTN3	2
ACTA1	2
PPT1	2
ANXA2	2
EIF2AK3	2

CD164	2
IGLL3	2
IBSP	2
IGLL3	2
TXN	2
BLK	2
SERPINA5	2
MROS	2
PPOX	2
MRAP	2
MAG	2
CAA	2
CTBS	2
SLC7A5	2
FOXP1	2
PCYT1B	2
HSP90B1	2
COL14A1	2
LY6E	2
LCH	2
CADM1	2
TACR1	2
MYH9	2
KLF4	2
COP1	2
MUC2	2
SLC20A1	2
ARNTL	2
RAI1	2
GEN1	2
SMCR	2
CYGB	2
TNFSF8	2
PSMD4	2
LSP1	2
LOC390387	2
SNORA73A	2
MVP	2
BCKDHB	2
UBXD5	2

PKD1	2
SIGLEC12	2
IL12B	2
SLC22A1	2
KRT122P	2
ITGA1	2
TESC	2
COL1A1	2
SCN10A	2
SLC12A3	2
BANF1	2
PTGER3	2
RAPGEF3	2
DDX53	2
LIAS	2
F2R	2
SDC2	2
ZC3H12D	2
PLAA	2
MMP12	2
HOXB3	2
CEP70	2
FHIT	2
PON1	2
FGD1	2
SGCG	2
CDIPT	2
VDAC1	2
POLE3	2
RNPC3	2
LGALS1	2
DOCK3	2
KIR2DL2	2
MTMR11	2
TIMELESS	2
RDH11	2
CYP1B1	2
ETV5	2
TRERF1	2
NFKB2	2

ADRB2	2
ARSF	2
APOBEC3G	2
8-Mar	2
EGR2	2
LYVE1	2
BCL2A1	2
EPHA4	2
FKBP1A	2
CLU	2
GAS6	2
SERPINF2	2
BEAN	2
PIAS1	2
PRSS33	2
SPARC	2
TAAR1	2
FKBP5	2
TRAF3	2
GSTM1	2
RPS6KA1	2
RAD51	2
IKZF4	2
BUD31	2
EOS	2
HOXB2	2
HOXB7	2
TRIM37	2
EDAR	2
IGKV2-23	2
FBN2	2
EPHB4	2
VIPR2	2
DDX58	2
PRKCZ	2
DTL	2
FAM20C	2
MT3	2
TNFRSF6B	2
AKAP13	2

PMPS	2
BNIP3	2
DKFZP586H2123	2
BCAP31	2
BFAR	2
NHS	2
HLA-E	2
PTPRJ	2
PDB1	2
C1R	2
MYBL1	2
GYPC	2
ENPP1	2
AFF2	2
CGN	2
NF2	2
CCL11	2
TRIP10	2
NPPC	2
IFNGR1	2
FBLIM1	2
XCL1	2
ST6GAL1	2
ALDH3A2	2
IL25	2
MRS2	2
NFIC	2
CNP	2
HPT	2
HSR	2
COL17A1	2
LIPA	2
HADHA	2
PRODH	2
SRA1	2
APBB3	2
OPRM1	2
MAP2K3	2
SFTPC	2
ADC	2

PTCRA	2
TMEM132A	2
AZU1	2
PPL	2
FLOT2	2
CCR10	2
SFRS2	2
GCG	2
NCL	2
XPO1	2
IGKV3-7	2
F12	2
ZBTB12	2
CALD1	2
RNASE2	2
PRLH	2
PDPK1	2
ABHD5	2
MT4	2
SIRT1	2
CDS1	2
MIRN223	2
FLG	2
NES	2
ENO1	2
DCD	2
CD300C	2
ELAVL1	2
DNMT3A	2
SFTPB	2
TGM2	2
TXNRD2	2
MDS1	2
SLC22A16	2
MAP3K5	2
TREM2	2
FABP4	2
PDPN	2
SERPINF1	2
PDK1	2

HSPE1	2
PHEX	2
RCP9	2
HSD11B2	2
CTCF	2
CLEC4A	2
MMVP1	2
PPR	2
EPHA2	2
YME1L1	2
CENTG1	2
DICER1	2
PIF1	2
LSS	2
ANK1	2
PTPRO	2
MLLT4	2
DOCK2	2
GPNMB	2
TBCE	2
PARC	2
LGALS3	2
NR1H2	2
DEFB4	2
MEF2A	2
TRNAD1	2
ANKH	2
MAP3K11	2
DLEU2	2
CES1	2
CCL14	2
DCTN1	2
GOLGB1	2
MXD3	2
TPD52	2
ARHGAP4	2
NRK	2
ADIPOR2	2
CBFA2T2	2
BPI	2

CES3	2
FOLR1	2
FUBP1	2
MMP10	2
KRT81	2
CIP29	2
IL26	2
HOXB1	2
HOXB5	2
HOXB9	2
ERCC2	2
BRD1	2
PLSCR1	2
DPYS	2
RAB14	2
FBP1	2
NDP	2
EBAG9	2
DOCK1	2
HMHB1	2
P2RY1	2
TRAF4	2
HMGA1	2
ITM2B	2
ECB2	2
CTSK	2
RBP3	2
TERF2	2
ERCC3	2
IGKV1-13	2
SIGLEC1	2
TAF9	2
FGL2	2
SOAT1	2
RGS1	2
HRH4	2
GTPBP1	2
MAP3K7IP1	2
PVRL2	2
IGKV1D-13	2

IFIT2	2
RGS16	2
PRX	2
C2orf3	2
COLQ	2
TFEC	2
TOX	2
PRKX	2
ILF3	2
GTF2H3	2
NRGN	2
STT3A	2
MAT2B	2
LDHA	2
LTB4R2	2
CD276	2
ST8SIA2	2
MTDH	2
STN	2
SNORD3B-1	2
SNORD3	2
TAS1R2	2
SGMS1	2
TACR2	2
KRT82	2
NR2C1	2
PCDH8	2
C18orf10	2
EMR3	2
TFPT	2
AURKA	2
GTF2H2	2
GPER	2
DBI	2
IFI30	2
GTF2H1	2
GTF2H4	2
TADA2L	2
IFNAR2	2
ACVR2B	2

MYL4	2
LOXL1	2
PGK1	2
CLEC4M	2
HLA-DRB4	2
DDOST	2
NMB	2
KRT13	2
DDX39	2
LRRC25	2
SLC7A9	2
LILRA4	2
PIGF	2
CBLB	2
PDE4D	2
SERPINB1	2
BAT1	2
CLEC5A	2
GPC3	2
APOC3	2
IL20RB	2
TNFRSF12A	2
NFKBIL1	2
MGCT	2
PTAFR	2
SIGIRR	2
TIAF1	2
TK1	2
OSBPL1A	2
NAIP	2
IL22RA2	2
ACE2	2
CARM1	2
MLLT11	2
ADAM15	2
GTF2H5	2
OTC	2
ZNF175	2
USP3	2
USP1	2

JDP2	2
IFIT3	2
MIPS	2
CD5L	2
OLR1	2
GPR68	2
SCARB2	2
HMG1L5	2
LST1	2
RP1	2
GLIPR1	2
IFIT1	2
TRIM17	2
ADAMTS5	2
TRIM67	2
HLA-F	2
CREB3	2
FPR3	2
DCI	2
OAS2	2
TNFSF12-TNFSF13	2
HNF4A	1
GAD1	1
BCL6	1
KCNJ11	1
CCND1	1
NKX2-1	1
GNAI2	1
GIP	1
C1QTNF1	1
SHH	1
POU5F1	1
ABCG2	1
ENPP3	1
FURIN	1
ETV6	1
GATA4	1
MGMT	1
LMO2	1
CTSC	1

KRT19	1
BRD2	1
GATA3	1
FRTS	1
SNAP25	1
KLRG1	1
CRS	1
MAFA	1
CCDC88A	1
ITGA2	1
TRA	1
CNTF	1
SOX9	1
PDXP	1
BCL10	1
SMUG1	1
TCL1A	1
DBF4	1
CSPG4	1
DSPP	1
MDM2	1
PCSK1	1
TNC	1
CKAP4	1
FANCA	1
TNNC1	1
UVRAG	1
KLF1	1
IGLL1	1
PLP1	1
ELF4	1
BTG2	1
H19	1
HCLS1	1
YWHAB	1
MATK	1
RFXANK	1
STATH	1
MORF4	1
ZFPM1	1

BLNK	1
CDK6	1
WNT3A	1
TAC1	1
CDAN1	1
ASCL1	1
TLX1	1
SLC12A2	1
MAP3K10	1
SLC2A4	1
RUNX3	1
MSTO1	1
SCARA3	1
MPST	1
PDGFRA	1
SLC22A2	1
PRDX5	1
FLII	1
KNTC1	1
GPR132	1
KRT17	1
TIMM8A	1
CDKL2	1
CCDC91	1
NLRP5	1
PSMC1	1
NR4A2	1
RFC1	1
CLPS	1
HBA2	1
RHOJ	1
PRS	1
GLMN	1
MAP4K1	1
LEP	1
LYPD4	1
CNR1	1
RAN	1
SP7	1
TERT	1

POLR3E	1
KIR2DL3	1
AFM	1
HESX1	1
NEU1	1
CBX8	1
MLRG	1
PRR6	1
NSD1	1
BMF	1
MRO	1
GHRL	1
LSAMP	1
ATF4	1
CFD	1
BLM	1
RCBTB1	1
ACLY	1
RBL1	1
HNRPDL	1
CYP3A4	1
SMAD1	1
SON	1
CREM	1
GARS	1
RYR1	1
PLAT	1
PDSS1	1
GOLGA6	1
EHMT1	1
CNOT8	1
CDH5	1
DPAGT1	1
LONP1	1
ZBTB16	1
GPT	1
PSEN1	1
MYH11	1
LY75	1
EPAS1	1

DEC	1
FGF8	1
NUMB	1
VAC14	1
LRPAP1	1
TAP1	1
GCA	1
TBX1	1
HMGA2	1
SLC6A3	1
SSFA2	1
MMS	1
PRDX1	1
CHKA	1
COIL	1
UCP1	1
NUCB2	1
EIF4EBP1	1
PTGER4	1
PDXK	1
CD2AP	1
NCOR2	1
PTPN2	1
TERC	1
CALR	1
SMAD5	1
NEFM	1
HLA-DPB1	1
TP73	1
SLC35A1	1
CP1	1
EIF3A	1
MRGPRF	1
ACIN1	1
KRT14	1
RBM9	1
SNAI2	1
SH2D3C	1
PSMD8	1
CHAT	1

PDCD1LG2	1
ILK	1
ARHGAP24	1
S100A4	1
HBB	1
GRIP1	1
BCL11A	1
HDAC3	1
SLAMF7	1
HLF	1
CDT1	1
DPT	1
AIFM2	1
GPR44	1
PAWR	1
EDNRB	1
SULT1A1	1
LEF1	1
AGA	1
CCL28	1
PREP	1
RAD23B	1
MAML1	1
ITGA3	1
CCR9	1
CDC2L1	1
NQO1	1
DNMT1	1
MYO1G	1
CCDC26	1
SNORD87	1
SCARNA5	1
E2F4	1
OMP	1
CCL25	1
DNAJC3	1
CASZ1	1
MLLT1	1
CNBP	1
SIL1	1

HAND1	1
MXI1	1
CFTR	1
TBXAS1	1
AGPS	1
DNMT3B	1
PSMD3	1
PRIM2	1
NTSR1	1
PLCB1	1
PLCB4	1
KLF6	1
ALKBH1	1
TTN	1
TIA1	1
EXT1	1
PIR	1
TFE3	1
LRSAM1	1
EMD	1
GGT1	1
GNAS	1
TALDO1	1
CTD	1
VHLL	1
CSF3R	1
MMP7	1
C3AR1	1
GIPC1	1
PES1	1
PRLR	1
TMEM121	1
AMELX	1
IL1RN	1
SLC6A8	1
HSPA1A	1
PLCG1	1
GRN	1
TAF8	1
B2M	1

CNC	1
IPP	1
ONECUT1	1
LYL1	1
RP4-691N24.1	1
GGTLC3	1
GGT2	1
GGTLC4P	1
GAL3ST1	1
HCCS	1
PTP4A3	1
GGTLC5P	1
PLB1	1
ITCH	1
SIM2	1
MAPK8IP1	1
MSH2	1
LPO	1
KIR2DL1	1
HSPC159	1
SAI1	1
HBD	1
WNT11	1
KRT121P	1
PKM2	1
IKZF3	1
MAFG	1
LOX	1
COPS5	1
CHPT1	1
ITGBL1	1
LAMP1	1
NOV	1
MLXIP	1
MYLIP	1
ADCYAP1R1	1
CCL4L1	1
CD177	1
GRP	1
MCM8	1

MLANA	1
PTLS	1
OVGP1	1
DDX6	1
DSC3	1
DAPK3	1
PREB	1
CHRNA4	1
PSMG1	1
ATR	1
SLC25A20	1
CDC25C	1
RPS6KA2	1
BACH2	1
PRKDC	1
FLNC	1
PLG	1
NR5A2	1
SEPN1	1
REM1	1
ARAF	1
TBK1	1
MPI	1
GTF2IRD1	1
RFX5	1
PAK1	1
CRYGD	1
MCC	1
TRIM27	1
CARD11	1
ATP2A2	1
CYP2E1	1
PKN1	1
FDPS	1
HCST	1
HOXC4	1
SCGB1A1	1
TYMS	1
PPM2C	1
CTS1	1

GCY	1
TNFSF4	1
STX2	1
KCNJ8	1
KRT85	1
LUM	1
IFIH1	1
HOXA5	1
GNA12	1
PIM2	1
HNT	1
GRIN1	1
F11	1
ZFP42	1
MIRN155	1
IL4R	1
MYST1	1
PTPN1	1
MAPK1IP1L	1
MARS	1
TCIRG1	1
ELTD1	1
FGF3	1
MMAB	1
BRIP1	1
REXO1	1
DNASE1L3	1
LYPLA2P1	1
ZC3HAV1	1
CORT	1
CAMLG	1
TAZ	1
BACH1	1
MAZ	1
TBC1D1	1
NFE2L1	1
MPG	1
ANTXR1	1
FOXO4	1
YLPM1	1

SDHB	1
MMP1	1
RPS6KA5	1
RASGRF1	1
NPHS1	1
ERBB4	1
ATP6V1E1	1
EVC2	1
SCD5	1
GSTA1	1
MTSS1	1
HEY1	1
VCP	1
USE1	1
CANX	1
MAPK7	1
OPA1	1
MTR	1
CTNNB1	1
NCOA3	1
HOXC6	1
CD109	1
DAPK1	1
CLEC2D	1
FTH1	1
HEBP1	1
CBX5	1
HOMER2	1
LHCGR	1
KIAA1524	1
DAXX	1
WASL	1
HRSP12	1
ABCC2	1
UNG	1
CD3E	1
HSH2D	1
GORASP2	1
EDARADD	1
IGFBP4	1

STX3	1
LEPROT	1
CYP1A2	1
GAA	1
CYR61	1
KIR2DS1	1
USF1	1
CREG1	1
RPL22	1
PIK3C2A	1
CSAD	1
ZNF385A	1
GZMB	1
WARS	1
FUT3	1
ZNF160	1
STAM2	1
TNP1	1
PDCD1	1
ITSN2	1
GART	1
SLC25A1	1
DDX17	1
HNRNPD	1
RNF39	1
DLX5	1
IFNB1	1
CETN2	1
RTCD1	1
ZDHHC23	1
HMBS	1
SFRS8	1
PDE3B	1
ATF6	1
C1orf9	1
DHX9	1
SOX4	1
SAT1	1
CPD	1
HFE	1

IFITM1	1
EBI3	1
SRGN	1
SLAMF6	1
SYF2	1
GTF2B	1
EIF4G2	1
DEAF1	1
ARHGDIB	1
FAM48A	1
ZFP36L1	1
RHOV	1
CBFA2T3	1
SLC6A4	1
PPP1R9B	1
GEMIN4	1
ARHGDIA	1
HDLBP	1
SDCBP2	1
BTLA	1
WWTR1	1
PROK2	1
SLBP	1
LARS	1
SGK1	1
AHI1	1
WNT2	1
IKBKAP	1
TLE1	1
HEMGN	1
AGL	1
SPA17	1
NRF1	1
LTC4S	1
EIF4G1	1
SERPIND1	1
MAPKAPK2	1
PSME1	1
PRDM16	1
CHP	1

HEXIM1	1
PPIA	1
CCRL2	1
HK2	1
NPB	1
OGG1	1
NS2	1
PLF	1
TRIT1	1
CPN1	1
CYP17A1	1
SFTPD	1
PSMA1	1
SAA3P	1
ARHGAP1	1
NUP37	1
KLHL1	1
CFDP1	1
ERN1	1
CXXC1	1
C19orf26	1
HDAC4	1
GSS	1
TST	1
TFDP2	1
DLG1	1
LMBR1	1
SKAP2	1
ANGPT2	1
YAP1	1
MARCO	1
KCNT1	1
MRAS	1
RAG1AP1	1
HOXD3	1
PSMD2	1
PAR1	1
EPB41	1
ZEB1	1
PSMA6	1

GOLGA4	1
APOBEC3C	1
YBX1	1
KCNA5	1
CYCSP38	1
LOC137886	1
PGM1	1
SMAD6	1
SMR3A	1
UNK	1
FLVCR2	1
CROT	1
GADD45G	1
CCT	1
DIH1	1
PRD	1
DBA2	1
KCNN3	1
TSG101	1
HBE1	1
NFYB	1
MYEF2	1
CYP11A1	1
SRL	1
ATP6V0D1	1
DONSON	1
RETNLB	1
CDCP1	1
THBS2	1
HLA-DMA	1
PRPH2	1
TRPV6	1
RAB11FIP1	1
FUT7	1
KIAA0020	1
POF1B	1
KAL1	1
PDCD4	1
NDUFB4	1
ST14	1

DDX3Y	1
PIAS3	1
AVPR2	1
BST1	1
HOXD4	1
CACNA1E	1
KCNH8	1
CDK5R2	1
PCK2	1
EIF2S3	1
NCOA1	1
BNIP3L	1
VISA	1
NAGLU	1
PTPRF	1
TRPM2	1
ABCC3	1
MC3R	1
SPTBN1	1
NONO	1
9-Sep	1
TSKU	1
FOXL1	1
POF1	1
ADIPOR1	1
UTS2	1
CLEC2B	1
PCID2	1
CLCN7	1
TIMP2	1
KEAP1	1
SIRT2	1
TBC1D8	1
NAG	1
ASPRV1	1
MAP4	1
SPINK1	1
HAVCR1	1
GNB2L1	1
MUTED	1

DGKB	1
CTSB	1
ANXA13	1
TPM2	1
PTPRD	1
RANBP2	1
PXMP2	1
ARHGAP26	1
TRPC1	1
HOXD10	1
CYP4V2	1
RAB5A	1
NELL1	1
SP4	1
FOSL2	1
WRN	1
AOC2	1
SPINT1	1
CDC20	1
DGKE	1
NISCH	1
HOXC5	1
MYADM	1
CKLF	1
SFRS11	1
DGUOK	1
FAM110A	1
CLEC6A	1
IGKV3D-15	1
IGKV1D-42	1
IGKV3D-7	1
TRRAP	1
CECR	1
LRE1	1
TRI	1
ORF1	1
AA1	1
RGS5	1
TPR	1
MYCL1	1

ACOT7	1
CPT1A	1
CLEC7A	1
XRCC3	1
FA2H	1
UBTF	1
ALOX15	1
HOOK2	1
INSL3	1
KLK8	1
NLRC4	1
PLXNB2	1
ZBTB20	1
TBL1XR1	1
ABCC5	1
XCR1	1
TRPC3	1
TLR10	1
SPARCL1	1
SNCA	1
HAVCR2	1
STK10	1
ADAMTS2	1
DEK	1
ETV7	1
NDUFB6	1
GAB3	1
XIST	1
REXO2	1
PRKCSH	1
TSPAN33	1
LCN2	1
GIMAP5	1
ULBP1	1
MGST1	1
HOXD11	1
HOXD8	1
RGL1	1
ACSM3	1
SRR	1

LGALS9	1
TNFRSF10C	1
RYR2	1
SLC22A18	1
ARSH	1
PICK1	1
MAFF	1
UBE2K	1
FZD2	1
LSR	1
FFAR2	1
SP2	1
PLEKHG2	1
FAAH	1
PRMT1	1
KLK2	1
KIF2A	1
SMN2	1
KLF5	1
CLN5	1
GSTZ1	1
TUBB3	1
IGKV1D-43	1
AMCN	1
CLN4	1
DGCR	1
KIR2DS5	1
XIC	1
KTWS	1
YY1AP1	1
ERCC6	1
KIN	1
APLNR	1
OSBP	1
KRT1	1
CALCOCO1	1
EFNB2	1
ABCC4	1
TP53BP1	1
SHF	1

LARP6	1
TMEM11	1
KCNH4	1
DUSP3	1
INTS2	1
CSH2	1
ASGR1	1
NT5C	1
SLC25A19	1
STAP2	1
LRG1	1
EMR4	1
SPATA2	1
ZNF148	1
PBRM1	1
MAPK10	1
EEF1B3	1
ENC1	1
HBS1L	1
MICC	1
PHIP	1
NDUFA5	1
SEMA3A	1
STC1	1
TRIM35	1
ELK1	1
WNT2B	1
SEC23IP	1
HSF2	1
MAGEC1	1
CKS1B	1
SGCD	1
FCER1A	1
SLAMF8	1
HOXD9	1
HOXD1	1
ARF1	1
RAB4A	1
LRRFIP1	1
SNED1	1

SFN	1
TGFBR2	1
RING1	1
FXYD1	1
PLK3	1
PDE1B	1
MAP3K3	1
ADRM1	1
C11orf2	1
PDE4B	1
HPR	1
DOK1	1
F2RL1	1
GAN	1
FOXF1	1
ARF1P1	1
DCR	1
ERVK2	1
ERVK3	1
IGES	1
KIR2DL4	1
PDR	1
SM1	1
ZBTB17	1
MYOC	1
RGS10	1
ABI1	1
NELL2	1
CCNT1	1
NACA	1
PPP1R12A	1
DCN	1
GZMH	1
DLG7	1
LGMN	1
DAPK2	1
SIN3A	1
KRT32	1
HN1	1
PSCD1	1

NKG7	1
EGR4	1
THBD	1
CSNK2A1	1
NRIP1	1
TYMP	1
P2RY12	1
CSPG5	1
TRAIP	1
TACR3	1
HPGD	1
CCL24	1
ADFP	1
TFG	1
CRIP2	1
GFI1B	1
PURA	1
GRM1	1
FLAD1	1
SDHC	1
HRH2	1
GRK6	1
MED15	1
C1orf107	1
LRMP	1
KLF13	1
GREM1	1
SETD1A	1
TCF19	1
RYR3	1
PPARD	1
AHRR	1
RLF	1
NMT1	1
RN7SK	1
MS4A7	1
SOCS6	1
BIRC5	1
SNORD15A	1
ANXA1	1

MC1R	1
PNRC1	1
SLC25A3	1
AFA	1
FEA	1
BPP	1
HCA1	1
C1orf102	1
AKR1C2	1
MPZL2	1
C1QTNF5	1
LTBP3	1
KLRC3	1
GPR109A	1
GPR109B	1
HTR2A	1
NDRG2	1
SLC7A8	1
TINF2	1
GMFB	1
PNMA1	1
OCA2	1
SELS	1
POLR3K	1
MYLK3	1
MYO18A	1
LILRB3	1
CCDC115	1
GBX2	1
KCNG1	1
THOC5	1
KCNJ4	1
MYLK	1
P2RY13	1
PPARGC1A	1
C5orf20	1
ADAM19	1
EPHA7	1
ATG9B	1
PABPC1	1

OPRK1	1
CDK5RAP2	1
AMN	1
RGS3	1
SORL1	1
FTMT	1
RNF139	1
RXRA	1
CASP2	1
SLC6A7	1
HSPA7	1
ADAM29	1
EPHB3	1
RGS13	1
RHOB	1
MMP11	1
SMARCB1	1
NFE2L3	1
RPL21	1
MYLK2	1
AQP1	1
ITGAD	1
DYNLRB1	1
CCNY	1
CASC5	1
CAPNS1	1
WDR76	1
SHD	1
AEBP1	1
ZNF35	1
TGM4	1
NR1H3	1
GRLF1	1
PAICS	1
PTPRG	1
TNFSF9	1
STAP1	1
HNRNPH3	1
CD300A	1
TAF1	1

FOLR2	1
OSTF1	1
ATP6V0D2	1
IGKV2D-14	1
IGKV2D-10	1
CPSF4	1
DCE	1
KIR3DS1	1
MBS1	1
MIRN15B	1
ACTD	1
DBT	1
CTSS	1
SLAMF9	1
CDC42BPA	1
INCA	1
ZNF202	1
TOLLIP	1
SAA4	1
DDB1	1
SLCO1A2	1
CSRP2	1
SPG20	1
FABP5L1	1
LTBP2	1
SNORD16	1
TSPAN3	1
ACSBG1	1
ARL6IP1	1
KRT34	1
P2RX1	1
VEZF1	1
EPR1	1
LMAN1	1
LPHN1	1
DNAJB1	1
SIGLEC11	1
NLRP12	1
NLRP7	1
NLRP11	1

CLEC4G	1
10-Sep	1
DNAJB1P	1
DTNB	1
FABP1	1
RGS19	1
NFAM1	1
CPN2	1
IMPDH2	1
LNX1	1
TSPAN5	1
GTPBP2	1
ZNF117	1
ERV3	1
PON2	1
TCEA1	1
ACSL4	1
ARD1A	1
NUDT11	1
NGFRAP1	1
GSTM2	1
P2RX4	1
ARF5	1
KHDRBS3	1
AKAP12	1
LCAP	1
TSPAN13	1
RGS14	1
5-Sep	1
CDKL5	1
NDUFA13	1
DHRS2	1
BTN3A1	1
NLRP6	1
NCF4	1
RRM1	1
ZNF382	1
MAOA	1
SLC27A2	1
FNDC3A	1

ARF6	1
FUT2	1
NUCB1	1
TINAG	1
RGS20	1
SIGLECP16	1
TRIM22	1
LILRA1	1
RCE1	1
POLS	1
C2orf13	1
ARL6IP5	1
IGBP1	1
TJP2	1
PLEKHB1	1
PAAF1	1
UPRT	1
BMP6	1
FABP5	1
XRCC4	1
SEMA4B	1
EBM	1
GTS	1
LAMA1	1
AD8	1
RA6	1
BW6	1
BW61	1
SRM	1
CHIT1	1
GCLM	1
NMT2	1
WAPAL	1
TRPC6	1
MRVI1	1
APOA1	1
ROBO4	1
TH	1
CUGBP1	1
MPEG1	1

NLRP10	1
OASL	1
RPAP3	1
TRPC4	1
SLC15A1	1
GPR18	1
SLC22A17	1
MOAP1	1
VPS39	1
IGSF6	1
GOT2	1
PSMB10	1
LCAT	1
MYH4	1
GIT1	1
HCRT	1
KDSR	1
PSG5	1
KLK15	1
GPR1	1
IGKV2-30	1
SRMS	1
FSTL1	1
ITGB5	1
RPN1	1
VPRBP	1
PDE5A	1
1-Mar	1
P4HA2	1
DIAPH1	1
DDO	1
HIST1H1D	1
TRIM10	1
DDAH2	1
TREML1	1
TREML2	1
CRIP3	1
CRISP3	1
MTPN	1
CYP11B2	1

PUF60	1
CKS1A	1
PTGES2	1
P2RY11	1
KIF1B	1
ZC3H12C	1
TRAFD1	1
IGSF2	1
DCPS	1
CALU	1
PRSS1	1
SNX9	1
ASMT	1
BLZF1	1
DDA1	1
CABLES1	1
SP100	1
SARM1	1
NR1D2	1
FARP2	1
ADAM28	1
ADRBK2	1
HIST1H1E	1
GYG2	1
BRE	1
MLLT6	1
UBAP1	1
H1F0	1
GCAT	1
RND2	1
ARL4D	1
DBF4B	1
SACM1L	1
UPP1	1
ARIH2	1
STAB1	1
LDHAL6B	1
PSMA3	1
SERPINB10	1
NLRP4	1

ZC3H12B	1
ERBB2IP	1
RABGEF1	1
NUTF2	1
EFNB1	1
TET1	1
ICT1	1
LRRC8C	1
IFIT5	1
TMPO	1
CNN2	1
KLRF1	1
ARL6	1
MARVELD1	1
FRA11B	1
LRE2	1
LRE3	1
LRE4	1
COX1	1
PRR1	1
TRNAP1	1
TYS	1
RCD2	1
SBS	1
UGT1A9	1
UGT1A1	1
KIR2DL5A	1
MIRN146A	1
BW62	1
KIR2DL5B	1
WARS2	1
DDAH1	1
RSU1	1
HTR7	1
MMP27	1
BACE1	1
SNORA54	1
C11orf40	1
CKAP5	1
MACROD1	1

PTPRCAP	1
CACNA2D4	1
YARS2	1
ABCD2	1
PAN2	1
HEXA	1
ACCN1	1
CDH19	1
ZNF675	1
TBXA2R	1
PSG11	1
PSG9	1
GPR4	1
MYO1F	1
RDH14	1
NAGA	1
DUSP7	1
GBE1	1
CDH12	1
SEMA5A	1
SRPK2	1
SETX	1
OAS1	1
LRRC8A	1
NARG1	1
NOTCH2NL	1
NOMO1	1
ACCN3	1
MAP3K13	1
CFHR3	1
GBL	1
ACCN4	1
SORBS3	1
GUK1	1
DUSP22	1
NOL5A	1
ZNF267	1
CAPRIN1	1
WDR19	1
PDE9A	1

RBM3	1
ACCN2	1
PAQR8	1
HNRNPA1	1
PRKCH	1
ZNF324	1
SLC25A26	1
SLC7A6	1
ZFAND6	1
LRRC8E	1
LRRC8B	1
IGKV1D-8	1
LRRC8D	1
LPPR4	1
CNPY4	1
BCRL3	1
CORD1	1
FSP	1
HCL3	1
ZNF126	1
TRR	1
PRG1	1
UGT1A10	1
UGT1A8	1
UGT1A7	1
UGT1A6	1
UGT1A5	1
UGT1A4	1
RLFP	1
C22orf17	1
