# Supplementary material for: Reconstruction of Monocyte Transcriptional Regulatory Network Accompanies Monocytic Functions in Human Fibroblasts
Source: PLoS One. 2012 Mar 13;7(3):e33474. doi: 10.1371/journal.pone.0033474 (PMC3302774; doi:10.1371/journal.pone.0033474)
Supplement: Table S2 — Human transcription factor list. (PDF) [file pone.0033474.s004.pdf]

Table S2 Human transcription factor list

EntrezGene	Symbol	Symbol_TR	Gene Name_TR
26574	AATF	AATF	APOPTOSIS ANTAGONIZING TRANSCRIPTION FACTOR
136647	C7orf11	ABHS	CHROMOSOME 7 OPEN READING FRAME 11
25	ABL1	ABL1	V-ABL ABELSON MURINE LEUKEMIA VIRAL ONCOGENE HOMOLOG 1
29777	ABT1	ABT1	ACTIVATOR OF BASAL TRANSCRIPTION 1
80325	ABTB1	ABTB1	ANKYRIN REPEAT AND BTB (POZ) DOMAIN CONTAINING 1
23394	ADNP	ADNP	ACTIVITY-DEPENDENT NEUROPROTECTOR
22850	KIAA0863	ADNP2	ADNP homeobox 2
165	AEBP1	AEBP1	AE BINDING PROTEIN 1
121536	AEBP2	AEBP2	AE BINDING PROTEIN 2
166	AES	AES	AMINO-TERMINAL ENHANCER OF SPLIT
4299	AFF1	AFF1	AF4/FMR2 FAMILY, MEMBER 1
3899	AFF3	AFF3	AF4/FMR2 FAMILY, MEMBER 3
27125	AFF4	AFF4	AF4/FMR2 FAMILY, MEMBER 4
25909	AHCTF1	AHCTF1	AT HOOK CONTAINING TRANSCRIPTION FACTOR 1
196	AHR	AHR	ARYL HYDROCARBON RECEPTOR
9049	AIP	AIP	ARYL HYDROCARBON RECEPTOR INTERACTING PROTEIN
326	AIRE	AIRE	AUTOIMMUNE REGULATOR (AUTOIMMUNE POLYENDOCRINOPATHY CANDIDIASIS ECTODERMAL DYSTROPHY)
11036	ALF	ALF	TFIIA-ALPHA/BETA-LIKE FACTOR
79800	ALS2CR8	ALS2CR8	AMYOTROPHIC LATERAL SCLEROSIS 2 (JUVENILE) CHROMOSOME REGION, CANDIDATE 8
257	ALX3	ALX3	ARISTALESS-LIKE HOMEOBOX 3
60529	ALX4	ALX4	ARISTALESS-LIKE HOMEOBOX 4
57763	ANKRA2	ANKRA2	ANKYRIN REPEAT, FAMILY A (RFXANK-LIKE), 2
27063	ANKRD1	ANKRD1	ANKYRIN REPEAT DOMAIN 1 (CARDIAC MUSCLE)
55608	ANKRD10	ANKRD10	ANKYRIN REPEAT DOMAIN 10
26287	ANKRD2	ANKRD2	ANKYRIN REPEAT DOMAIN 2 (STRETCH RESPONSIVE MUSCLE)
118932	ANKRD22	ANKRD22	ANKYRIN REPEAT DOMAIN 22
25959	ANKRD25	ANKRD25	ANKYRIN REPEAT DOMAIN 25
91074	ANKRD30A	ANKRD30A	ANKYRIN REPEAT DOMAIN 30A
84250	ANKRD32	ANKRD32	ANKYRIN REPEAT DOMAIN 32
341405	ANKRD33	ANKRD33	ANKYRIN REPEAT DOMAIN 33
338699	ANKRD42	ANKRD42	ANKYRIN REPEAT DOMAIN 42
339416	ANKRD45	ANKRD45	ANKYRIN REPEAT DOMAIN 45
54851	ANKRD49	ANKRD49	ANKYRIN REPEAT DOMAIN 49
63926	ANKRD5	ANKRD5	ANKYRIN REPEAT DOMAIN 5
129138	LOC129138	ANKRD54	ankyrin repeat domain 54
79722	FLJ11795	ANKRD55	ankyrin repeat domain 55
65124	C2orf26	ANKRD57	ankyrin repeat domain 57
22881	ANKRD6	ANKRD6	ANKYRIN REPEAT DOMAIN 6
56311	ANKRD7	ANKRD7	ANKYRIN REPEAT DOMAIN 7
23294	ANKS1A	ANKS1A	ANKYRIN REPEAT AND STERILE ALPHA MOTIF DOMAIN CONTAINING 1A
124401	ANKS3	ANKS3	ANKYRIN REPEAT AND STERILE ALPHA MOTIF DOMAIN CONTAINING 3
55139	ANKZF1	ANKZF1	ANKYRIN REPEAT AND ZINC FINGER DOMAIN CONTAINING 1
322	APBB1	APBB1	AMYLOID BETA (A4) PRECURSOR PROTEIN-BINDING, FAMILY B, MEMBER 1 (FE65)
323	APBB2	APBB2	AMYLOID BETA (A4) PRECURSOR PROTEIN-BINDING, FAMILY B, MEMBER 2 (FE65-LIKE)
324	APC	APC	ADENOMATOSIS POLYPOSIS COLI
328	APEX1	APEX1	APEX NUCLEASE (MULTIFUNCTIONAL DNA REPAIR ENZYME) 1
367	AR	AR	ANDROGEN RECEPTOR (DIHYDROTESTOSTERONE RECEPTOR; TESTICULAR FEMINIZATION; SPINAL AND BULBAR MUSCULAR ATROPHY; KENNEDY DISEASE)
8289	ARID1A	ARID1A	AT RICH INTERACTIVE DOMAIN 1A (SWI- LIKE)
57492	ARID1B	ARID1B	DAN15 PROTEIN
1820	ARID3A	ARID3A	AT RICH INTERACTIVE DOMAIN 3A (BRIGHT- LIKE)
10620	ARID3B	ARID3B	AT RICH INTERACTIVE DOMAIN 3B (BRIGHT- LIKE)
5926	ARID4A	ARID4A	AT RICH INTERACTIVE DOMAIN 4A (RBP1-LIKE)
51742	ARID4B	ARID4B	AT RICH INTERACTIVE DOMAIN 4B (RBP1- LIKE)
84159	ARID5B	ARID5B	AT RICH INTERACTIVE DOMAIN 5B (MRF1-LIKE)
405	ARNT	ARNT	ARYL HYDROCARBON RECEPTOR NUCLEAR TRANSLOCATOR
9915	ARNT2	ARNT2	ARYL-HYDROCARBON RECEPTOR NUCLEAR TRANSLOCATOR 2
406	ARNTL	ARNTL	ARYL HYDROCARBON RECEPTOR NUCLEAR TRANSLOCATOR-LIKE
56938	ARNTL2	ARNTL2	ARYL HYDROCARBON RECEPTOR NUCLEAR TRANSLOCATOR-LIKE 2
170302	ARX	ARX	ARISTALESS RELATED HOMEOBOX
51665	ASB1	ASB1	ANKYRIN REPEAT AND SOCS BOX-CONTAINING 1
136371	ASB10	ASB10	ANKYRIN REPEAT AND SOCS BOX-CONTAINING 10
140456	ASB11	ASB11	ANKYRIN REPEAT AND SOCS BOX-CONTAINING 11
142689	ASB12	ASB12	ANKYRIN REPEAT AND SOCS BOX-CONTAINING 12
142685	ASB15	ASB15	ANKYRIN REPEAT AND SOCS BOX-CONTAINING 15
51130	ASB3	ASB3	ANKYRIN REPEAT AND SOCS BOX-CONTAINING 3
51666	ASB4	ASB4	ANKYRIN REPEAT AND SOCS BOX-CONTAINING 4
140459	ASB6	ASB6	LIKELY ORTHOLOG OF MOUSE ANKYRIN REPEAT AND SOCS BOX-CONTAINING PROTEIN 6
140461	ASB8	ASB8	ANKYRIN REPEAT AND SOCS BOX-CONTAINING 8
140462	ASB9	ASB9	DKFZP564L0862 PROTEIN
51008	ASCC1	ASCC1	ACTIVATING SIGNAL COINTEGRATOR 1 COMPLEX SUBUNIT 1
84164	ASCC2	ASCC2	HYPOTHETICAL PROTEIN DKFZP586O0223
10973	ASCC3	ASCC3	ACTIVATING SIGNAL COINTEGRATOR 1 COMPLEX SUBUNIT 3
429	ASCL1	ASCL1	ACHAETE-SCUTE COMPLEX-LIKE 1 (DROSOPHILA)
430	ASCL2	ASCL2	ACHAETE-SCUTE COMPLEX-LIKE 2 (DROSOPHILA)
56676	ASCL3	ASCL3	ACHAETE-SCUTE COMPLEX (DROSOPHILA) HOMOLOG-LIKE 3
55870	ASH1L	ASH1L	ASH1 (ABSENT, SMALL, OR HOMEOTIC)-LIKE (DROSOPHILA)
9070	ASH2L	ASH2L	ASH2 (ABSENT, SMALL, OR HOMEOTIC)-LIKE (DROSOPHILA)
171023	ASXL1	ASXL1	KIAA0978 PROTEIN
463	ATBF1	ATBF1	AT-BINDING TRANSCRIPTION FACTOR 1
466	ATF1	ATF1	ACTIVATING TRANSCRIPTION FACTOR 1
1386	ATF2	ATF2	ACTIVATING TRANSCRIPTION FACTOR 2
467	ATF3	ATF3	ACTIVATING TRANSCRIPTION FACTOR 3
468	ATF4	ATF4	ACTIVATING TRANSCRIPTION FACTOR 4 (TAX-RESPONSIVE ENHANCER ELEMENT B67)
22809	ATF5	ATF5	ACTIVATING TRANSCRIPTION FACTOR 5
22926	ATF6	ATF6	ACTIVATING TRANSCRIPTION FACTOR 6
11016	ATF7	ATF7	ACTIVATING TRANSCRIPTION FACTOR 7
55729	ATF7IP	ATF7IP	activating transcription factor 7 interacting protein
472	ATM	ATM	ATAXIA TELANGIECTASIA MUTATED (INCLUDES COMPLEMENTATION GROUPS A, C AND D)
474	ATOH1	ATOH1	ATONAL HOMOLOG 1 (DROSOPHILA)
546	ATRX	ATRX	ALPHA THALASSEMIA/MENTAL RETARDATION SYNDROME X-LINKED (RAD54 HOMOLOG, S. CEREVISIAE)
571	BACH1	BACH1	BTB AND CNC HOMOLOGY 1, BASIC LEUCINE ZIPPER TRANSCRIPTION FACTOR 1
60468	BACH2	BACH2	BTB AND CNC HOMOLOGY 1, BASIC LEUCINE ZIPPER TRANSCRIPTION FACTOR 2
579	BAPX1	BAPX1	BAGPIPE HOMEOBOX HOMOLOG 1 (DROSOPHILA)
580	BARD1	BARD1	BRCA1 ASSOCIATED RING DOMAIN 1
56751	BARHL1	BARHL1	BARH-LIKE 1 (DROSOPHILA)
343472	BARHL2	BARHL2	BARH-LIKE 2 (DROSOPHILA)
56033	BARX1	BARX1	BARH-LIKE HOMEOBOX 1
8538	BARX2	BARX2	BARH-LIKE HOMEOBOX 2
10538	BATF	BATF	BASIC LEUCINE ZIPPER TRANSCRIPTION FACTOR, ATF-LIKE
11177	BAZ1A	BAZ1A	BROMODOMAIN ADJACENT TO ZINC FINGER DOMAIN, 1A
9031	BAZ1B	BAZ1B	WILLIAMS-BEUREN SYNDROME CHROMOSOME REGION 10
29994	BAZ2B	BAZ2B	DKFZP434H071 PROTEIN
56987	BBX	BBX	BOBBY SOX HOMOLOG (DROSOPHILA)
53335	BCL11A	BCL11A	B-CELL CLL/LYMPHOMA 11A (ZINC FINGER PROTEIN)
64919	BCL11B	BCL11B	B-CELL CLL/LYMPHOMA 11B (ZINC FINGER PROTEIN)
602	BCL3	BCL3	B-CELL CLL/LYMPHOMA 3
604	BCL6	BCL6	B-CELL CLL/LYMPHOMA 6 (ZINC FINGER PROTEIN 51)
255877	BCL6B	BCL6B	B-CELL CLL/LYMPHOMA 6, MEMBER B (ZINC FINGER PROTEIN)
9774	BCLAF1	BCLAF1	BCL2-ASSOCIATED TRANSCRIPTION FACTOR 1
54880	BCOR	BCOR	BCL6 CO-REPRESSOR
55814	BDP1	BDP1	B DOUBLE PRIME 1, SUBUNIT OF RNA POLYMERASE III TRANSCRIPTION INITIATION FACTOR IIIB
8553	BHLHB2	BHLHB2	BASIC HELIX-LOOP-HELIX DOMAIN CONTAINING, CLASS B, 2
79365	BHLHB3	BHLHB3	BASIC HELIX-LOOP-HELIX DOMAIN CONTAINING, CLASS B, 3
168620	BHLHB8	BHLHB8	BASIC HELIX-LOOP-HELIX DOMAIN CONTAINING, CLASS B, 8
274	BIN1	BIN1	BRIDGING INTEGRATOR 1
8548	BLZF1	BLZF1	BASIC LEUCINE ZIPPER NUCLEAR FACTOR 1 (JEM-1)
646	BNC1	BNC1	BASONUCLIN 1
51027	BOLA1	BOLA1	BOLA-LIKE 1 (E. COLI)
552900	BOLA2	BOLA2	BOLA-LIKE 2 (E. COLI)
672	BRCA1	BRCA1	BREAST CANCER 1, EARLY ONSET
675	BRCA2	BRCA2	BREAST CANCER 2, EARLY ONSET
23774	BRD1	BRD1	BROMODOMAIN CONTAINING 1
29117	BRD7	BRD7	BROMODOMAIN CONTAINING 7
10902	BRD8	BRD8	BROMODOMAIN CONTAINING 8
676	BRDT	BRDT	BROMODOMAIN, TESTIS-SPECIFIC
2972	BRF1	BRF1	BRF1 HOMOLOG, SUBUNIT OF RNA POLYMERASE III TRANSCRIPTION INITIATION FACTOR IIIB (S. CEREVISIAE)
83990	BRIP1	BRIP1	BRCA1 INTERACTING PROTEIN C-TERMINAL HELICASE 1

7862	BRPF1	BRPF1	BROMODOMAIN AND PHD FINGER CONTAINING, 1
27154	BRPF3	BRPF3	BROMODOMAIN AND PHD FINGER CONTAINING, 3
54014	BRWD1	BRWD1	BROMODOMAIN AND WD REPEAT DOMAIN CONTAINING 1
9044	BTAF1	BTAF1	BTAF1 RNA POLYMERASE II, B-TFIID TRANSCRIPTION FACTOR-ASSOCIATED, 170KDA (MOT1 HOMOLOG, S. CEREVISIAE)
121551	BTBD11	BTBD11	BTB (POZ) DOMAIN CONTAINING 11
140685	BTBD4	BTBD4	BTB (POZ) DOMAIN CONTAINING 4
689	BTF3	BTF3	BASIC TRANSCRIPTION FACTOR 3
694	BTG1	BTG1	B-CELL TRANSLOCATION GENE 1, ANTI-PROLIFERATIVE
7832	BTG2	BTG2	BTG FAMILY, MEMBER 2
8896	BUD31	BUD31	G10 PROTEIN
9689	BZW1	BZW1	BASIC LEUCINE ZIPPER AND W2 DOMAINS 1
55320	C14orf106	C14ORF106	PUTATIVE PROTEIN P243 WHICH INTERACTS WITH TRANSCRIPTION FACTOR SP1
10438	C1D	C1D	NUCLEAR DNA-BINDING PROTEIN
127428	C1orf83	C1ORF83	CHROMOSOME 1 OPEN READING FRAME 83
140893	C20orf151	C20ORF151	CHROMOSOME 20 OPEN READING FRAME 151
56911	C21orf7	C21ORF7	CHROMOSOME 21 OPEN READING FRAME 7
6936	C2orf3	C2ORF3	CHROMOSOME 2 OPEN READING FRAME 3
57658	CALCOCO1	CALCOCO1	CALCIUM BINDING AND COILED-COIL DOMAIN 1
8092	CART1	CART1	CARTILAGE PAIRED-CLASS HOMEOPROTEIN 1
54897	CASZ1	CASZ1	CASTOR HOMOLOG 1, ZINC FINGER (DROSOPHILA)
9139	CBFA2T2	CBFA2T2	CORE-BINDING FACTOR, RUNT DOMAIN, ALPHA SUBUNIT 2; TRANSLOCATED TO, 2
863	CBFA2T3	CBFA2T3	CORE-BINDING FACTOR, RUNT DOMAIN, ALPHA SUBUNIT 2; TRANSLOCATED TO, 3
865	CBFB	CBFB	CORE-BINDING FACTOR, BETA SUBUNIT
11335	CBX3	CBX3	CHROMOBOX HOMOLOG 3 (HP1 GAMMA HOMOLOG, DROSOPHILA)
8535	CBX4	CBX4	CHROMOBOX HOMOLOG 4 (PC CLASS HOMOLOG, DROSOPHILA)
23466	CBX6	CBX6	CHROMOBOX HOMOLOG 6
23492	CBX7	CBX7	CHROMOBOX HOMOLOG 7
57332	CBX8	CBX8	CHROMOBOX HOMOLOG 8 (PC CLASS HOMOLOG, DROSOPHILA)
25819	CCRN4L	CCRN4L	CCR4 CARBON CATABOLITE REPRESSION 4-LIKE (S. CEREVISIAE)
988	CDC5L	CDC5L	CDC5 CELL DIVISION CYCLE 5-LIKE (S. POMBE)
1044	CDX1	CDX1	CAUDAL TYPE HOMEOBOX TRANSCRIPTION FACTOR 1
1045	CDX2	CDX2	CAUDAL TYPE HOMEOBOX TRANSCRIPTION FACTOR 2
1046	CDX4	CDX4	CAUDAL TYPE HOMEOBOX TRANSCRIPTION FACTOR 4
1050	CEBPA	CEBPA	CCAAT/ENHANCER BINDING PROTEIN (C/EBP), ALPHA
1051	CEBPB	CEBPB	CCAAT/ENHANCER BINDING PROTEIN (C/EBP), BETA
1052	CEBPD	CEBPD	CCAAT/ENHANCER BINDING PROTEIN (C/EBP), DELTA
1053	CEBPE	CEBPE	CCAAT/ENHANCER BINDING PROTEIN (C/EBP), EPSILON
1054	CEBPG	CEBPG	CCAAT/ENHANCER BINDING PROTEIN (C/EBP), GAMMA
10153	CEBPZ	CEBPZ	CCAAT/ENHANCER BINDING PROTEIN ZETA
1059	CENPB	CENPB	CENTROMERE PROTEIN B, 80KDA
116988	CENTG3	CENTG3	CENTAURIN, GAMMA 3
11064	CEP110	CEP110	CENTROSOMAL PROTEIN 110KDA
10036	CHAF1A	CHAF1A	CHROMATIN ASSEMBLY FACTOR 1, SUBUNIT A (P150)
1105	CHD1	CHD1	CHROMODOMAIN HELICASE DNA BINDING PROTEIN 1
1106	CHD2	CHD2	CHROMODOMAIN HELICASE DNA BINDING PROTEIN 2
1108	CHD4	CHD4	CHROMODOMAIN HELICASE DNA BINDING PROTEIN 4
26038	CHD5	CHD5	CHROMODOMAIN HELICASE DNA BINDING PROTEIN 5
55636	CHD7	CHD7	CHROMODOMAIN HELICASE DNA BINDING PROTEIN 7
57680	CHD8	CHD8	CHROMODOMAIN HELICASE DNA BINDING PROTEIN 8
1112	CHES1	CHES1	CHECKPOINT SUPPRESSOR 1
91612	CHURC1	CHURC1	CHURCHILL DOMAIN CONTAINING 1
338917	CHX10	CHX10	CEH-10 HOMEODOMAIN CONTAINING HOMOLOG (C. ELEGANS)
23152	CIC	CIC	CAPICUA HOMOLOG (DROSOPHILA)
4261	CIITA	CIITA	CLASS II, MAJOR HISTOCOMPATIBILITY COMPLEX, TRANSACTIVATOR
4435	CITED1	CITED1	CBP/P300-INTERACTING TRANSACTIVATOR, WITH GLU/ASP-RICH CARBOXY-TERMINAL DOMAIN, 1
10370	CITED2	CITED2	CBP/P300-INTERACTING TRANSACTIVATOR, WITH GLU/ASP-RICH CARBOXY-TERMINAL DOMAIN, 2
163732	CITED4	CITED4	CBP/P300-INTERACTING TRANSACTIVATOR, WITH GLU/ASP-RICH CARBOXY-TERMINAL DOMAIN, 4
9575	CLOCK	CLOCK	CLOCK HOMOLOG (MOUSE)
4848	CNOT2	CNOT2	CCR4-NOT TRANSCRIPTION COMPLEX, SUBUNIT 2
4849	CNOT3	CNOT3	CCR4-NOT TRANSCRIPTION COMPLEX, SUBUNIT 3
4850	CNOT4	CNOT4	CCR4-NOT TRANSCRIPTION COMPLEX, SUBUNIT 4
57472	CNOT6	CNOT6	CCR4-NOT TRANSCRIPTION COMPLEX, SUBUNIT 6
246175	CNOT6L	CNOT6L	CCR4-NOT transcription complex, subunit 6-like
29883	CNOT7	CNOT7	CCR4-NOT TRANSCRIPTION COMPLEX, SUBUNIT 7
9337	CNOT8	CNOT8	CCR4-NOT TRANSCRIPTION COMPLEX, SUBUNIT 8
25920	COBRA1	COBRA1	COFACTOR OF BRCA1
8161	COIL	COIL	COILIN
9318	COPS2	COPS2	COP9 CONSTITUTIVE PHOTOMORPHOGENIC HOMOLOG SUBUNIT 2 (ARABIDOPSIS)
10987	COPS5	COPS5	COP9 CONSTITUTIVE PHOTOMORPHOGENIC HOMOLOG SUBUNIT 5 (ARABIDOPSIS)
1382	CRABP2	CRABP2	CELLULAR RETINOIC ACID BINDING PROTEIN 2
57585	CRAMP1L	CRAMP1L	CRM, CRAMPED-LIKE (DROSOPHILA)
1385	CREB1	CREB1	CAMP RESPONSIVE ELEMENT BINDING PROTEIN 1
10488	CREB3	CREB3	CAMP RESPONSIVE ELEMENT BINDING PROTEIN 3
90993	CREB3L1	CREB3L1	CAMP RESPONSIVE ELEMENT BINDING PROTEIN 3-LIKE 1
84699	CREB3L3	CREB3L3	CAMP RESPONSIVE ELEMENT BINDING PROTEIN 3-LIKE 3
148327	CREB3L4	CREB3L4	CAMP RESPONSIVE ELEMENT BINDING PROTEIN 3-LIKE 4
9586	CREB5	CREB5	CAMP RESPONSIVE ELEMENT BINDING PROTEIN 5
1387	CREBBP	CREBBP	CREB BINDING PROTEIN (RUBINSTEIN-TAYBI SYNDROME)
1388	CREBL1	CREBL1	CAMP RESPONSIVE ELEMENT BINDING PROTEIN-LIKE 1
1389	CREBL2	CREBL2	CAMP RESPONSIVE ELEMENT BINDING PROTEIN-LIKE 2
8804	CREG1	CREG1	CELLULAR REPRESSOR OF E1A-STIMULATED GENES 1
1390	CREM	CREM	CAMP RESPONSIVE ELEMENT MODULATOR
9282	CRSP2	CRSP2	COFACTOR REQUIRED FOR SP1 TRANSCRIPTIONAL ACTIVATION, SUBUNIT 2, 150KDA
9439	CRSP3	CRSP3	COFACTOR REQUIRED FOR SP1 TRANSCRIPTIONAL ACTIVATION, SUBUNIT 3, 130KDA
9440	CRSP6	CRSP6	COFACTOR REQUIRED FOR SP1 TRANSCRIPTIONAL ACTIVATION, SUBUNIT 6, 77KDA
9441	CRSP7	CRSP7	COFACTOR REQUIRED FOR SP1 TRANSCRIPTIONAL ACTIVATION, SUBUNIT 7, 70KDA
9442	CRSP8	CRSP8	COFACTOR REQUIRED FOR SP1 TRANSCRIPTIONAL ACTIVATION, SUBUNIT 8, 34KDA
9443	CRSP9	CRSP9	COFACTOR REQUIRED FOR SP1 TRANSCRIPTIONAL ACTIVATION, SUBUNIT 9, 33KDA
23373	CRTC1	CRTC1	CREB REGULATED TRANSCRIPTION COACTIVATOR 1
1407	CRY1	CRY1	CRYPTOCHROME 1 (PHOTOLYASE-LIKE)
8531	CSDA	CSDA	COLD SHOCK DOMAIN PROTEIN A
27254	CSDC2	CSDC2	COLD SHOCK DOMAIN CONTAINING C2, RNA BINDING
7812	CSDE1	CSDE1	COLD SHOCK DOMAIN CONTAINING E1, RNA-BINDING
1466	CSRP2	CSRP2	CYSTEINE AND GLYCINE-RICH PROTEIN 2
8048	CSRP3	CSRP3	CYSTEINE AND GLYCINE-RICH PROTEIN 3 (CARDIAC LIM PROTEIN)
1487	CTBP1	CTBP1	C-TERMINAL BINDING PROTEIN 1
10664	CTCF	CTCF	CCCTC-BINDING FACTOR (ZINC FINGER PROTEIN)
140690	CTCFL	CTCFL	CCCTC-BINDING FACTOR (ZINC FINGER PROTEIN)-LIKE
9150	CTDP1	CTDP1	CTD (CARBOXY-TERMINAL DOMAIN, RNA POLYMERASE II, POLYPEPTIDE A) PHOSPHATASE, SUBUNIT 1
10217	CTDSPL	CTDSPL	CTD (CARBOXY-TERMINAL DOMAIN, RNA POLYMERASE II, POLYPEPTIDE A) SMALL PHOSPHATASE-LIKE
1499	CTNNB1	CTNNB1	CATENIN (CADHERIN-ASSOCIATED PROTEIN), BETA 1, 88KDA
56998	CTNNBIP1	CTNNBIP1	CATENIN, BETA INTERACTING PROTEIN 1
56259	CTNNBL1	CTNNBL1	CATENIN, BETA LIKE 1
1523	CUTL1	CUTL1	CUT-LIKE 1, CCAAT DISPLACEMENT PROTEIN (DROSOPHILA)
23316	CUTL2	CUTL2	CUT-LIKE 2 (DROSOPHILA)
30827	CXXC1	CXXC1	CXXC FINGER 1 (PHD DOMAIN)
1601	DAB2	DAB2	DISABLED HOMOLOG 2, MITOGEN-RESPONSIVE PHOSPHOPROTEIN (DROSOPHILA)
1602	DACH1	DACH1	DACHSHUND HOMOLOG 1 (DROSOPHILA)
117154	DACH2	DACH2	DACHSHUND HOMOLOG 2 (DROSOPHILA)
1616	DAXX	DAXX	DEATH-ASSOCIATED PROTEIN 6
9802	DAZAP2	DAZAP2	DAZ ASSOCIATED PROTEIN 2
1628	DBP	DBP	D SITE OF ALBUMIN PROMOTER (ALBUMIN D-BOX) BINDING PROTEIN
55802	DCP1A	DCP1A	DCP1 DECAPPING ENZYME HOMOLOG A (S. CEREVISIAE)
1642	DDB1	DDB1	DAMAGE-SPECIFIC DNA BINDING PROTEIN 1, 127KDA
1643	DDB2	DDB2	DAMAGE-SPECIFIC DNA BINDING PROTEIN 2, 48KDA
1649	DDIT3	DDIT3	DNA-DAMAGE-INDUCIBLE TRANSCRIPT 3
11218	DDX20	DDX20	DEAD (ASP-GLU-ALA-ASP) BOX POLYPEPTIDE 20
1655	DDX5	DDX5	DEAD (ASP-GLU-ALA-ASP) BOX POLYPEPTIDE 5
79039	DDX54	DDX54	DEAD (ASP-GLU-ALA-ASP) BOX POLYPEPTIDE 54
10522	DEAF1	DEAF1	DEFORMED EPIDERMAL AUTOREGULATORY FACTOR 1 (DROSOPHILA)
9191	DEDD	DEDD	DEATH EFFECTOR DOMAIN CONTAINING
162989	DEDD2	DEDD2	DEATH EFFECTOR DOMAIN CONTAINING 2
7913	DEK	DEK	DEK ONCOGENE (DNA BINDING)
10260	DENND4A	DENND4A	DENN/MADD DOMAIN CONTAINING 4A
9785	DHX38	DHX38	DEAH (ASP-GLU-ALA-HIS) BOX POLYPEPTIDE 38
11083	DIDO1	DIDO1	DEATH INDUCER-OBLITERATOR 1
22982	DIP2C	DIP2C	DIP2 DISCO-INTERACTING PROTEIN 2 HOMOLOG C (DROSOPHILA)
1745	DLX1	DLX1	DISTAL-LESS HOMEOBOX 1
1746	DLX2	DLX2	DISTAL-LESS HOMEOBOX 2

1747	DLX3	DLX3	DISTAL-LESS HOMEOBOX 3
1748	DLX4	DLX4	DISTAL-LESS HOMEOBOX 4
1749	DLX5	DLX5	DISTAL-LESS HOMEOBOX 5
1750	DLX6	DLX6	DISTAL-LESS HOMEOBOX 6
55929	DMAP1	DMAP1	DNA METHYLTRANSFERASE 1 ASSOCIATED PROTEIN 1
127343	DMBX1	DMBX1	DIENCEPHALON/MESENCEPHALON HOMEOBOX 1
1761	DMRT1	DMRT1	DOUBLESEX AND MAB-3 RELATED TRANSCRIPTION FACTOR 1
10655	DMRT2	DMRT2	DOUBLESEX AND MAB-3 RELATED TRANSCRIPTION FACTOR 2
58524	DMRT3	DMRT3	DOUBLESEX AND MAB-3 RELATED TRANSCRIPTION FACTOR 3
63951	DMRTA1	DMRTA1	DMRT-LIKE FAMILY A1
63948	DMRTB1	DMRTB1	DMRT-LIKE FAMILY B WITH PROLINE-RICH C-TERMINAL, 1
63946	DMRTC2	DMRTC2	DMRT-LIKE FAMILY C2
9988	DMTF1	DMTF1	CYCLIN D BINDING MYB-LIKE TRANSCRIPTION FACTOR 1
1786	DNMT1	DNMT1	DNA (CYTOSINE-5-)-METHYLTRANSFERASE 1
1788	DNMT3A	DNMT3A	DNA (CYTOSINE-5-)-METHYLTRANSFERASE 3 ALPHA
29947	DNMT3L	DNMT3L	DNA (CYTOSINE-5-)-METHYLTRANSFERASE 3-LIKE
84444	DOT1L	DOT1L	DOT1-LIKE, HISTONE H3 METHYLTRANSFERASE (S. CEREVISIAE)
8193	DPF1	DPF1	D4, ZINC AND DOUBLE PHD FINGERS FAMILY 1
5977	DPF2	DPF2	D4, ZINC AND DOUBLE PHD FINGERS FAMILY 2
8110	DPF3	DPF3	D4, ZINC AND DOUBLE PHD FINGERS, FAMILY 3
1810	DR1	DR1	DOWN-REGULATOR OF TRANSCRIPTION 1, TBP-BINDING (NEGATIVE COFACTOR 2)
10589	DRAP1	DRAP1	DR1-ASSOCIATED PROTEIN 1 (NEGATIVE COFACTOR 2 ALPHA)
1840	DTX1	DTX1	DELTEX HOMOLOG 1 (DROSOPHILA)
26582	DUX3	DUX3	DOUBLE HOMEOBOX, 3
22947	DUX4	DUX4	DOUBLE HOMEOBOX, 4
1869	E2F1	E2F1	E2F TRANSCRIPTION FACTOR 1
1870	E2F2	E2F2	E2F TRANSCRIPTION FACTOR 2
1871	E2F3	E2F3	E2F TRANSCRIPTION FACTOR 3
1874	E2F4	E2F4	E2F TRANSCRIPTION FACTOR 4, P107/P130-BINDING
1875	E2F5	E2F5	E2F TRANSCRIPTION FACTOR 5, P130-BINDING
1876	E2F6	E2F6	E2F TRANSCRIPTION FACTOR 6
79733	E2F8	E2F8	E2F TRANSCRIPTION FACTOR 8
1877	E4F1	E4F1	E4F TRANSCRIPTION FACTOR 1
85403	EAF1	EAF1	ELL ASSOCIATED FACTOR 1
1879	EBF	EBF	EARLY B-CELL FACTOR
64641	EBF2	EBF2	EARLY B-CELL FACTOR 2
253738	EBF3	EBF3	EARLY B-CELL FACTOR 3
57593	RP5-860F19.3	EBF4	early B-cell factor 4
11319	ECD	ECD	ECDYSONELESS HOMOLOG (DROSOPHILA)
8721	EDF1	EDF1	ENDOTHELIAL DIFFERENTIATION-RELATED FACTOR 1
8726	EED	EED	EMBRYONIC ECTODERM DEVELOPMENT
1915	EEF1A1	EEF1A1	EUKARYOTIC TRANSLATION ELONGATION FACTOR 1 ALPHA 1
1958	EGR1	EGR1	EARLY GROWTH RESPONSE 1
1959	EGR2	EGR2	EARLY GROWTH RESPONSE 2 (KROX-20 HOMOLOG, DROSOPHILA)
1960	EGR3	EGR3	EARLY GROWTH RESPONSE 3
1961	EGR4	EGR4	EARLY GROWTH RESPONSE 4
26298	EHF	EHF	ETS HOMOLOGOUS FACTOR
79813	EHMT1	EHMT1	EUCHROMATIC HISTONE-LYSINE N-METHYLTRANSFERASE 1
10919	EHMT2	EHMT2	EUCHROMATIC HISTONE-LYSINE N-METHYLTRANSFERASE 2
5610	EIF2AK2	EIF2AK2	EUKARYOTIC TRANSLATION INITIATION FACTOR 2-ALPHA KINASE 2
9669	EIF5B	EIF5B	EUKARYOTIC TRANSLATION INITIATION FACTOR 5B
1993	ELAVL2	ELAVL2	ELAV (EMBRYONIC LETHAL, ABNORMAL VISION, DROSOPHILA)-LIKE 2 (HU ANTIGEN B)
1997	ELF1	ELF1	E74-LIKE FACTOR 1 (ETS DOMAIN TRANSCRIPTION FACTOR)
1998	ELF2	ELF2	E74-LIKE FACTOR 2 (ETS DOMAIN TRANSCRIPTION FACTOR)
1999	ELF3	ELF3	E74-LIKE FACTOR 3 (ETS DOMAIN TRANSCRIPTION FACTOR, EPITHELIAL-SPECIFIC)
2000	ELF4	ELF4	E74-LIKE FACTOR 4 (ETS DOMAIN TRANSCRIPTION FACTOR)
2001	ELF5	ELF5	E74-LIKE FACTOR 5 (ETS DOMAIN TRANSCRIPTION FACTOR)
2002	ELK1	ELK1	ELK1, MEMBER OF ETS ONCOGENE FAMILY
2004	ELK3	ELK3	ELK3, ETS-DOMAIN PROTEIN (SRF ACCESSORY PROTEIN 2)
2005	ELK4	ELK4	ELK4, ETS-DOMAIN PROTEIN (SRF ACCESSORY PROTEIN 1)
8178	ELL	ELL	ELONGATION FACTOR RNA POLYMERASE II
22936	ELL2	ELL2	ELONGATION FACTOR, RNA POLYMERASE II, 2
80237	ELL3	ELL3	ELONGATION FACTOR RNA POLYMERASE II-LIKE 3
2016	EMX1	EMX1	EMPTY SPIRACLES HOMOLOG 1 (DROSOPHILA)
2018	EMX2	EMX2	EMPTY SPIRACLES HOMOLOG 2 (DROSOPHILA)
2019	EN1	EN1	ENGRAILED HOMOLOG 1
2020	EN2	EN2	ENGRAILED HOMOLOG 2
2023	ENO1	ENO1	ENOLASE 1, (ALPHA)
8320	EOMES	EOMES	EOMESODERMIN HOMOLOG (XENOPUS LAEVIS)
2033	EP300	EP300	E1A BINDING PROTEIN P300
57634	EP400	EP400	TRINUCLEOTIDE REPEAT CONTAINING 12
2034	EPAS1	EPAS1	ENDOTHELIAL PAS DOMAIN PROTEIN 1
80314	EPC1	EPC1	ENHANCER OF POLYCOMB HOMOLOG 1 (DROSOPHILA)
2068	ERCC2	ERCC2	EXCISION REPAIR CROSS-COMPLEMENTING RODENT REPAIR DEFICIENCY, COMPLEMENTATION GROUP 2 (XERODERMA PIGMENTOSUM D)
2071	ERCC3	ERCC3	EXCISION REPAIR CROSS-COMPLEMENTING RODENT REPAIR DEFICIENCY, COMPLEMENTATION GROUP 3 (XERODERMA PIGMENTOSUM GROUP B COMPLEMEN
1161	ERCC8	ERCC8	EXCISION REPAIR CROSS-COMPLEMENTING RODENT REPAIR DEFICIENCY, COMPLEMENTATION GROUP 8
2077	ERF	ERF	ETS2 REPRESSOR FACTOR
2078	ERG	ERG	V-ETS ERYTHROBLASTOSIS VIRUS E26 ONCOGENE LIKE (AVIAN)
9700	ESPL1	ESPL1	EXTRA SPINDLE POLES LIKE 1 (S. CEREVISIAE)
2099	ESR1	ESR1	ESTROGEN RECEPTOR 1
2100	ESR2	ESR2	ESTROGEN RECEPTOR 2 (ER BETA)
2101	ESRRA	ESRRA	ESTROGEN-RELATED RECEPTOR ALPHA
2103	ESRRB	ESRRB	ESTROGEN-RELATED RECEPTOR BETA
2104	ESRRG	ESRRG	ESTROGEN-RELATED RECEPTOR GAMMA
80712	ESX1	ESX1	EXTRAEMBRYONIC, SPERMATOGENESIS, HOMEOBOX 1 HOMOLOG (MOUSE)
2113	ETS1	ETS1	V-ETS ERYTHROBLASTOSIS VIRUS E26 ONCOGENE HOMOLOG 1 (AVIAN)
2114	ETS2	ETS2	V-ETS ERYTHROBLASTOSIS VIRUS E26 ONCOGENE HOMOLOG 2 (AVIAN)
2115	ETV1	ETV1	ETS VARIANT GENE 1
2116	ETV2	ETV2	ETS VARIANT GENE 2
2117	ETV3	ETV3	ETS VARIANT GENE 3
2118	ETV4	ETV4	ETS VARIANT GENE 4 (E1A ENHANCER BINDING PROTEIN, E1AF)
2119	ETV5	ETV5	ETS VARIANT GENE 5 (ETS-RELATED MOLECULE)
2120	ETV6	ETV6	ETS VARIANT GENE 6 (TEL ONCOGENE)
51513	ETV7	ETV7	ETS TRANSCRIPTION FACTOR TEL-2B
2122	EVI1	EVI1	ECOTROPIC VIRAL INTEGRATION SITE 1
4197	MDS1	MDS1	myelodysplasia syndrome 1
2128	EVX1	EVX1	EVE, EVEN-SKIPPED HOMEOBOX HOMOLOG 1 (DROSOPHILA)
2130	EWSR1	EWSR1	EWING SARCOMA BREAKPOINT REGION 1
2139	EYA2	EYA2	EYES ABSENT HOMOLOG 2 (DROSOPHILA)
2140	EYA3	EYA3	EYES ABSENT HOMOLOG 3 (DROSOPHILA)
2070	EYA4	EYA4	EYES ABSENT HOMOLOG 4 (DROSOPHILA)
2145	EZH1	EZH1	ENHANCER OF ZESTE HOMOLOG 1 (DROSOPHILA)
2146	EZH2	EZH2	ENHANCER OF ZESTE HOMOLOG 2 (DROSOPHILA)
9214	FAIM3	FAIM3	FAS APOPTOTIC INHIBITORY MOLECULE 3
2186	FALZ	FALZ	FETAL ALZHEIMER ANTIGEN
92565	FANK1	FANK1	FIBRONECTIN TYPE III AND ANKYRIN REPEAT DOMAINS 1
84678	FBXL10	FBXL10	F-BOX AND LEUCINE-RICH REPEAT PROTEIN 10
22992	FBXL11	FBXL11	F-BOX AND LEUCINE-RICH REPEAT PROTEIN 11
54620	FBXL19	FBXL19	F-BOX AND LEUCINE-RICH REPEAT PROTEIN 19
25793	FBXO7	FBXO7	F-BOX PROTEIN 7
54738	FEV	FEV	FEV (ETS ONCOGENE FAMILY)
2274	FHL2	FHL2	FOUR AND A HALF LIM DOMAINS 2
9457	FHL5	FHL5	FOUR AND A HALF LIM DOMAINS 5
344018	FIGLA	FIGLA	FACTOR IN THE GERMLINE ALPHA
84922	FLJ14768	FIZ1	FLT3-interacting zinc finger 1
2307	FKHL18	FKHL18	FORKHEAD-LIKE 18 (DROSOPHILA)
64105	FKSG14	FKSG14	LEUCINE ZIPPER PROTEIN FKSG14
2313	FLI1	FLI1	FRIEND LEUKEMIA VIRUS INTEGRATION 1
79998	ANKRD53	FLJ12056	HYPOTHETICAL PROTEIN FLJ12056
2332	FMR1	FMR1	FRAGILE X MENTAL RETARDATION 1
2353	FOS	FOS	V-FOS FBJ MURINE OSTEOSARCOMA VIRAL ONCOGENE HOMOLOG
2354	FOSB	FOSB	FBJ MURINE OSTEOSARCOMA VIRAL ONCOGENE HOMOLOG B
8061	FOSL1	FOSL1	FOS-LIKE ANTIGEN 1
2355	FOSL2	FOSL2	FOS-LIKE ANTIGEN 2
3169	FOXA1	FOXA1	FORKHEAD BOX A1
3170	FOXA2	FOXA2	FORKHEAD BOX A2
3171	FOXA3	FOXA3	FORKHEAD BOX A3
27023	FOXB1	FOXB1	FORKHEAD BOX B1
2296	FOXC1	FOXC1	FORKHEAD BOX C1

2303	FOXC2	FOXC2	FORKHEAD BOX C2 (MFH-1, MESENCHYME FORKHEAD 1)
2297	FOXD1	FOXD1	FORKHEAD BOX D1
2306	FOXD2	FOXD2	FORKHEAD BOX D2
27022	FOXD3	FOXD3	FORKHEAD BOX D3
2298	FOXD4	FOXD4	FORKHEAD BOX D4
200350	FOXD4L1	FOXD4L1	FORKHEAD BOX D4-LIKE 1
349334	FOXD4L4	FOXD4L4	FORKHEAD BOX D4-LIKE 4
2304	FOXE1	FOXE1	FORKHEAD BOX E1 (THYROID TRANSCRIPTION FACTOR 2)
2301	FOXE3	FOXE3	FORKHEAD BOX E3
2294	FOXF1	FOXF1	FORKHEAD BOX F1
2295	FOXF2	FOXF2	FORKHEAD BOX F2
2291	FOXG1A	FOXG1A	FORKHEAD BOX G1A
2290	FOXG1B	FOXG1B	FORKHEAD BOX G1B
2292	FOXG1C	FOXG1C	FORKHEAD BOX G1C
8928	FOXH1	FOXH1	FORKHEAD BOX H1
2299	FOXI1	FOXI1	FORKHEAD BOX I1
2302	FOXJ1	FOXJ1	FORKHEAD BOX J1
55810	FOXJ2	FOXJ2	FORKHEAD BOX J2
22887	FOXJ3	FOXJ3	FORKHEAD BOX J3
3607	FOXK2	FOXK2	FORKHEAD BOX K2
2300	FOXL1	FOXL1	FORKHEAD BOX L1
668	FOXL2	FOXL2	FORKHEAD BOX L2
2305	FOXM1	FOXM1	FORKHEAD BOX M1
8456	FOXN1	FOXN1	FORKHEAD BOX N1
121643	FOXN4	FOXN4	FORKHEAD BOX N4
2308	FOXO1A	FOXO1A	FORKHEAD BOX O1A (RHABDOMYOSARCOMA)
2309	FOXO3A	FOXO3A	FORKHEAD BOX O3A
27086	FOXP1	FOXP1	FORKHEAD BOX P1
93986	FOXP2	FOXP2	TRINUCLEOTIDE REPEAT CONTAINING 10
50943	FOXP3	FOXP3	FORKHEAD BOX P3
116113	FOXP4	FOXP4	FORKHEAD BOX P4
94234	FOXQ1	FOXQ1	FORKHEAD BOX Q1
8880	FUBP1	FUBP1	FAR UPSTREAM ELEMENT (FUSE) BINDING PROTEIN 1
8939	FUBP3	FUBP3	FAR UPSTREAM ELEMENT (FUSE) BINDING PROTEIN 3
2521	FUS	FUS	FUSION (INVOLVED IN T(12;16) IN MALIGNANT LIPOSARCOMA)
10772	FUSIP1	FUSIP1	FUS INTERACTING PROTEIN (SERINE/ARGININE-RICH) 1
8087	FXR1	FXR1	FRAGILE X MENTAL RETARDATION, AUTOSOMAL HOMOLOG 1
9513	FXR2	FXR2	FRAGILE X MENTAL RETARDATION, AUTOSOMAL HOMOLOG 2
2551	GABPA	GABPA	GA BINDING PROTEIN TRANSCRIPTION FACTOR, ALPHA SUBUNIT 60KDA
2553	GABPB2	GABPB2	GA BINDING PROTEIN TRANSCRIPTION FACTOR, BETA SUBUNIT 1, 53KDA
2623	GATA1	GATA1	GATA BINDING PROTEIN 1 (GLOBIN TRANSCRIPTION FACTOR 1)
2624	GATA2	GATA2	GATA BINDING PROTEIN 2
2625	GATA3	GATA3	GATA BINDING PROTEIN 3
2626	GATA4	GATA4	GATA BINDING PROTEIN 4
140628	GATA5	GATA5	GATA BINDING PROTEIN 5
2627	GATA6	GATA6	GATA BINDING PROTEIN 6
57798	GATAD1	GATAD1	GATA ZINC FINGER DOMAIN CONTAINING 1
54815	GATAD2A	GATAD2A	GATA ZINC FINGER DOMAIN CONTAINING 2A
57459	GATAD2B	GATAD2B	GATA ZINC FINGER DOMAIN CONTAINING 2B
2637	GBX2	GBX2	GASTRULATION BRAIN HOMEOBOX 2
94104	C21orf66	GCFC	GC-RICH SEQUENCE DNA-BINDING FACTOR CANDIDATE
8521	GCM1	GCM1	GLIAL CELLS MISSING HOMOLOG 1 (DROSOPHILA)
9247	GCM2	GCM2	GLIAL CELLS MISSING HOMOLOG 2 (DROSOPHILA)
2672	GFI1	GFI1	GROWTH FACTOR INDEPENDENT 1
8328	GFI1B	GFI1B	GROWTH FACTOR INDEPENDENT 1B (POTENTIAL REGULATOR OF CDKN1A, TRANSLOCATED IN CML)
92283	GIOT-1	GIOT-1	GONADOTROPIN INDUCIBLE TRANSCRIPTION REPRESSOR 1
2735	GLI1	GLI1	GLIOMA-ASSOCIATED ONCOGENE HOMOLOG 1 (ZINC FINGER PROTEIN)
2736	GLI2	GLI2	GLI-KRUPPEL FAMILY MEMBER GLI2
2737	GLI3	GLI3	GLI-KRUPPEL FAMILY MEMBER GLI3 (GREIG CEPHALOPOLYSYNDACTYLY SYNDROME)
2738	GLI4	GLI4	GLI-KRUPPEL FAMILY MEMBER GLI4
148979	GLIS1	GLIS1	GLIS FAMILY ZINC FINGER 1
84662	GLIS2	GLIS2	GLIS FAMILY ZINC FINGER 2
169792	GLIS3	GLIS3	GLIS FAMILY ZINC FINGER 3
10691	GMEB1	GMEB1	GLUCOCORTICOID MODULATORY ELEMENT BINDING PROTEIN 1
26205	GMEB2	GMEB2	GLUCOCORTICOID MODULATORY ELEMENT BINDING PROTEIN 2
54856	GON4L	GON4L	GON-4-LIKE (C.ELEGANS)
29841	GRHL1	GRHL1	GRAINYHEAD-LIKE 1 (DROSOPHILA)
2909	GRLF1	GRLF1	GLUCOCORTICOID RECEPTOR DNA BINDING FACTOR 1
145258	GSC	GSC	GOOSECOID
2928	GSCL	GSCL	GOOSECOID-LIKE
219409	GSH1	GSH1	GS HOMEOBOX 1
170825	GSH2	GSH2	GS HOMEOBOX 2
2957	GTF2A1	GTF2A1	GENERAL TRANSCRIPTION FACTOR IIA, 1, 19/37KDA
2958	GTF2A2	GTF2A2	GENERAL TRANSCRIPTION FACTOR IIA, 2, 12KDA
2959	GTF2B	GTF2B	GENERAL TRANSCRIPTION FACTOR IIB
2960	GTF2E1	GTF2E1	GENERAL TRANSCRIPTION FACTOR IIE, POLYPEPTIDE 1, ALPHA 56KDA
2961	GTF2E2	GTF2E2	GENERAL TRANSCRIPTION FACTOR IIE, POLYPEPTIDE 2, BETA 34KDA
2962	GTF2F1	GTF2F1	GENERAL TRANSCRIPTION FACTOR IIF, POLYPEPTIDE 1, 74KDA
2963	GTF2F2	GTF2F2	GENERAL TRANSCRIPTION FACTOR IIF, POLYPEPTIDE 2, 30KDA
2965	GTF2H1	GTF2H1	GENERAL TRANSCRIPTION FACTOR IIH, POLYPEPTIDE 1, 62KDA
2966	GTF2H2	GTF2H2	GENERAL TRANSCRIPTION FACTOR IIH, POLYPEPTIDE 2, 44KDA
2967	GTF2H3	GTF2H3	GENERAL TRANSCRIPTION FACTOR IIH, POLYPEPTIDE 3, 34KDA
2968	GTF2H4	GTF2H4	GENERAL TRANSCRIPTION FACTOR IIH, POLYPEPTIDE 4, 52KDA
2969	GTF2I	GTF2I	GENERAL TRANSCRIPTION FACTOR II, I
9569	GTF2IRD1	GTF2IRD1	GTF2I REPEAT DOMAIN CONTAINING 1
2971	GTF3A	GTF3A	GENERAL TRANSCRIPTION FACTOR IIIA
2975	GTF3C1	GTF3C1	GENERAL TRANSCRIPTION FACTOR IIIC, POLYPEPTIDE 1, ALPHA 220KDA
2976	GTF3C2	GTF3C2	GENERAL TRANSCRIPTION FACTOR IIIC, POLYPEPTIDE 2, BETA 110KDA
9330	GTF3C3	GTF3C3	GENERAL TRANSCRIPTION FACTOR IIIC, POLYPEPTIDE 3, 102KDA
9329	GTF3C4	GTF3C4	GENERAL TRANSCRIPTION FACTOR IIIC, POLYPEPTIDE 4, 90KDA
9328	GTF3C5	GTF3C5	GENERAL TRANSCRIPTION FACTOR IIIC, POLYPEPTIDE 5, 63KDA
3005	H1F0	H1F0	H1 HISTONE FAMILY, MEMBER 0
8971	H1FX	H1FX	H1 HISTONE FAMILY, MEMBER X
8347	HIST1H2BC	H3/O	HISTONE 1, H2BD
3017	HIST1H2BD	H3/O	HISTONE 1, H2BD
8344	HIST1H2BE	H3/O	HISTONE 1, H2BD
8339	HIST1H2BG	H3/O	HISTONE 1, H2BD
22927	HABP4	HABP4	HYALURONAN BINDING PROTEIN 4
9421	HAND1	HAND1	HEART AND NEURAL CREST DERIVATIVES EXPRESSED 1
9464	HAND2	HAND2	HEART AND NEURAL CREST DERIVATIVES EXPRESSED 2
10456	HAX1	HAX1	HCLS1 ASSOCIATED PROTEIN X-1
26959	HBP1	HBP1	HMG-BOX TRANSCRIPTION FACTOR 1
51773	HBXAP	HBXAP	HEPATITIS B VIRUS X ASSOCIATED PROTEIN
3054	HCFC1	HCFC1	HOST CELL FACTOR C1 (VP16-ACCESSORY PROTEIN)
3059	HCLS1	HCLS1	HEMATOPOIETIC CELL-SPECIFIC LYN SUBSTRATE 1
3064	HD	HD	HUNTINGTIN (HUNTINGTON DISEASE)
3065	HDAC1	HDAC1	HISTONE DEACETYLASE 1
83933	HDAC10	HDAC10	HISTONE DEACETYLASE 10
79885	HDAC11	HDAC11	HISTONE DEACETYLASE 11
3066	HDAC2	HDAC2	HISTONE DEACETYLASE 2
8841	HDAC3	HDAC3	HISTONE DEACETYLASE 3
9759	HDAC4	HDAC4	HISTONE DEACETYLASE 4
10014	HDAC5	HDAC5	HISTONE DEACETYLASE 5
51564	HDAC7A	HDAC7A	HISTONE DEACETYLASE 7A
55869	HDAC8	HDAC8	HISTONE DEACETYLASE 8
9734	HDAC9	HDAC9	HISTONE DEACETYLASE 9
3068	HDGF	HDGF	HEPATOMA-DERIVED GROWTH FACTOR (HIGH-MOBILITY GROUP PROTEIN 1-LIKE)
84717	HDGF2	HDGF2	HEPATOMA-DERIVED GROWTH FACTOR-RELATED PROTEIN 2
139324	CXorf43	HDX	highly divergent homeobox
9931	HELZ	HELZ	HELICASE WITH ZINC FINGER
3280	HES1	HES1	HAIRY AND ENHANCER OF SPLIT 1, (DROSOPHILA)
54626	HES2	HES2	HAIRY AND ENHANCER OF SPLIT 2 (DROSOPHILA)
57801	HES4	HES4	HAIRY AND ENHANCER OF SPLIT 4 (DROSOPHILA)
55502	HES6	HES6	HAIRY AND ENHANCER OF SPLIT 6 (DROSOPHILA)
84667	HES7	HES7	HAIRY AND ENHANCER OF SPLIT 7 (DROSOPHILA)
8820	HESX1	HESX1	HOMEOBOX, ES CELL EXPRESSED 1
10614	HEXIM1	HEXIM1	HEXAMETHYLENE BIS-ACETAMIDE INDUCIBLE 1
23462	HEY1	HEY1	HAIRY/ENHANCER-OF-SPLIT RELATED WITH YRPW MOTIF 1
23493	HEY2	HEY2	HAIRY/ENHANCER-OF-SPLIT RELATED WITH YRPW MOTIF 2

26508	HEYL	HEYL	HAIRY/ENHANCER-OF-SPLIT RELATED WITH YRPW MOTIF-LIKE
3087	HHEX	HHEX	HOMEOBOX, HEMATOPOIETICALLY EXPRESSED
3090	HIC1	HIC1	HYPERMETHYLATED IN CANCER 1
23119	HIC2	HIC2	HYPERMETHYLATED IN CANCER 2
3091	HIF1A	HIF1A	HYPOXIA-INDUCIBLE FACTOR 1, ALPHA SUBUNIT (BASIC HELIX-LOOP-HELIX TRANSCRIPTION FACTOR)
55662	HIF1AN	HIF1AN	HYPOXIA-INDUCIBLE FACTOR 1, ALPHA SUBUNIT INHIBITOR
64344	HIF3A	HIF3A	HYPOXIA INDUCIBLE FACTOR 3, ALPHA SUBUNIT
3093	HIP2	HIP2	HUNTINGTIN INTERACTING PROTEIN 2
28996	HIPK2	HIPK2	HOMEODOMAIN INTERACTING PROTEIN KINASE 2
7290	HIRA	HIRA	HIR HISTONE CELL CYCLE REGULATION DEFECTIVE HOMOLOG A (S. CEREVISIAE)
3009	HIST1H1B	HIST1H1B	HISTONE 1, H1B
3006	HIST1H1C	HIST1H1C	HISTONE 1, H1C
3007	HIST1H1D	HIST1H1D	HISTONE 1, H1D
3008	HIST1H1E	HIST1H1E	HISTONE 1, H1E
3010	HIST1H1T	HIST1H1T	HISTONE 1, H1T
8345	HIST1H2BH	HIST1H2BH	HISTONE 1, H2BH
8970	HIST1H2BJ	HIST1H2BJ	HISTONE 1, H2BJ
8341	HIST1H2BN	HIST1H2BN	HISTONE 1, H2BN
3018	HIST1H2BB	HIST2H2BE	HISTONE 1, H2BB
8349	HIST2H2BE	HIST2H2BE	HISTONE 1, H2BB
3096	HIVEP1	HIVEP1	HUMAN IMMUNODEFICIENCY VIRUS TYPE I ENHANCER BINDING PROTEIN 1
3097	HIVEP2	HIVEP2	HUMAN IMMUNODEFICIENCY VIRUS TYPE I ENHANCER BINDING PROTEIN 2
59269	HIVEP3	HIVEP3	HUMAN IMMUNODEFICIENCY VIRUS TYPE I ENHANCER BINDING PROTEIN 3
284459	HKR1	HKR1	GLI-KRUPPEL FAMILY MEMBER HKR1
342945	HKR2	HKR2	GLI-KRUPPEL FAMILY MEMBER HKR2
3104	HKR3	HKR3	GLI-KRUPPEL FAMILY MEMBER HKR3
3131	HLF	HLF	HEPATIC LEUKEMIA FACTOR
3142	HLX1	HLX1	H2.0-LIKE HOMEOBOX 1 (DROSOPHILA)
3110	HLXB9	HLXB9	HOMEOBOX HB9
79618	HMBOX1	HMBOX1	HOMEOBOX CONTAINING 1
10363	HMG20A	HMG20A	HIGH-MOBILITY GROUP 20A
10362	HMG20B	HMG20B	HIGH-MOBILITY GROUP 20B
10042	HMG2L1	HMG2L1	PARTIAL TRANSCRIPT ENCOMPASSING THC211630 GENE
128872	HMG4L	HMG4L	HIGH-MOBILITY GROUP (NONHISTONE CHROMOSOMAL) PROTEIN 4-LIKE
3159	HMGA1	HMGA1	HIGH MOBILITY GROUP AT-HOOK 1
8091	HMGA2	HMGA2	HIGH MOBILITY GROUP AT-HOOK 2
3148	HMGB2	HMGB2	HIGH-MOBILITY GROUP BOX 2
3149	HMGB3	HMGB3	HIGH-MOBILITY GROUP BOX 3
127540	HMGB4	HMGB4	HIGH-MOBILITY GROUP BOX 4
3150	HMGN1	HMGN1	HIGH-MOBILITY GROUP NUCLEOSOME BINDING DOMAIN 1
3151	HMGN2	HMGN2	HIGH-MOBILITY GROUP NUCLEOSOMAL BINDING DOMAIN 2
9324	HMGN3	HMGN3	HIGH MOBILITY GROUP NUCLEOSOMAL BINDING DOMAIN 3
10473	HMGN4	HMGN4	HIGH MOBILITY GROUP NUCLEOSOMAL BINDING DOMAIN 4
3166	HMX1	HMX2	HOMEOBOX (H6 FAMILY) 1
3172	HNF4A	HNF4A	HEPATOCYTE NUCLEAR FACTOR 4, ALPHA
3174	HNF4G	HNF4G	HEPATOCYTE NUCLEAR FACTOR 4, GAMMA
27288	HNRNPG-T	HNRNPG-T	TESTES-SPECIFIC HETEROGENOUS NUCLEAR RIBONUCLEOPROTEIN G-T
3182	HNRPAB	HNRPAB	HETEROGENEOUS NUCLEAR RIBONUCLEOPROTEIN A/B
3184	HNRPD	HNRPD	HETEROGENEOUS NUCLEAR RIBONUCLEOPROTEIN D (AU-RICH ELEMENT RNA BINDING PROTEIN 1, 37KDA)
3190	HNRPK	HNRPK	HETEROGENEOUS NUCLEAR RIBONUCLEOPROTEIN K
10236	HNRPR	HNRPR	HETEROGENEOUS NUCLEAR RIBONUCLEOPROTEIN R
11100	HNRPUL1	HNRPUL1	HETEROGENEOUS NUCLEAR RIBONUCLEOPROTEIN U-LIKE 1
57594	KIAA1443	HOMEZ	homeobox and leucine zipper encoding
84525	HOP	HOP	HOMEODOMAIN-ONLY PROTEIN
3198	HOXA1	HOXA1	HOMEOBOX A1
3206	HOXA10	HOXA10	HOMEOBOX A10
3207	HOXA11	HOXA11	HOMEOBOX A11
3209	HOXA13	HOXA13	HOMEOBOX A13
3199	HOXA2	HOXA2	HOMEOBOX A2
3200	HOXA3	HOXA3	HOMEOBOX A3
3201	HOXA4	HOXA4	HOMEOBOX A4
3202	HOXA5	HOXA5	HOMEOBOX A5
3203	HOXA6	HOXA6	HOMEOBOX A6
3204	HOXA7	HOXA7	HOMEOBOX A7
3205	HOXA9	HOXA9	HOMEOBOX A9
3211	HOXB1	HOXB1	HOMEOBOX B1
10481	HOXB13	HOXB13	HOMEOBOX B13
3212	HOXB2	HOXB2	HOMEOBOX B2
3213	HOXB3	HOXB3	HOMEOBOX B3
3214	HOXB4	HOXB4	HOMEOBOX B4
3215	HOXB5	HOXB5	HOMEOBOX B5
3216	HOXB6	HOXB6	HOMEOBOX B6
3217	HOXB7	HOXB7	HOMEOBOX B7
3218	HOXB8	HOXB8	HOMEOBOX B8
3219	HOXB9	HOXB9	HOMEOBOX B9
3226	HOXC10	HOXC10	HOMEOBOX C10
3227	HOXC11	HOXC11	HOMEOBOX C11
3228	HOXC12	HOXC12	HOMEOBOX C12
3229	HOXC13	HOXC13	HOMEOBOX C13
3221	HOXC4	HOXC4	HOMEOBOX C4
3222	HOXC5	HOXC5	HOMEOBOX C5
3223	HOXC6	HOXC6	HOMEOBOX C6
3224	HOXC8	HOXC8	HOMEOBOX C8
3225	HOXC9	HOXC9	HOMEOBOX C9
3231	HOXD1	HOXD1	HOMEOBOX D1
3236	HOXD10	HOXD10	HOMEOBOX D10
3237	HOXD11	HOXD11	HOMEOBOX D11
3238	HOXD12	HOXD12	HOMEOBOX D12
3239	HOXD13	HOXD13	HOMEOBOX D13
3232	HOXD3	HOXD3	HOMEOBOX D3
3233	HOXD4	HOXD4	HOMEOBOX D4
3234	HOXD8	HOXD8	HOMEOBOX D8
3235	HOXD9	HOXD9	HOMEOBOX D9
55806	HR	HR	HAIRLESS HOMOLOG (MOUSE)
3281	HSBP1	HSBP1	HEAT SHOCK FACTOR BINDING PROTEIN 1
3297	HSF1	HSF1	HEAT SHOCK TRANSCRIPTION FACTOR 1
3298	HSF2	HSF2	HEAT SHOCK TRANSCRIPTION FACTOR 2
11077	HSF2BP	HSF2BP	HEAT SHOCK TRANSCRIPTION FACTOR 2 BINDING PROTEIN
3299	HSF4	HSF4	HEAT SHOCK TRANSCRIPTION FACTOR 4
51402	LW-1	HSFX1	heat shock transcription factor family, X linked 1
86614	HSFY1	HSFY1	HEAT SHOCK TRANSCRIPTION FACTOR, Y-LINKED 1
54737	HSMPP8	HSMPP8	M-PHASE PHOSPHOPROTEIN, MPP8
55552	HSZFP36	HSZFP36	ZFP-36 FOR A ZINC FINGER PROTEIN
10524	HTATIP	HTATIP	HIV-1 TAT INTERACTING PROTEIN, 60KDA
10553	HTATIP2	HTATIP2	HIV-1 TAT INTERACTIVE PROTEIN 2, 30KDA
27336	HTATSF1	HTATSF1	HIV-1 TAT SPECIFIC FACTOR 1
3344	HTLF	HTLF	HUMAN T-CELL LEUKEMIA VIRUS ENHANCER FACTOR
3397	ID1	ID1	INHIBITOR OF DNA BINDING 1, DOMINANT NEGATIVE HELIX-LOOP-HELIX PROTEIN
3398	ID2	ID2	INHIBITOR OF DNA BINDING 2, DOMINANT NEGATIVE HELIX-LOOP-HELIX PROTEIN
3399	ID3	ID3	INHIBITOR OF DNA BINDING 3, DOMINANT NEGATIVE HELIX-LOOP-HELIX PROTEIN
3400	ID4	ID4	INHIBITOR OF DNA BINDING 4, DOMINANT NEGATIVE HELIX-LOOP-HELIX PROTEIN
3428	IFI16	IFI16	INTERFERON, GAMMA-INDUCIBLE PROTEIN 16
3508	IGHMBP2	IGHMBP2	IMMUNOGLOBULIN MU BINDING PROTEIN 2
3551	IKBKB	IKBKB	INHIBITOR OF KAPPA LIGHT POLYPEPTIDE GENE ENHANCER IN B-CELLS, KINASE BETA
8517	IKBKG	IKBKG	INHIBITOR OF KAPPA LIGHT POLYPEPTIDE GENE ENHANCER IN B-CELLS, KINASE GAMMA
3608	ILF2	ILF2	INTERLEUKIN ENHANCER BINDING FACTOR 2, 45KDA
3609	ILF3	ILF3	INTERLEUKIN ENHANCER BINDING FACTOR 3, 90KDA
3621	ING1	ING1	INHIBITOR OF GROWTH FAMILY, MEMBER 1
3622	ING2	ING2	INHIBITOR OF GROWTH FAMILY, MEMBER 2
54556	ING3	ING3	INHIBITOR OF GROWTH FAMILY, MEMBER 3
51147	ING4	ING4	INHIBITOR OF GROWTH FAMILY, MEMBER 4
84289	ING5	ING5	INHIBITOR OF GROWTH FAMILY, MEMBER 5
3642	INSM1	INSM1	INSULINOMA-ASSOCIATED 1
84684	INSM2	INSM2	INSULINOMA-ASSOCIATED 2
3651	IPF1	IPF1	INSULIN PROMOTER FACTOR 1, HOMEODOMAIN TRANSCRIPTION FACTOR
3659	IRF1	IRF1	INTERFERON REGULATORY FACTOR 1
3660	IRF2	IRF2	INTERFERON REGULATORY FACTOR 2
26145	IRF2BP1	IRF2BP1	INTERFERON REGULATORY FACTOR 2 BINDING PROTEIN 1
3661	IRF3	IRF3	INTERFERON REGULATORY FACTOR 3

3662	IRF4	IRF4	INTERFERON REGULATORY FACTOR 4
3663	IRF5	IRF5	INTERFERON REGULATORY FACTOR 5
3664	IRF6	IRF6	INTERFERON REGULATORY FACTOR 6
3665	IRF7	IRF7	INTERFERON REGULATORY FACTOR 7
3394	IRF8	IRF8	INTERFERON REGULATORY FACTOR 8
79192	IRX1	IRX1	IROQUOIS HOMEOBOX PROTEIN 1
153572	IRX2	IRX2	IROQUOIS HOMEOBOX PROTEIN 2
79191	IRX3	IRX3	IROQUOIS HOMEOBOX PROTEIN 3
50805	IRX4	IRX4	IROQUOIS HOMEOBOX PROTEIN 4
10265	IRX5	IRX5	IROQUOIS HOMEOBOX PROTEIN 5
79190	IRX6	IRX6	IROQUOIS HOMEOBOX PROTEIN 6
283078	MKX	IRXL1	IROQUOIS HOMEOBOX PROTEIN-LIKE 1
3670	ISL1	ISL1	ISL1 TRANSCRIPTION FACTOR, LIM/HOMEODOMAIN, (ISLET-1)
64843	ISL2	ISL2	ISL2 TRANSCRIPTION FACTOR, LIM/HOMEODOMAIN, (ISLET-2)
10625	IVNS1ABP	IVNS1ABP	INFLUENZA VIRUS NS1A BINDING PROTEIN
5927	JARID1A	JARID1A	JUMONJI, AT RICH INTERACTIVE DOMAIN 1A (RBBP2-LIKE)
10765	JARID1B	JARID1B	JUMONJI, AT RICH INTERACTIVE DOMAIN 1B (RBP2-LIKE)
3720	JARID2	JARID2	JUMONJI, AT RICH INTERACTIVE DOMAIN 2
221895	tcag7.981	JAZF1	JUXTAPOSED WITH ANOTHER ZINC FINGER GENE 1
122953	JDP2	JDP2	JUN DIMERIZATION PROTEIN 2
221037	JMJD1C	JMJD1C	THYROID HORMONE RECEPTOR INTERACTOR 8
9682	JMJD2A	JMJD2A	JUMONJI DOMAIN CONTAINING 2A
23030	JMJD2B	JMJD2B	JUMONJI DOMAIN CONTAINING 2B
23081	JMJD2C	JMJD2C	JUMONJI DOMAIN CONTAINING 2C
133746	JMY	JMY	JUNCTION-MEDIATING AND REGULATORY PROTEIN
8629	JRK	JRK	JERKY HOMOLOG (MOUSE)
3725	JUN	JUN	V-JUN SARCOMA VIRUS 17 ONCOGENE HOMOLOG (AVIAN)
3726	JUNB	JUNB	JUN B PROTO-ONCOGENE
3727	JUND	JUND	JUN D PROTO-ONCOGENE
30818	KCNIP3	KCNIP3	KV CHANNEL INTERACTING PROTEIN 3, CALSENILIN
10657	KHDRBS1	KHDRBS1	KH DOMAIN CONTAINING, RNA BINDING, SIGNAL TRANSDUCTION ASSOCIATED 1
202559	KHDRBS2	KHDRBS2	KH DOMAIN CONTAINING, RNA BINDING, SIGNAL TRANSDUCTION ASSOCIATED 2
8570	KHSRP	KHSRP	KH-TYPE SPLICING REGULATORY PROTEIN (FUSE BINDING PROTEIN 2)
22993	KIAA0194	KIAA0194	KIAA0194 PROTEIN
10661	KLF1	KLF1	KRUPPEL-LIKE FACTOR 1 (ERYTHROID)
7071	KLF10	KLF10	KRUPPEL-LIKE FACTOR 10
8462	KLF11	KLF11	KRUPPEL-LIKE FACTOR 11
11278	KLF12	KLF12	KRUPPEL-LIKE FACTOR 12
51621	KLF13	KLF13	KRUPPEL-LIKE FACTOR 13
136259	KLF14	KLF14	KRUPPEL-LIKE FACTOR 14
28999	KLF15	KLF15	KRUPPEL-LIKE FACTOR 15
83855	KLF16	KLF16	KRUPPEL-LIKE FACTOR 16
10365	KLF2	KLF2	KRUPPEL-LIKE FACTOR 2 (LUNG)
51274	KLF3	KLF3	KRUPPEL-LIKE FACTOR 3 (BASIC)
9314	KLF4	KLF4	KRUPPEL-LIKE FACTOR 4 (GUT)
688	KLF5	KLF5	KRUPPEL-LIKE FACTOR 5 (INTESTINAL)
1316	KLF6	KLF6	KRUPPEL-LIKE FACTOR 6
8609	KLF7	KLF7	KRUPPEL-LIKE FACTOR 7 (UBIQUITOUS)
11279	KLF8	KLF8	KRUPPEL-LIKE FACTOR 8
687	KLF9	KLF9	KRUPPEL-LIKE FACTOR 9
9735	KNTC1	KNTC1	KINETOCHORE ASSOCIATED 1
84626	KIAA1862	KRBA1	KRAB-A domain containing 1
26013	L3MBTL	L3MBTL	LETHAL (3) MALIGNANT BRAIN TUMOR L(3)MBT PROTEIN (DROSOPHILA) HOMOLOG
83746	L3MBTL2	L3MBTL2	L(3)MBT-LIKE 2 (DROSOPHILA)
84456	L3MBTL3	L3MBTL3	L(3)MBT-LIKE 3 (DROSOPHILA)
91133	L3MBTL4	L3MBTL4	L(3)MBT-LIKE 4 (DROSOPHILA)
29956	LASS2	LASS2	LAG1 LONGEVITY ASSURANCE HOMOLOG 2 (S. CEREVISIAE)
204219	LASS3	LASS3	LAG1 LONGEVITY ASSURANCE HOMOLOG 3 (S. CEREVISIAE)
79603	LASS4	LASS4	LAG1 LONGEVITY ASSURANCE HOMOLOG 4 (S. CEREVISIAE)
91012	LASS5	LASS5	LAG1 LONGEVITY ASSURANCE HOMOLOG 5 (S. CEREVISIAE)
10660	LBX1	LBX1	LADYBIRD HOMEOBOX HOMOLOG 1 (DROSOPHILA)
8861	LDB1	LDB1	LIM DOMAIN BINDING 1
9079	LDB2	LDB2	LIM DOMAIN BINDING 2
51176	LEF1	LEF1	LYMPHOID ENHANCER-BINDING FACTOR 1
3975	LHX1	LHX1	LIM HOMEOBOX 1
9355	LHX2	LHX2	LIM HOMEOBOX 2
8022	LHX3	LHX3	LIM HOMEOBOX 3
89884	LHX4	LHX4	LIM HOMEOBOX 4
64211	LHX5	LHX5	LIM HOMEOBOX 5
26468	LHX6	LHX6	LIM HOMEOBOX 6
56956	LHX9	LHX9	LIM HOMEOBOX 9
8994	LIMD1	LIMD1	LIM DOMAINS CONTAINING 1
79727	LIN28	LIN28	LIN-28 HOMOLOG (C. ELEGANS)
4004	LMO1	LMO1	LIM DOMAIN ONLY 1 (RHOMBOTIN 1)
4005	LMO2	LMO2	LIM DOMAIN ONLY 2 (RHOMBOTIN-LIKE 1)
55885	LMO3	LMO3	LIM DOMAIN ONLY 3 (RHOMBOTIN-LIKE 2)
8543	LMO4	LMO4	LIM DOMAIN ONLY 4
4007	LMO6	LMO6	LIM DOMAIN ONLY 6
4009	LMX1A	LMX1A	LIM HOMEOBOX TRANSCRIPTION FACTOR 1, ALPHA
4010	LMX1B	LMX1B	LIM HOMEOBOX TRANSCRIPTION FACTOR 1, BETA
360030	LOC360030	LOC360030	HOMEOBOX C14
643641	LOC643641	LOC643641	SIMILAR TO ZINC FINGER PROTEIN 74 (HZNF7)
9208	LRRFIP1	LRRFIP1	LEUCINE RICH REPEAT (IN FLII) INTERACTING PROTEIN 1
4066	LYL1	LYL1	LYMPHOBLASTIC LEUKEMIA DERIVED SEQUENCE 1
8216	LZTR1	LZTR1	LEUCINE-ZIPPER-LIKE TRANSCRIPTION REGULATOR 1
11178	LZTS1	LZTS1	LEUCINE ZIPPER, PUTATIVE TUMOR SUPPRESSOR 1
4094	MAF	MAF	V-MAF MUSCULOAPONEUROTIC FIBROSARCOMA ONCOGENE HOMOLOG (AVIAN)
84232	MAF1	MAF1	MAF1 HOMOLOG (S. CEREVISIAE)
389692	MAFA	MAFA	V-MAF MUSCULOAPONEUROTIC FIBROSARCOMA ONCOGENE HOMOLOG A (AVIAN)
9935	MAFB	MAFB	V-MAF MUSCULOAPONEUROTIC FIBROSARCOMA ONCOGENE HOMOLOG B (AVIAN)
23764	MAFF	MAFF	V-MAF MUSCULOAPONEUROTIC FIBROSARCOMA ONCOGENE HOMOLOG F (AVIAN)
4097	MAFG	MAFG	V-MAF MUSCULOAPONEUROTIC FIBROSARCOMA ONCOGENE HOMOLOG G (AVIAN)
7975	MAFK	MAFK	V-MAF MUSCULOAPONEUROTIC FIBROSARCOMA ONCOGENE HOMOLOG K (AVIAN)
9794	MAML1	MAML1	MASTERMIND-LIKE 1 (DROSOPHILA)
55534	MAML3	MAML3	MASTERMIND-LIKE 3 (DROSOPHILA)
9782	MATR3	MATR3	MATRIN 3
4149	MAX	MAX	MYC ASSOCIATED FACTOR X
4150	MAZ	MAZ	MYC-ASSOCIATED ZINC FINGER PROTEIN (PURINE-BINDING TRANSCRIPTION FACTOR)
4152	MBD1	MBD1	METHYL-CPG BINDING DOMAIN PROTEIN 1
8932	MBD2	MBD2	METHYL-CPG BINDING DOMAIN PROTEIN 2
53615	MBD3	MBD3	METHYL-CPG BINDING DOMAIN PROTEIN 3
85509	MBD3L1	MBD3L1	METHYL-CPG BINDING DOMAIN PROTEIN 3-LIKE 1
4171	MCM2	MCM2	MCM2 MINICHROMOSOME MAINTENANCE DEFICIENT 2, MITOTIN (S. CEREVISIAE)
4172	MCM3	MCM3	MCM3 MINICHROMOSOME MAINTENANCE DEFICIENT 3 (S. CEREVISIAE)
4173	MCM4	MCM4	MCM4 MINICHROMOSOME MAINTENANCE DEFICIENT 4 (S. CEREVISIAE)
4174	MCM5	MCM5	MCM5 MINICHROMOSOME MAINTENANCE DEFICIENT 5, CELL DIVISION CYCLE 46 (S. CEREVISIAE)
4175	MCM6	MCM6	MCM6 MINICHROMOSOME MAINTENANCE DEFICIENT 6 (MIS5 HOMOLOG, S. POMBE) (S. CEREVISIAE)
4176	MCM7	MCM7	MCM7 MINICHROMOSOME MAINTENANCE DEFICIENT 7 (S. CEREVISIAE)
84515	MCM8	MCM8	MCM8 MINICHROMOSOME MAINTENANCE DEFICIENT 8 (S. CEREVISIAE)
254394	MCMDC1	MCMDC1	MINICHROMOSOME MAINTENANCE DEFICIENT DOMAIN CONTAINING 1
4194	MDM4	MDM4	MDM4, TRANSFORMED 3T3 CELL DOUBLE MINUTE 4, P53 BINDING PROTEIN (MOUSE)
4204	MECP2	MECP2	METHYL CPG BINDING PROTEIN 2 (RETT SYNDROME)
9968	MED12	MED12	MEDIATOR OF RNA POLYMERASE II TRANSCRIPTION, SUBUNIT 12 HOMOLOG (YEAST)
51003	MED31	MED31	MEDIATOR OF RNA POLYMERASE II TRANSCRIPTION, SUBUNIT 31 HOMOLOG (YEAST)
29079	MED4	MED4	MEDIATOR OF RNA POLYMERASE II TRANSCRIPTION, SUBUNIT 4 HOMOLOG (YEAST)
10001	MED6	MED6	MEDIATOR OF RNA POLYMERASE II TRANSCRIPTION, SUBUNIT 6 HOMOLOG (YEAST)
112950	MED8	MED8	MEDIATOR OF RNA POLYMERASE II TRANSCRIPTION, SUBUNIT 8 HOMOLOG (YEAST)
4205	MEF2A	MEF2A	MADS BOX TRANSCRIPTION ENHANCER FACTOR 2, POLYPEPTIDE A (MYOCYTE ENHANCER FACTOR 2A)
4207	MEF2B	MEF2B	MADS BOX TRANSCRIPTION ENHANCER FACTOR 2, POLYPEPTIDE B (MYOCYTE ENHANCER FACTOR 2B)
4208	MEF2C	MEF2C	MADS BOX TRANSCRIPTION ENHANCER FACTOR 2, POLYPEPTIDE C (MYOCYTE ENHANCER FACTOR 2C)
4209	MEF2D	MEF2D	MADS BOX TRANSCRIPTION ENHANCER FACTOR 2, POLYPEPTIDE D (MYOCYTE ENHANCER FACTOR 2D)
4211	MEIS1	MEIS1	MEIS1, MYELOID ECOTROPIC VIRAL INTEGRATION SITE 1 HOMOLOG (MOUSE)
4212	MEIS2	MEIS2	MEIS1, MYELOID ECOTROPIC VIRAL INTEGRATION SITE 1 HOMOLOG 2 (MOUSE)
56917	MEIS3	MEIS3	MEIS1, MYELOID ECOTROPIC VIRAL INTEGRATION SITE 1 HOMOLOG 3 (MOUSE)
4213	MEIS3P1	MEIS3P1	MEIS1 HOMOLOG 3 (MOUSE) PSEUDOGENE 1
4221	MEN1	MEN1	MULTIPLE ENDOCRINE NEOPLASIA I
4222	MEOX1	MEOX1	MESENCHYME HOMEOBOX 1
4223	MEOX2	MEOX2	MESENCHYME HOMEOBOX 2
170082	MGC17403	MGC17403	TFIIS central domain-containing protein 1

65988	ZNF747	MGC2474	ZINC FINGER PROTEIN 747
4286	MITF	MITF	MICROPHTHALMIA-ASSOCIATED TRANSCRIPTION FACTOR
83881	MIXL1	MIXL1	MIX1 HOMEOBOX-LIKE 1 (XENOPUS LAEVIS)
25988	MIZF	MIZF	MBD2-INTERACTING ZINC FINGER
84365	MKI67IP	MKI67IP	MKI67 (FHA DOMAIN) INTERACTING NUCLEOLAR PHOSPHOPROTEIN
57591	MKL1	MKL1	MEGAKARYOBLASTIC LEUKEMIA (TRANSLOCATION) 1
57496	MKL2	MKL2	MKL/MYOCARDIN-LIKE 2
4297	MLL	MLL	MYELOID/LYMPHOID OR MIXED-LINEAGE LEUKEMIA (TRITHORAX HOMOLOG, DROSOPHILA)
8085	MLL2	MLL2	MYELOID/LYMPHOID OR MIXED-LINEAGE LEUKEMIA 2
58508	MLL3	MLL3	MYELOID/LYMPHOID OR MIXED-LINEAGE LEUKEMIA 3
9757	MLL4	MLL4	MYELOID/LYMPHOID OR MIXED-LINEAGE LEUKEMIA 4
55904	MLL5	MLL5	MYELOID/LYMPHOID OR MIXED-LINEAGE LEUKEMIA 5
4298	MLLT1	MLLT1	MYELOID/LYMPHOID OR MIXED-LINEAGE LEUKEMIA (TRITHORAX HOMOLOG, DROSOPHILA); TRANSLOCATED TO, 1
8028	MLLT10	MLLT10	MYELOID/LYMPHOID OR MIXED-LINEAGE LEUKEMIA (TRITHORAX HOMOLOG, DROSOPHILA); TRANSLOCATED TO, 10
4300	MLLT3	MLLT3	MYELOID/LYMPHOID OR MIXED-LINEAGE LEUKEMIA (TRITHORAX HOMOLOG, DROSOPHILA); TRANSLOCATED TO, 3
4302	MLLT6	MLLT6	MYELOID/LYMPHOID OR MIXED-LINEAGE LEUKEMIA (TRITHORAX HOMOLOG, DROSOPHILA); TRANSLOCATED TO, 6
4303	MLLT7	MLLT7	MYELOID/LYMPHOID OR MIXED-LINEAGE LEUKEMIA (TRITHORAX HOMOLOG, DROSOPHILA); TRANSLOCATED TO, 7
254251	MLR1	MLR1	TRANSCRIPTION FACTOR MLR1
84458	MLR2	MLR2	LIGAND-DEPENDENT COREPRESSOR
6945	MLX	MLX	MAX-LIKE PROTEIN X
22877	MLXIP	MLXIP	MLX INTERACTING PROTEIN
51085	MLXIPL	MLXIPL	MLX INTERACTING PROTEIN-LIKE
64210	MMS19L	MMS19L	MMS19-LIKE (MET18 HOMOLOG, S. CEREVISIAE)
4332	MNDA	MNDA	MYELOID CELL NUCLEAR DIFFERENTIATION ANTIGEN
4335	MNT	MNT	MAX BINDING PROTEIN
10934	MORF4	MORF4	MORTALITY FACTOR 4
9242	MSC	MSC	MUSCULIN (ACTIVATED B-CELL FACTOR-1)
10943	MSL3L1	MSL3L1	MALE-SPECIFIC LETHAL 3-LIKE 1 (DROSOPHILA)
4487	MSX1	MSX1	MSH HOMEOBOX HOMOLOG 1 (DROSOPHILA)
4488	MSX2	MSX2	MSH HOMEOBOX HOMOLOG 2 (DROSOPHILA)
9112	MTA1	MTA1	METASTASIS ASSOCIATED 1
9219	MTA2	MTA2	METASTASIS ASSOCIATED 1 FAMILY, MEMBER 2
57504	MTA3	MTA3	METASTASIS ASSOCIATED 1 FAMILY, MEMBER 3
4520	MTF1	MTF1	METAL-REGULATORY TRANSCRIPTION FACTOR 1
22823	MTF2	MTF2	METAL RESPONSE ELEMENT BINDING TRANSCRIPTION FACTOR 2
4084	MXD1	MXD1	MAX DIMERIZATION PROTEIN 1
83463	MXD3	MXD3	MAX DIMERIZATION PROTEIN 3
10608	MXD4	MXD4	MAX DIMERIZATION PROTEIN 4
4601	MXI1	MXI1	MAX INTERACTOR 1
4602	MYB	MYB	V-MYB MYELOBLASTOSIS VIRAL ONCOGENE HOMOLOG (AVIAN)
10514	MYBBP1A	MYBBP1A	MYB BINDING PROTEIN (P160) 1A
4603	MYBL1	MYBL1	V-MYB MYELOBLASTOSIS VIRAL ONCOGENE HOMOLOG (AVIAN)-LIKE 1
4605	MYBL2	MYBL2	V-MYB MYELOBLASTOSIS VIRAL ONCOGENE HOMOLOG (AVIAN)-LIKE 2
4609	MYC	MYC	V-MYC MYELOCYTOMATOSIS VIRAL ONCOGENE HOMOLOG (AVIAN)
26292	MYCBP	MYCBP	C-MYC BINDING PROTEIN
4610	MYCL1	MYCL1	V-MYC MYELOCYTOMATOSIS VIRAL ONCOGENE HOMOLOG 1, LUNG CARCINOMA DERIVED (AVIAN)
4613	MYCN	MYCN	V-MYC MYELOCYTOMATOSIS VIRAL RELATED ONCOGENE, NEUROBLASTOMA DERIVED (AVIAN)
50804	MYEF2	MYEF2	MYELIN EXPRESSION FACTOR 2
4617	MYF5	MYF5	MYOGENIC FACTOR 5
4618	MYF6	MYF6	MYOGENIC FACTOR 6 (HERCULIN)
55892	MYNN	MYNN	MYONEURIN
93649	MYOCD	MYOCD	MYOCARDIN
4654	MYOD1	MYOD1	MYOGENIC DIFFERENTIATION 1
4656	MYOG	MYOG	MYOGENIN (MYOGENIC FACTOR 4)
11143	MYST2	MYST2	MYST HISTONE ACETYLTRANSFERASE 2
7994	MYST3	MYST3	MYST HISTONE ACETYLTRANSFERASE (MONOCYTIC LEUKEMIA) 3
23522	MYST4	MYST4	MYST HISTONE ACETYLTRANSFERASE (MONOCYTIC LEUKEMIA) 4
23040	MYT1L	MYT1L	MYELIN TRANSCRIPTION FACTOR 1-LIKE
4664	NAB1	NAB1	NGFI-A BINDING PROTEIN 1 (EGR1 BINDING PROTEIN 1)
4665	NAB2	NAB2	NGFI-A BINDING PROTEIN 2 (EGR1 BINDING PROTEIN 2)
79923	NANOG	NANOG	NANOG HOMEOBOX
4674	NAP1L2	NAP1L2	NUCLEOSOME ASSEMBLY PROTEIN 1-LIKE 2
4675	NAP1L3	NAP1L3	NUCLEOSOME ASSEMBLY PROTEIN 1-LIKE 3
4676	NAP1L4	NAP1L4	NUCLEOSOME ASSEMBLY PROTEIN 1-LIKE 4
80155	NARG1	NARG1	NMDA RECEPTOR REGULATED 1
8648	NCOA1	NCOA1	NUCLEAR RECEPTOR COACTIVATOR 1
10499	NCOA2	NCOA2	NUCLEAR RECEPTOR COACTIVATOR 2
8202	NCOA3	NCOA3	NUCLEAR RECEPTOR COACTIVATOR 3
8031	NCOA4	NCOA4	NUCLEAR RECEPTOR COACTIVATOR 4
57727	NCOA5	NCOA5	NUCLEAR RECEPTOR COACTIVATOR 5
23054	NCOA6	NCOA6	NUCLEAR RECEPTOR COACTIVATOR 6
9611	NCOR1	NCOR1	NUCLEAR RECEPTOR CO-REPRESSOR 1
9612	NCOR2	NCOR2	NUCLEAR RECEPTOR CO-REPRESSOR 2
4692	NDN	NDN	NECDIN HOMOLOG (MOUSE)
56160	NDNL2	NDNL2	NECDIN-LIKE 2
252969	NEIL2	NEIL2	HYPOTHETICAL PROTEIN MGC2832
4760	NEUROD1	NEUROD1	NEUROGENIC DIFFERENTIATION 1
4761	NEUROD2	NEUROD2	NEUROGENIC DIFFERENTIATION 2
58158	NEUROD4	NEUROD4	NEUROGENIC DIFFERENTIATION 4
63974	NEUROD6	NEUROD6	NEUROGENIC DIFFERENTIATION 6
4762	NEUROG1	NEUROG1	NEUROGENIN 1
63973	NEUROG2	NEUROG2	NEUROGENIN 2
50674	NEUROG3	NEUROG3	NEUROGENIN 3
10725	NFAT5	NFAT5	NUCLEAR FACTOR OF ACTIVATED T-CELLS 5, TONICITY-RESPONSIVE
4772	NFATC1	NFATC1	NUCLEAR FACTOR OF ACTIVATED T-CELLS, CYTOPLASMIC, CALCINEURIN-DEPENDENT 1
4773	NFATC2	NFATC2	NUCLEAR FACTOR OF ACTIVATED T-CELLS, CYTOPLASMIC, CALCINEURIN-DEPENDENT 2
4775	NFATC3	NFATC3	NUCLEAR FACTOR OF ACTIVATED T-CELLS, CYTOPLASMIC, CALCINEURIN-DEPENDENT 3
4776	NFATC4	NFATC4	NUCLEAR FACTOR OF ACTIVATED T-CELLS, CYTOPLASMIC, CALCINEURIN-DEPENDENT 4
4778	NFE2	NFE2	NUCLEAR FACTOR (ERYTHROID-DERIVED 2), 45KDA
4779	NFE2L1	NFE2L1	NUCLEAR FACTOR (ERYTHROID-DERIVED 2)-LIKE 1
4780	NFE2L2	NFE2L2	NUCLEAR FACTOR (ERYTHROID-DERIVED 2)-LIKE 2
9603	NFE2L3	NFE2L3	NUCLEAR FACTOR (ERYTHROID-DERIVED 2)-LIKE 3
4774	NFIA	NFIA	NUCLEAR FACTOR I/A
4781	NFIB	NFIB	NUCLEAR FACTOR I/B
4782	NFIC	NFIC	NUCLEAR FACTOR I/C (CCAAT-BINDING TRANSCRIPTION FACTOR)
4783	NFIL3	NFIL3	NUCLEAR FACTOR, INTERLEUKIN 3 REGULATED
4784	NFIX	NFIX	NUCLEAR FACTOR I/X (CCAAT-BINDING TRANSCRIPTION FACTOR)
4790	NFKB1	NFKB1	NUCLEAR FACTOR OF KAPPA LIGHT POLYPEPTIDE GENE ENHANCER IN B-CELLS 1 (P105)
4791	NFKB2	NFKB2	NUCLEAR FACTOR OF KAPPA LIGHT POLYPEPTIDE GENE ENHANCER IN B-CELLS 2 (P49/P100)
4792	NFKBIA	NFKBIA	NUCLEAR FACTOR OF KAPPA LIGHT POLYPEPTIDE GENE ENHANCER IN B-CELLS INHIBITOR, ALPHA
4793	NFKBIB	NFKBIB	NUCLEAR FACTOR OF KAPPA LIGHT POLYPEPTIDE GENE ENHANCER IN B-CELLS INHIBITOR, BETA
4795	NFKBIL1	NFKBIL1	NUCLEAR FACTOR OF KAPPA LIGHT POLYPEPTIDE GENE ENHANCER IN B-CELLS INHIBITOR-LIKE 1
4796	NFKBIL2	NFKBIL2	NUCLEAR FACTOR OF KAPPA LIGHT POLYPEPTIDE GENE ENHANCER IN B-CELLS INHIBITOR-LIKE 2
64332	NFKBIZ	NFKBIZ	NUCLEAR FACTOR OF KAPPA LIGHT POLYPEPTIDE GENE ENHANCER IN B-CELLS INHIBITOR, ZETA
4798	NFRKB	NFRKB	NUCLEAR FACTOR RELATED TO KAPPAB BINDING PROTEIN
4799	NFX1	NFX1	NUCLEAR TRANSCRIPTION FACTOR, X-BOX BINDING 1
152518	NFXL1	NFXL1	NUCLEAR TRANSCRIPTION FACTOR, X-BOX BINDING-LIKE 1
4800	NFYA	NFYA	NUCLEAR TRANSCRIPTION FACTOR Y, ALPHA
4801	NFYB	NFYB	NUCLEAR TRANSCRIPTION FACTOR Y, BETA
4802	NFYC	NFYC	NUCLEAR TRANSCRIPTION FACTOR Y, GAMMA
4807	NHLH1	NHLH1	NESCIENT HELIX LOOP HELIX 1
4808	NHLH2	NHLH2	NESCIENT HELIX LOOP HELIX 2
85409	NKD2	NKD2	NAKED CUTICLE HOMOLOG 2 (DROSOPHILA)
55922	NKRF	NKRF	NFKB repressing factor
4821	NKX2-2	NKX2-2	NK2 TRANSCRIPTION FACTOR RELATED, LOCUS 2 (DROSOPHILA)
159296	NKX2-3	NKX2-3	NK2 TRANSCRIPTION FACTOR HOMOLOG C (DROSOPHILA)
1482	NKX2-5	NKX2-5	NK2 TRANSCRIPTION FACTOR RELATED, LOCUS 5 (DROSOPHILA)
26257	NKX2-8	NKX2-8	NK2 TRANSCRIPTION FACTOR RELATED, LOCUS 8 (DROSOPHILA)
4824	NKX3-1	NKX3-1	NK3 TRANSCRIPTION FACTOR RELATED, LOCUS 1 (DROSOPHILA)
4825	NKX6-1	NKX6-1	NK6 TRANSCRIPTION FACTOR RELATED, LOCUS 1 (DROSOPHILA)
84504	NKX6-2	NKX6-2	NK6 TRANSCRIPTION FACTOR RELATED, LOCUS 2 (DROSOPHILA)
157848	FLJ25169	NKX6-3	NK6 homeobox 3
4831	NME2	NME2	NON-METASTATIC CELLS 2, PROTEIN (NM23B) EXPRESSED IN
9111	NMI	NMI	N-MYC (AND STAT) INTERACTOR
8725	C19orf2	NNX3	CHROMOSOME 19 OPEN READING FRAME 2
4841	NONO	NONO	NON-POU DOMAIN CONTAINING, OCTAMER-BINDING
4853	NOTCH2	NOTCH2	NOTCH HOMOLOG 2 (DROSOPHILA)
4854	NOTCH3	NOTCH3	NOTCH HOMOLOG 3 (DROSOPHILA)
4855	NOTCH4	NOTCH4	NOTCH HOMOLOG 4 (DROSOPHILA)

4861	NPAS1	NPAS1	NEURONAL PAS DOMAIN PROTEIN 1
4862	NPAS2	NPAS2	NEURONAL PAS DOMAIN PROTEIN 2
64067	NPAS3	NPAS3	NEURONAL PAS DOMAIN PROTEIN 3
4869	NPM1	NPM1	NUCLEOPHOSMIN (NUCLEOLAR PHOSPHOPROTEIN B23, NUMATRIN)
10361	NPM2	NPM2	NUCLEOPHOSMIN/NUCLEOPLASMIN, 2
190	NR0B1	NR0B1	NUCLEAR RECEPTOR SUBFAMILY 0, GROUP B, MEMBER 1
8431	NR0B2	NR0B2	NUCLEAR RECEPTOR SUBFAMILY 0, GROUP B, MEMBER 2
9572	NR1D1	NR1D1	NUCLEAR RECEPTOR SUBFAMILY 1, GROUP D, MEMBER 1
9975	NR1D2	NR1D2	NUCLEAR RECEPTOR SUBFAMILY 1, GROUP D, MEMBER 2
7376	NR1H2	NR1H2	NUCLEAR RECEPTOR SUBFAMILY 1, GROUP H, MEMBER 2
10062	NR1H3	NR1H3	NUCLEAR RECEPTOR SUBFAMILY 1, GROUP H, MEMBER 3
9971	NR1H4	NR1H4	NUCLEAR RECEPTOR SUBFAMILY 1, GROUP H, MEMBER 4
8856	NR1I2	NR1I2	NUCLEAR RECEPTOR SUBFAMILY 1, GROUP I, MEMBER 2
9970	NR1I3	NR1I3	NUCLEAR RECEPTOR SUBFAMILY 1, GROUP I, MEMBER 3
7181	NR2C1	NR2C1	NUCLEAR RECEPTOR SUBFAMILY 2, GROUP C, MEMBER 1
7182	NR2C2	NR2C2	NUCLEAR RECEPTOR SUBFAMILY 2, GROUP C, MEMBER 2
7101	NR2E1	NR2E1	NUCLEAR RECEPTOR SUBFAMILY 2, GROUP E, MEMBER 1
10002	NR2E3	NR2E3	NUCLEAR RECEPTOR SUBFAMILY 2, GROUP E, MEMBER 3
7025	NR2F1	NR2F1	NUCLEAR RECEPTOR SUBFAMILY 2, GROUP F, MEMBER 1
7026	NR2F2	NR2F2	NUCLEAR RECEPTOR SUBFAMILY 2, GROUP F, MEMBER 2
2063	NR2F6	NR2F6	NUCLEAR RECEPTOR SUBFAMILY 2, GROUP F, MEMBER 6
2908	NR3C1	NR3C1	NUCLEAR RECEPTOR SUBFAMILY 3, GROUP C, MEMBER 1 (GLUCOCORTICOID RECEPTOR)
4306	NR3C2	NR3C2	NUCLEAR RECEPTOR SUBFAMILY 3, GROUP C, MEMBER 2
3164	NR4A1	NR4A1	NUCLEAR RECEPTOR SUBFAMILY 4, GROUP A, MEMBER 1
4929	NR4A2	NR4A2	NUCLEAR RECEPTOR SUBFAMILY 4, GROUP A, MEMBER 2
8013	NR4A3	NR4A3	NUCLEAR RECEPTOR SUBFAMILY 4, GROUP A, MEMBER 3
2516	NR5A1	NR5A1	NUCLEAR RECEPTOR SUBFAMILY 5, GROUP A, MEMBER 1
2494	NR5A2	NR5A2	NUCLEAR RECEPTOR SUBFAMILY 5, GROUP A, MEMBER 2
29982	NRBF2	NRBF2	NUCLEAR RECEPTOR BINDING FACTOR 2
4899	NRF1	NRF1	NUCLEAR RESPIRATORY FACTOR 1
8204	NRIP1	NRIP1	NUCLEAR RECEPTOR INTERACTING PROTEIN 1
4901	NRL	NRL	NEURAL RETINA LEUCINE ZIPPER
79366	NSBP1	NSBP1	NUCLEOSOMAL BINDING PROTEIN 1
64324	NSD1	NSD1	NUCLEAR RECEPTOR BINDING SET DOMAIN PROTEIN 1
116448	OLIG1	OLIG1	OLIGODENDROCYTE TRANSCRIPTION FACTOR 1
10215	OLIG2	OLIG2	OLIGODENDROCYTE LINEAGE TRANSCRIPTION FACTOR 2
167826	OLIG3	OLIG3	OLIGODENDROCYTE TRANSCRIPTION FACTOR 3
3175	ONECUT1	ONECUT1	ONE CUT DOMAIN, FAMILY MEMBER 1
9480	ONECUT2	ONECUT2	ONE CUT DOMAIN, FAMILY MEMBER 2
4999	ORC2L	ORC2L	ORIGIN RECOGNITION COMPLEX, SUBUNIT 2-LIKE (YEAST)
158800	OTEX	OTEX	PAIRED-LIKE HOMEOBOX PROTEIN OTEX
5013	OTX1	OTX1	ORTHODENTICLE HOMOLOG 1 (DROSOPHILA)
5015	OTX2	OTX2	ORTHODENTICLE HOMOLOG 2 (DROSOPHILA)
1406	CRX	OTX3	CONE-ROD HOMEOBOX
5017	OVOL1	OVOL1	OVO-LIKE 1(DROSOPHILA)
58495	OVOL2	OVOL2	OVO-LIKE 2 (DROSOPHILA)
26471	P8	P8	P8 PROTEIN (CANDIDATE OF METASTASIS 1)
5036	PA2G4	PA2G4	PROLIFERATION-ASSOCIATED 2G4, 38KDA
10914	PAPOLA	PAPOLA	POLY(A) POLYMERASE ALPHA
56903	PAPOLB	PAPOLB	POLY(A) POLYMERASE BETA (TESTIS SPECIFIC)
64895	PAPOLG	PAPOLG	POLY(A) POLYMERASE GAMMA
5073	PARN	PARN	POLY(A)-SPECIFIC RIBONUCLEASE (DEADENYLATION NUCLEASE)
142	PARP1	PARP1	POLY (ADP-RIBOSE) POLYMERASE FAMILY, MEMBER 1
5074	PAWR	PAWR	PRKC, APOPTOSIS, WT1, REGULATOR
5075	PAX1	PAX1	PAIRED BOX GENE 1
5076	PAX2	PAX2	PAIRED BOX GENE 2
5077	PAX3	PAX3	PAIRED BOX GENE 3 (WAARDENBURG SYNDROME 1)
5078	PAX4	PAX4	PAIRED BOX GENE 4
5079	PAX5	PAX5	PAIRED BOX GENE 5 (B-CELL LINEAGE SPECIFIC ACTIVATOR)
5080	PAX6	PAX6	PAIRED BOX GENE 6 (ANIRIDIA, KERATITIS)
5081	PAX7	PAX7	PAIRED BOX GENE 7
7849	PAX8	PAX8	PAIRED BOX GENE 8
5083	PAX9	PAX9	PAIRED BOX GENE 9
55193	PB1	PB1	POLYBROMO 1
5087	PBX1	PBX1	PRE-B-CELL LEUKEMIA TRANSCRIPTION FACTOR 1
5089	PBX2	PBX2	PRE-B-CELL LEUKEMIA TRANSCRIPTION FACTOR 2
5090	PBX3	PBX3	PRE-B-CELL LEUKEMIA TRANSCRIPTION FACTOR 3
80714	PBX4	PBX4	PRE-B-CELL LEUKEMIA TRANSCRIPTION FACTOR 4
57326	PBXIP1	PBXIP1	PRE-B-CELL LEUKEMIA TRANSCRIPTION FACTOR INTERACTING PROTEIN 1
8850	PCAF	PCAF	P300/CBP-ASSOCIATED FACTOR
5092	PCBD1	PCBD1	PTERIN-4 ALPHA-CARBINOLAMINE DEHYDRATASE/DIMERIZATION COFACTOR OF HEPATOCYTE NUCLEAR FACTOR 1 ALPHA (TCF1)
84105	PCBD2	PCBD2	PTERIN-4 ALPHA-CARBINOLAMINE DEHYDRATASE/DIMERIZATION COFACTOR OF HEPATOCYTE NUCLEAR FACTOR 1 ALPHA (TCF1) 2
7703	PCGF2	PCGF2	POLYCOMB GROUP RING FINGER 2
648	PCGF4	PCGF4	POLYCOMB GROUP RING FINGER 4
84108	PCGF6	PCGF6	POLYCOMB GROUP RING FINGER 6
4661	MYT1	PCMTD2	MYELIN TRANSCRIPTION FACTOR 1
51586	PCQAP	PCQAP	TRINUCLEOTIDE REPEAT CONTAINING 7
22984	PDCD11	PDCD11	PROGRAMMED CELL DEATH 11
9124	PDLIM1	PDLIM1	PDZ AND LIM DOMAIN 1 (ELFIN)
8572	PDLIM4	PDLIM4	PDZ AND LIM DOMAIN 4
5178	PEG3	PEG3	PATERNALLY EXPRESSED 3
84528	PEPP-2	PEPP-2	PEPP SUBFAMILY GENE 2
5187	PER1	PER1	PERIOD HOMOLOG 1 (DROSOPHILA)
8864	PER2	PER2	PERIOD HOMOLOG 2 (DROSOPHILA)
8863	PER3	PER3	PERIOD HOMOLOG 3 (DROSOPHILA)
84547	PGBD1	PGBD1	PIGGYBAC TRANSPOSABLE ELEMENT DERIVED 1
2074	ERCC6	PGBD3????	EXCISION REPAIR CROSS-COMPLEMENTING RODENT REPAIR DEFICIENCY, COMPLEMENTATION GROUP 6
5241	PGR	PGR	PROGESTERONE RECEPTOR
11331	PHB2	PHB2	PROHIBITIN 2
5252	PHF1	PHF1	PHD FINGER PROTEIN 1
55274	PHF10	PHF10	PHD FINGER PROTEIN 10
57649	PHF12	PHF12	PHD FINGER PROTEIN 12
148479	PHF13	PHF13	PHD FINGER PROTEIN 13
9678	PHF14	PHF14	PHD FINGER PROTEIN 14
23338	PHF15	PHF15	PHD FINGER PROTEIN 15
9767	PHF16	PHF16	PHD FINGER PROTEIN 16
79960	PHF17	PHF17	PHD FINGER PROTEIN 17
26147	PHF19	PHF19	PHD FINGER PROTEIN 19
5253	PHF2	PHF2	PHD FINGER PROTEIN 2
51230	PHF20	PHF20	PHD FINGER PROTEIN 20
51317	PHF21A	PHF21A	PHD FINGER PROTEIN 21A
112885	PHF21B	PHF21B	PHD FINGER PROTEIN 21B
57117	INTS12	PHF22	PHD FINGER PROTEIN 22
79142	PHF23	PHF23	PHD FINGER PROTEIN 23
23469	PHF3	PHF3	PHD FINGER PROTEIN 3
84844	PHF5A	PHF5A	PHD FINGER PROTEIN 5A
84295	PHF6	PHF6	PHD FINGER PROTEIN 6
51533	PHF7	PHF7	PHD FINGER PROTEIN 7
23133	PHF8	PHF8	PHD FINGER PROTEIN 8
401	PHOX2A	PHOX2A	PAIRED-LIKE (ARISTALESS) HOMEOBOX 2A
8929	PHOX2B	PHOX2B	PAIRED-LIKE HOMEOBOX 2B
10745	PHTF1	PHTF1	PUTATIVE HOMEODOMAIN TRANSCRIPTION FACTOR 1
8554	PIAS1	PIAS1	PROTEIN INHIBITOR OF ACTIVATED STAT, 1
9063	PIAS2	PIAS2	PROTEIN INHIBITOR OF ACTIVATED STAT, 2
10401	PIAS3	PIAS3	PROTEIN INHIBITOR OF ACTIVATED STAT, 3
51588	PIAS4	PIAS4	PROTEIN INHIBITOR OF ACTIVATED STAT, 4
8544	PIR	PIR	PIRIN (IRON-BINDING NUCLEAR PROTEIN)
5307	PITX1	PITX1	PAIRED-LIKE HOMEODOMAIN TRANSCRIPTION FACTOR 1
5308	PITX2	PITX2	PAIRED-LIKE HOMEODOMAIN TRANSCRIPTION FACTOR 2
5309	PITX3	PITX3	PAIRED-LIKE HOMEODOMAIN TRANSCRIPTION FACTOR 3
5316	PKNOX1	PKNOX1	PBX/KNOTTED 1 HOMEOBOX 1
63876	PKNOX2	PKNOX2	PBX/KNOTTED 1 HOMEOBOX 2
5324	PLAG1	PLAG1	PLEIOMORPHIC ADENOMA GENE 1
5325	PLAGL1	PLAGL1	PLEIOMORPHIC ADENOMA GENE-LIKE 1
5326	PLAGL2	PLAGL2	PLEIOMORPHIC ADENOMA GENE-LIKE 2
5356	PLRG1	PLRG1	PLEIOTROPIC REGULATOR 1 (PRL1 HOMOLOG, ARABIDOPSIS)
5371	PML	PML	PROMYELOCYTIC LEUKEMIA
5378	PMS1	PMS1	PMS1 POSTMEIOTIC SEGREGATION INCREASED 1 (S. CEREVISIAE)
5387	PMS2L3	PMS2L3	POSTMEIOTIC SEGREGATION INCREASED 2-LIKE 3

5411	PNN	PNN	PININ, DESMOSOME ASSOCIATED PROTEIN
55629	PNRC2	PNRC2	PROLINE-RICH NUCLEAR RECEPTOR COACTIVATOR 2
57645	POGK	POGK	POGO TRANSPOSABLE ELEMENT WITH KRAB DOMAIN
25885	POLR1A	POLR1A	POLYMERASE (RNA) I POLYPEPTIDE A, 194KDA
84172	POLR1B	POLR1B	POLYMERASE (RNA) I POLYPEPTIDE B, 194KDA
9533	POLR1C	POLR1C	POLYMERASE (RNA) I POLYPEPTIDE C, 30KDA
51082	POLR1D	POLR1D	POLYMERASE (RNA) I POLYPEPTIDE D, 16KDA
64425	POLR1E	POLR1E	POLYMERASE (RNA) I ASSOCIATED FACTOR 1
5430	POLR2A	POLR2A	POLYMERASE (RNA) II (DNA DIRECTED) POLYPEPTIDE A, 220KDA
5431	POLR2B	POLR2B	POLYMERASE (RNA) II (DNA DIRECTED) POLYPEPTIDE B, 140KDA
5432	POLR2C	POLR2C	POLYMERASE (RNA) II (DNA DIRECTED) POLYPEPTIDE C, 33KDA
5433	POLR2D	POLR2D	POLYMERASE (RNA) II (DNA DIRECTED) POLYPEPTIDE D
5434	POLR2E	POLR2E	POLYMERASE (RNA) II (DNA DIRECTED) POLYPEPTIDE E, 25KDA
5435	POLR2F	POLR2F	POLYMERASE (RNA) II (DNA DIRECTED) POLYPEPTIDE F
5436	POLR2G	POLR2G	POLYMERASE (RNA) II (DNA DIRECTED) POLYPEPTIDE G
5437	POLR2H	POLR2H	POLYMERASE (RNA) II (DNA DIRECTED) POLYPEPTIDE H
5438	POLR2I	POLR2I	POLYMERASE (RNA) II (DNA DIRECTED) POLYPEPTIDE I, 14.5KDA
5439	POLR2J	POLR2J	POLYMERASE (RNA) II (DNA DIRECTED) POLYPEPTIDE J, 13.3KDA
246721	POLR2J2	POLR2J3	DNA DIRECTED RNA POLYMERASE II POLYPEPTIDE J-RELATED GENE
5440	POLR2K	POLR2K	POLYMERASE (RNA) II (DNA DIRECTED) POLYPEPTIDE K, 7.0KDA
5441	POLR2L	POLR2L	POLYMERASE (RNA) II (DNA DIRECTED) POLYPEPTIDE L, 7.6KDA
11128	POLR3A	POLR3A	POLYMERASE (RNA) III (DNA DIRECTED) POLYPEPTIDE A, 155KDA
55703	POLR3B	POLR3B	POLYMERASE (RNA) III (DNA DIRECTED) POLYPEPTIDE B
10623	POLR3C	POLR3C	POLYMERASE (RNA) III (DNA DIRECTED) POLYPEPTIDE C (62KD)
661	POLR3D	POLR3D	POLYMERASE (RNA) III (DNA DIRECTED) POLYPEPTIDE D, 44KDA
55718	POLR3E	POLR3E	POLYMERASE (RNA) III (DNA DIRECTED) POLYPEPTIDE E (80KD)
10621	POLR3F	POLR3F	POLYMERASE (RNA) III (DNA DIRECTED) POLYPEPTIDE F, 39 KDA
171568	POLR3H	POLR3H	POLYMERASE (RNA) III (DNA DIRECTED) POLYPEPTIDE H (22.9KD)
51728	POLR3K	POLR3K	POLYMERASE (RNA) III (DNA DIRECTED) POLYPEPTIDE K, 12.3 KDA
5449	POU1F1	POU1F1	POU DOMAIN, CLASS 1, TRANSCRIPTION FACTOR 1 (PIT1, GROWTH HORMONE FACTOR 1)
5450	POU2AF1	POU2AF1	POU DOMAIN, CLASS 2, ASSOCIATING FACTOR 1
5451	POU2F1	POU2F1	POU DOMAIN, CLASS 2, TRANSCRIPTION FACTOR 1
5452	POU2F2	POU2F2	POU DOMAIN, CLASS 2, TRANSCRIPTION FACTOR 2
25833	POU2F3	POU2F3	POU DOMAIN, CLASS 2, TRANSCRIPTION FACTOR 3
5453	POU3F1	POU3F1	POU DOMAIN, CLASS 3, TRANSCRIPTION FACTOR 1
5454	POU3F2	POU3F2	POU DOMAIN, CLASS 3, TRANSCRIPTION FACTOR 2
5455	POU3F3	POU3F3	POU DOMAIN, CLASS 3, TRANSCRIPTION FACTOR 3
5456	POU3F4	POU3F4	POU DOMAIN, CLASS 3, TRANSCRIPTION FACTOR 4
5457	POU4F1	POU4F1	POU DOMAIN, CLASS 4, TRANSCRIPTION FACTOR 1
5458	POU4F2	POU4F2	POU DOMAIN, CLASS 4, TRANSCRIPTION FACTOR 2
5459	POU4F3	POU4F3	POU DOMAIN, CLASS 4, TRANSCRIPTION FACTOR 3
5460	POU5F1	POU5F1	POU DOMAIN, CLASS 5, TRANSCRIPTION FACTOR 1
5462	POU5F1P1	POU5F1P1	POU DOMAIN, CLASS 5, TRANSCRIPTION FACTOR 1 PSEUDOGENE 1
134187	FLJ25680	POU5F2	POU domain class 5, transcription factor 2
5463	POU6F1	POU6F1	POU DOMAIN, CLASS 6, TRANSCRIPTION FACTOR 1
11281	POU6F2	POU6F2	POU DOMAIN, CLASS 6, TRANSCRIPTION FACTOR 2
56342	PPAN	PPAN	PETER PAN HOMOLOG (DROSOPHILA)
5465	PPARA	PPARA	PEROXISOME PROLIFERATIVE ACTIVATED RECEPTOR, ALPHA
5469	PPARBP	PPARBP	PPAR BINDING PROTEIN
5467	PPARD	PPARD	PEROXISOME PROLIFERATIVE ACTIVATED RECEPTOR, DELTA
5468	PPARG	PPARG	PEROXISOME PROLIFERATIVE ACTIVATED RECEPTOR, GAMMA
10891	PPARGC1A	PPARGC1A	PEROXISOME PROLIFERATIVE ACTIVATED RECEPTOR, GAMMA, COACTIVATOR 1, ALPHA
133522	PPARGC1B	PPARGC1B	PEROXISOME PROLIFERATIVE ACTIVATED RECEPTOR, GAMMA, COACTIVATOR 1, BETA
10084	PQBP1	PQBP1	POLYGLUTAMINE BINDING PROTEIN 1
639	PRDM1	PRDM1	PR DOMAIN CONTAINING 1, WITH ZNF DOMAIN
56980	PRDM10	PRDM10	PR DOMAIN CONTAINING 10
59335	PRDM12	PRDM12	PR DOMAIN CONTAINING 12
59336	PRDM13	PRDM13	PR DOMAIN CONTAINING 13
63978	PRDM14	PRDM14	PR DOMAIN CONTAINING 14
63976	PRDM16	PRDM16	PR DOMAIN CONTAINING 16
7799	PRDM2	PRDM2	PR DOMAIN CONTAINING 2, WITH ZNF DOMAIN
11108	PRDM4	PRDM4	PR DOMAIN CONTAINING 4
11107	PRDM5	PRDM5	PR DOMAIN CONTAINING 5
93166	PRDM6	PRDM6	PR DOMAIN CONTAINING 6
11105	PRDM7	PRDM7	PR DOMAIN CONTAINING 7
56978	PRDM8	PRDM8	PR DOMAIN CONTAINING 8
56979	PRDM9	PRDM9	PR DOMAIN CONTAINING 9
10113	PREB	PREB	PROLACTIN REGULATORY ELEMENT BINDING
85441	PRIC285	PRIC285	PEROXISOMAL PROLIFERATOR-ACTIVATED RECEPTOR A INTERACTING COMPLEX 285
5626	PROP1	PROP1	PROPHET OF PIT1, PAIRED-LIKE HOMEODOMAIN TRANSCRIPTION FACTOR
5629	PROX1	PROX1	PROSPERO-RELATED HOMEOBOX 1
8899	PRPF4B	PRPF4B	PRP4 PRE-MRNA PROCESSING FACTOR 4 HOMOLOG B (YEAST)
5396	PRRX1	PRRX1	PAIRED RELATED HOMEOBOX 1
51450	PRRX2	PRRX2	PAIRED RELATED HOMEOBOX 2
11168	PSIP1	PSIP1	PC4 AND SFRS1 INTERACTING PROTEIN 1
256297	PTF1A	PTF1A	PANCREAS SPECIFIC TRANSCRIPTION FACTOR, 1A
5757	PTMA	PTMA	PROTHYMOSIN, ALPHA (GENE SEQUENCE 28)
284119	PTRF	PTRF	POLYMERASE I AND TRANSCRIPT RELEASE FACTOR
9232	PTTG1	PTTG1	PITUITARY TUMOR-TRANSFORMING 1
5813	PURA	PURA	PURINE-RICH ELEMENT BINDING PROTEIN A
11137	PWP1	PWP1	PWP1 HOMOLOG (S. CEREVISIAE)
51547	SIRT7	PYCR1	PYRROLINE-5-CARBOXYLATE REDUCTASE 1
26108	PYGO1	PYGO1	PYGOPUS HOMOLOG 1 (DROSOPHILA)
90780	PYGO2	PYGO2	PYGOPUS HOMOLOG 2 (DROSOPHILA)
5888	RAD51	RAD51	RAD51 HOMOLOG (RECA HOMOLOG, E. COLI) (S. CEREVISIAE)
5890	RAD51L1	RAD51L1	RAD51-LIKE 1 (S. CEREVISIAE)
25788	RAD54B	RAD54B	FIBRINOGEN SILENCER BINDING PROTEIN
10743	RAI1	RAI1	RETINOIC ACID INDUCED 1
26064	RAI14	RAI14	RETINOIC ACID INDUCED 14
51720	RAP80	RAP80	RECEPTOR ASSOCIATED PROTEIN 80
5914	RARA	RARA	RETINOIC ACID RECEPTOR, ALPHA
5915	RARB	RARB	RETINOIC ACID RECEPTOR, BETA
5916	RARG	RARG	RETINOIC ACID RECEPTOR, GAMMA
30062	RAX	RAX	RETINA AND ANTERIOR NEURAL FOLD HOMEOBOX
84839	RAXL1	RAXL1	RETINA AND ANTERIOR NEURAL FOLD HOMEOBOX LIKE 1
5925	RB1	RB1	RETINOBLASTOMA 1 (INCLUDING OSTEOSARCOMA)
57786	RBAK	RBAK	RB-ASSOCIATED KRAB REPRESSOR
5928	RBBP4	RBBP4	RETINOBLASTOMA BINDING PROTEIN 4
5931	RBBP7	RBBP7	RETINOBLASTOMA BINDING PROTEIN 7
10616	C20orf18	RBCK1	RanBP-type and C3HC4-type zinc finger containing 1
5933	RBL1	RBL1	RETINOBLASTOMA-LIKE 1 (P107)
5934	RBL2	RBL2	RETINOBLASTOMA-LIKE 2 (P130)
10432	RBM14	RBM14	RNA BINDING MOTIF PROTEIN 14
64783	RBM15	RBM15	RNA BINDING MOTIF PROTEIN 15
29890	RBM15B	RBM15B	RNA BINDING MOTIF PROTEIN 15B
22828	RBM16	RBM16	RNA BINDING MOTIF PROTEIN 16
10179	RBM7	RBM7	RNA BINDING MOTIF PROTEIN 7
23543	RBM9	RBM9	RNA BINDING MOTIF PROTEIN 9
27316	RBMX	RBMX	RNA BINDING MOTIF PROTEIN, X-LINKED
3516	RBPSUH	RBPSUH	RECOMBINING BINDING PROTEIN SUPPRESSOR OF HAIRLESS (DROSOPHILA)
11317	RBPSUHL	RBPSUHL	RECOMBINING BINDING PROTEIN SUPPRESSOR OF HAIRLESS (DROSOPHILA)-LIKE
283248	RCOR2	RCOR2	REST COREPRESSOR 2
7936	RDBP	RDBP	RD RNA BINDING PROTEIN
5966	REL	REL	V-REL RETICULOENDOTHELIOSIS VIRAL ONCOGENE HOMOLOG (AVIAN)
5970	RELA	RELA	V-REL RETICULOENDOTHELIOSIS VIRAL ONCOGENE HOMOLOG A, NUCLEAR FACTOR OF KAPPA LIGHT POLYPEPTIDE GENE ENHANCER IN B-CELLS 3, P65 (AV
5971	RELB	RELB	V-REL RETICULOENDOTHELIOSIS VIRAL ONCOGENE HOMOLOG B, NUCLEAR FACTOR OF KAPPA LIGHT POLYPEPTIDE GENE ENHANCER IN B-CELLS 3 (AVIAN)
473	RERE	RERE	ARGININE-GLUTAMIC ACID DIPEPTIDE (RE) REPEATS
5978	REST	REST	RE1-SILENCING TRANSCRIPTION FACTOR
57109	REXO4	REXO4	REX4, RNA EXONUCLEASE 4 HOMOLOG (S. CEREVISIAE)
5981	RFC1	RFC1	REPLICATION FACTOR C (ACTIVATOR 1) 1, 145KDA
5987	RFP	RFP	RET FINGER PROTEIN
5989	RFX1	RFX1	REGULATORY FACTOR X, 1 (INFLUENCES HLA CLASS II EXPRESSION)
5990	RFX2	RFX2	REGULATORY FACTOR X, 2 (INFLUENCES HLA CLASS II EXPRESSION)
5991	RFX3	RFX3	REGULATORY FACTOR X, 3 (INFLUENCES HLA CLASS II EXPRESSION)
5992	RFX4	RFX4	REGULATORY FACTOR X, 4 (INFLUENCES HLA CLASS II EXPRESSION)
5993	RFX5	RFX5	REGULATORY FACTOR X, 5 (INFLUENCES HLA CLASS II EXPRESSION)
8625	RFXANK	RFXANK	REGULATORY FACTOR X-ASSOCIATED ANKYRIN-CONTAINING PROTEIN
5994	RFXAP	RFXAP	REGULATORY FACTOR X-ASSOCIATED PROTEIN
222546	RFXDC1	RFXDC1	REGULATORY FACTOR X DOMAIN CONTAINING 1

64864	RFXDC2	RFXDC2	REGULATORY FACTOR X DOMAIN CONTAINING 2
10636	RGS14	RGS14	REGULATOR OF G-PROTEIN SIGNALLING 14
6015	RING1	RING1	RING FINGER PROTEIN 1
6018	RLF	RLF	REARRANGED L-MYC FUSION
51132	RNF12	RNF12	RING FINGER PROTEIN 12
9604	RNF14	RNF14	RING FINGER PROTEIN 14
50862	RNF141	RNF141	RING FINGER PROTEIN 141
25897	RNF19	RNF19	RING FINGER PROTEIN 19
6045	RNF2	RNF2	RING FINGER PROTEIN 2
10379	ISGF3G	RNF31	INTERFERON-STIMULATED TRANSCRIPTION FACTOR 3, GAMMA 48KDA
6047	RNF4	RNF4	RING FINGER PROTEIN 4
6049	RNF6	RNF6	RING FINGER PROTEIN (C3H2C3 TYPE) 6
9584	RNPC2	RNPC2	RNA-BINDING REGION (RNP1, RRM) CONTAINING 2
10921	RNPS1	RNPS1	RNA BINDING PROTEIN S1, SERINE-RICH DOMAIN
6095	RORA	RORA	RAR-RELATED ORPHAN RECEPTOR A
6096	RORB	RORB	RAR-RELATED ORPHAN RECEPTOR B
6097	RORC	RORC	RAR-RELATED ORPHAN RECEPTOR C
286380	RP11-561O23.3	RP11-561O23.3	FOXD4-LIKE 2
8227	RP13-297E16.1	RP13-297E16.1	DNA SEGMENT ON CHROMOSOME X AND Y (UNIQUE) 155 EXPRESSED SEQUENCE, ISOFORM 1
6239	RREB1	RREB1	RAS RESPONSIVE ELEMENT BINDING PROTEIN 1
861	RUNX1	RUNX1	RUNT-RELATED TRANSCRIPTION FACTOR 1 (ACUTE MYELOID LEUKEMIA 1; AML1 ONCOGENE)
862	RUNX1T1	RUNX1T1	RUNT-RELATED TRANSCRIPTION FACTOR 1; TRANSLOCATED TO, 1 (CYCLIN D-RELATED)
860	RUNX2	RUNX2	RUNT-RELATED TRANSCRIPTION FACTOR 2
864	RUNX3	RUNX3	RUNT-RELATED TRANSCRIPTION FACTOR 3
8607	RUVBL1	RUVBL1	RUVB-LIKE 1 (E. COLI)
10856	RUVBL2	RUVBL2	RUVB-LIKE 2 (E. COLI)
6256	RXRA	RXRA	RETINOID X RECEPTOR, ALPHA
6257	RXRB	RXRB	RETINOID X RECEPTOR, BETA
6258	RXRG	RXRG	RETINOID X RECEPTOR, GAMMA
11017	RY1	RY1	PUTATIVE NUCLEIC ACID BINDING PROTEIN RY-1
23429	RYBP	RYBP	RING1 AND YY1 BINDING PROTEIN
6294	SAFB	SAFB	SCAFFOLD ATTACHMENT FACTOR B
9667	SAFB2	SAFB2	SCAFFOLD ATTACHMENT FACTOR B2
286749	SALF	SALF	STONIN 1
6299	SALL1	SALL1	SAL-LIKE 1 (DROSOPHILA)
6297	SALL2	SALL2	SAL-LIKE 2 (DROSOPHILA)
27164	SALL3	SALL3	SAL-LIKE 3 (DROSOPHILA)
57167	SALL4	SALL4	SAL-LIKE 4 (DROSOPHILA)
10284	SAP18	SAP18	SIN3A-ASSOCIATED PROTEIN, 18KDA
8819	SAP30	SAP30	SIN3A-ASSOCIATED PROTEIN, 30KDA
29115	SAP30BP	SAP30BP	SAP30 BINDING PROTEIN
6304	SATB1	SATB1	SPECIAL AT-RICH SEQUENCE BINDING PROTEIN 1 (BINDS TO NUCLEAR MATRIX/SCAFFOLD-ASSOCIATING DNA'S)
23314	SATB2	SATB2	SATB FAMILY MEMBER 2
51282	SCAND1	SCAND1	SCAN DOMAIN CONTAINING 1
54581	SCAND2	SCAND2	SCAN DOMAIN CONTAINING 2
22955	SCMH1	SCMH1	SEX COMB ON MIDLEG HOMOLOG 1 (DROSOPHILA)
6322	SCML1	SCML1	SEX COMB ON MIDLEG-LIKE 1 (DROSOPHILA)
10389	SCML2	SCML2	SEX COMB ON MIDLEG-LIKE 2 (DROSOPHILA)
83482	SCRT1	SCRT1	SCRATCH HOMOLOG 1, ZINC FINGER PROTEIN (DROSOPHILA)
85508	SCRT2	SCRT2	SCRATCH HOMOLOG 2, ZINC FINGER PROTEIN (DROSOPHILA)
23541	SEC14L2	SEC14L2	SEC14-LIKE 2 (S. CEREVISIAE)
29950	SERTAD1	SERTAD1	SERTA DOMAIN CONTAINING 1
9792	SERTAD2	SERTAD2	SERTA DOMAIN CONTAINING 2
29946	SERTAD3	SERTAD3	SERTA DOMAIN CONTAINING 3
26040	SETBP1	SETBP1	SET BINDING PROTEIN 1
7536	SF1	SF1	SPLICING FACTOR 1
57794	SF4	SF4	SPLICING FACTOR 4
51460	SFMBT1	SFMBT1	Scm-like with four mbt domains 1
6421	SFPQ	SFPQ	SPLICING FACTOR PROLINE/GLUTAMINE-RICH (POLYPYRIMIDINE TRACT BINDING PROTEIN ASSOCIATED)
6426	SFRS1	SFRS1	SPLICING FACTOR, ARGININE/SERINE-RICH 1 (SPLICING FACTOR 2, ALTERNATE SPLICING FACTOR)
10147	SFRS14	SFRS14	SPLICING FACTOR, ARGININE/SERINE-RICH 14
11129	SFRS16	SFRS16	SPLICING FACTOR, ARGININE/SERINE-RICH 16 (SUPPRESSOR-OF-WHITE-APRICOT HOMOLOG, DROSOPHILA)
6427	SFRS2	SFRS2	SPLICING FACTOR, ARGININE/SERINE-RICH 2
6428	SFRS3	SFRS3	SPLICING FACTOR, ARGININE/SERINE-RICH 3
6430	SFRS5	SFRS5	SPLICING FACTOR, ARGININE/SERINE-RICH 5
6431	SFRS6	SFRS6	SPLICING FACTOR, ARGININE/SERINE-RICH 6
6433	SFRS8	SFRS8	SPLICING FACTOR, ARGININE/SERINE-RICH 8 (SUPPRESSOR-OF-WHITE-APRICOT HOMOLOG, DROSOPHILA)
8683	SFRS9	SFRS9	SPLICING FACTOR, ARGININE/SERINE-RICH 9
9646	SH2BP1	SH2BP1	SH2 DOMAIN BINDING PROTEIN 1 (TETRATRICOPEPTIDE REPEAT CONTAINING)
6473	SHOX1	SHOX	SHORT STATURE HOMEOBOX
6474	SHOX2	SHOX2	SHORT STATURE HOMEOBOX 2
257218	SHPRH	SHPRH	SNF2 HISTONE LINKER PHD RING HELICASE
6492	SIM1	SIM1	SINGLE-MINDED HOMOLOG 1 (DROSOPHILA)
6493	SIM2	SIM2	SINGLE-MINDED HOMOLOG 2 (DROSOPHILA)
25942	SIN3A	SIN3A	SIN3 HOMOLOG A, TRANSCRIPTION REGULATOR (YEAST)
23309	SIN3B	SIN3B	SIN3 HOMOLOG B, TRANSCRIPTION REGULATOR (YEAST)
23411	SIRT1	SIRT1	SIRTUIN (SILENT MATING TYPE INFORMATION REGULATION 2 HOMOLOG) 1 (S. CEREVISIAE)
22933	SIRT2	SIRT2	SIRTUIN (SILENT MATING TYPE INFORMATION REGULATION 2 HOMOLOG) 2 (S. CEREVISIAE)
23410	SIRT3	SIRT3	SIRTUIN (SILENT MATING TYPE INFORMATION REGULATION 2 HOMOLOG) 3 (S. CEREVISIAE)
23409	SIRT4	SIRT4	SIRTUIN (SILENT MATING TYPE INFORMATION REGULATION 2 HOMOLOG) 4 (S. CEREVISIAE)
23408	SIRT5	SIRT5	SIRTUIN (SILENT MATING TYPE INFORMATION REGULATION 2 HOMOLOG) 5 (S. CEREVISIAE)
6495	SIX1	SIX1	SINE OCULIS HOMEOBOX HOMOLOG 1 (DROSOPHILA)
10736	SIX2	SIX2	SINE OCULIS HOMEOBOX HOMOLOG 2 (DROSOPHILA)
6496	SIX3	SIX3	SINE OCULIS HOMEOBOX HOMOLOG 3 (DROSOPHILA)
51804	SIX4	SIX4	SINE OCULIS HOMEOBOX HOMOLOG 4 (DROSOPHILA)
147912	SIX5	SIX5	SINE OCULIS HOMEOBOX HOMOLOG 5 (DROSOPHILA)
4990	SIX6	SIX6	SINE OCULIS HOMEOBOX HOMOLOG 6 (DROSOPHILA)
6497	SKI	SKI	V-SKI SARCOMA VIRAL ONCOGENE HOMOLOG (AVIAN)
6498	SKIL	SKIL	SKI-LIKE
84174	SLA2	SLA2	SRC-LIKE-ADAPTOR 2
56731	SLC2A4RG	SLC2A4RG	SLC2A4 REGULATOR
4086	SMAD1	SMAD1	SMAD, MOTHERS AGAINST DPP HOMOLOG 1 (DROSOPHILA)
4087	SMAD2	SMAD2	SMAD, MOTHERS AGAINST DPP HOMOLOG 2 (DROSOPHILA)
4088	SMAD3	SMAD3	SMAD, MOTHERS AGAINST DPP HOMOLOG 3 (DROSOPHILA)
4089	SMAD4	SMAD4	SMAD, MOTHERS AGAINST DPP HOMOLOG 4 (DROSOPHILA)
4090	SMAD5	SMAD5	SMAD, MOTHERS AGAINST DPP HOMOLOG 5 (DROSOPHILA)
4091	SMAD6	SMAD6	SMAD, MOTHERS AGAINST DPP HOMOLOG 6 (DROSOPHILA)
4092	SMAD7	SMAD7	SMAD, MOTHERS AGAINST DPP HOMOLOG 7 (DROSOPHILA)
4093	SMAD9	SMAD9	SMAD, MOTHERS AGAINST DPP HOMOLOG 9 (DROSOPHILA)
6594	SMARCA1	SMARCA1	SWI/SNF RELATED, MATRIX ASSOCIATED, ACTIN DEPENDENT REGULATOR OF CHROMATIN, SUBFAMILY A, MEMBER 1
6595	SMARCA2	SMARCA2	SWI/SNF RELATED, MATRIX ASSOCIATED, ACTIN DEPENDENT REGULATOR OF CHROMATIN, SUBFAMILY A, MEMBER 2
6596	SMARCA3	SMARCA3	SWI/SNF RELATED, MATRIX ASSOCIATED, ACTIN DEPENDENT REGULATOR OF CHROMATIN, SUBFAMILY A, MEMBER 3
6597	SMARCA4	SMARCA4	SWI/SNF RELATED, MATRIX ASSOCIATED, ACTIN DEPENDENT REGULATOR OF CHROMATIN, SUBFAMILY A, MEMBER 4
8467	SMARCA5	SMARCA5	SWI/SNF RELATED, MATRIX ASSOCIATED, ACTIN DEPENDENT REGULATOR OF CHROMATIN, SUBFAMILY A, MEMBER 5
56916	SMARCAD1	SMARCAD1	SWI/SNF-RELATED, MATRIX-ASSOCIATED ACTIN-DEPENDENT REGULATOR OF CHROMATIN, SUBFAMILY A, CONTAINING DEAD/H BOX 1
6598	SMARCB1	SMARCB1	SWI/SNF RELATED, MATRIX ASSOCIATED, ACTIN DEPENDENT REGULATOR OF CHROMATIN, SUBFAMILY B, MEMBER 1
6599	SMARCC1	SMARCC1	SWI/SNF RELATED, MATRIX ASSOCIATED, ACTIN DEPENDENT REGULATOR OF CHROMATIN, SUBFAMILY C, MEMBER 1
6601	SMARCC2	SMARCC2	SWI/SNF RELATED, MATRIX ASSOCIATED, ACTIN DEPENDENT REGULATOR OF CHROMATIN, SUBFAMILY C, MEMBER 2
6602	SMARCD1	SMARCD1	SWI/SNF RELATED, MATRIX ASSOCIATED, ACTIN DEPENDENT REGULATOR OF CHROMATIN, SUBFAMILY D, MEMBER 1
6603	SMARCD2	SMARCD2	SWI/SNF RELATED, MATRIX ASSOCIATED, ACTIN DEPENDENT REGULATOR OF CHROMATIN, SUBFAMILY D, MEMBER 2
6604	SMARCD3	SMARCD3	SWI/SNF RELATED, MATRIX ASSOCIATED, ACTIN DEPENDENT REGULATOR OF CHROMATIN, SUBFAMILY D, MEMBER 3
6605	SMARCE1	SMARCE1	SWI/SNF RELATED, MATRIX ASSOCIATED, ACTIN DEPENDENT REGULATOR OF CHROMATIN, SUBFAMILY E, MEMBER 1
8242	SMCX	SMCX	SMCY HOMOLOG, X-LINKED (MOUSE)
8284	SMCY	SMCY	SMCY HOMOLOG, Y-LINKED (MOUSE)
64750	SMURF2	SMURF2	SMAD SPECIFIC E3 UBIQUITIN PROTEIN LIGASE 2
150572	SMYD1	SMYD1	SET AND MYND DOMAIN CONTAINING 1
6615	SNAI1	SNAI1	SNAIL HOMOLOG 1 (DROSOPHILA)
6591	SNAI2	SNAI2	SNAIL HOMOLOG 2 (DROSOPHILA)
333929	SNAI3	SNAI3	SNAIL HOMOLOG 3 (DROSOPHILA)
6617	SNAPC1	SNAPC1	SMALL NUCLEAR RNA ACTIVATING COMPLEX, POLYPEPTIDE 1, 43KDA
6618	SNAPC2	SNAPC2	SMALL NUCLEAR RNA ACTIVATING COMPLEX, POLYPEPTIDE 2, 45KDA
6619	SNAPC3	SNAPC3	SMALL NUCLEAR RNA ACTIVATING COMPLEX, POLYPEPTIDE 3, 50KDA
6621	SNAPC4	SNAPC4	SMALL NUCLEAR RNA ACTIVATING COMPLEX, POLYPEPTIDE 4, 190KDA
10302	SNAPC5	SNAPC5	SMALL NUCLEAR RNA ACTIVATING COMPLEX, POLYPEPTIDE 5, 19KDA
27044	SND1	SND1	STAPHYLOCOCCAL NUCLEASE DOMAIN CONTAINING 1
55509	SNFT	SNFT	JUN DIMERIZATION PROTEIN P21SNFT
79753	SNIP1	SNIP1	SMAD NUCLEAR INTERACTING PROTEIN 1
6628	SNRPB	SNRPB	SMALL NUCLEAR RIBONUCLEOPROTEIN POLYPEPTIDES B AND B1
6632	SNRPD1	SNRPD1	SMALL NUCLEAR RIBONUCLEOPROTEIN D1 POLYPEPTIDE 16KDA

22938	SNW1	SNW1	SNW DOMAIN CONTAINING 1
6650	SOLH	SOLH	SMALL OPTIC LOBES HOMOLOG (DROSOPHILA)
6656	SOX1	SOX1	SRY (SEX DETERMINING REGION Y)-BOX 1
6663	SOX10	SOX10	SRY (SEX DETERMINING REGION Y)-BOX 10
6664	SOX11	SOX11	SRY (SEX DETERMINING REGION Y)-BOX 11
6666	SOX12	SOX12	SRY (SEX DETERMINING REGION Y)-BOX 12
9580	SOX13	SOX13	SRY (SEX DETERMINING REGION Y)-BOX 13
8403	SOX14	SOX14	SRY (SEX DETERMINING REGION Y)-BOX 14
6665	SOX15	SOX15	SRY (SEX DETERMINING REGION Y)-BOX 15
64321	SOX17	SOX17	SRY (SEX DETERMINING REGION Y)-BOX 17
6657	SOX2	SOX2	SRY (SEX DETERMINING REGION Y)-BOX 2
11166	SOX21	SOX21	SRY (SEX DETERMINING REGION Y)-BOX 21
6658	SOX3	SOX3	SRY (SEX DETERMINING REGION Y)-BOX 3
11063	SOX30	SOX30	SRY (SEX DETERMINING REGION Y)-BOX 30
6659	SOX4	SOX4	SRY (SEX DETERMINING REGION Y)-BOX 4
6660	SOX5	SOX5	SRY (SEX DETERMINING REGION Y)-BOX 5
55553	SOX6	SOX6	SRY (SEX DETERMINING REGION Y)-BOX 6
83595	SOX7	SOX7	SRY (SEX DETERMINING REGION Y)-BOX 7
30812	SOX8	SOX8	SRY (SEX DETERMINING REGION Y)-BOX 8
6662	SOX9	SOX9	SRY (SEX DETERMINING REGION Y)-BOX 9 (CAMPOMELIC DYSPLASIA, AUTOSOMAL SEX-REVERSAL)
6667	SP1	SP1	SP1 TRANSCRIPTION FACTOR
6672	SP100	SP100	SP100 NUCLEAR ANTIGEN
3431	SP110	SP110	SP110 NUCLEAR BODY PROTEIN
11262	SP140	SP140	SP140 NUCLEAR BODY PROTEIN
6668	SP2	SP2	SP2 TRANSCRIPTION FACTOR
6670	SP3	SP3	SP3 TRANSCRIPTION FACTOR
6671	SP4	SP4	SP4 TRANSCRIPTION FACTOR
121340	SP7	SP7	SP7 TRANSCRIPTION FACTOR
221833	SP8	SP8	SP8 TRANSCRIPTION FACTOR
25803	SPDEF	SPDEF	SAM POINTED DOMAIN CONTAINING ETS TRANSCRIPTION FACTOR
23013	SPEN	SPEN	SPEN HOMOLOG, TRANSCRIPTIONAL REGULATOR (DROSOPHILA)
6688	SPI1	SPI1	SPLEEN FOCUS FORMING VIRUS (SFFV) PROVIRAL INTEGRATION ONCOGENE SPI1
6689	SPIB	SPIB	SPI-B TRANSCRIPTION FACTOR (SPI-1/PU.1 RELATED)
121599	SPIC	SPIC	SPI-C TRANSCRIPTION FACTOR (SPI-1/PU.1 RELATED)
23626	SPO11	SPO11	SPO11 MEIOTIC PROTEIN COVALENTLY BOUND TO DSB-LIKE (S. CEREVISIAE)
90853	SPOCD1	SPOCD1	SPOC DOMAIN CONTAINING 1
84654	SPZ1	SPZ1	SPERMATOGENIC LEUCINE ZIPPER 1
58506	SR-A1	SR-A1	SERINE ARGININE-RICH PRE-MRNA SPLICING FACTOR SR-A1
10847	SRCAP	SRCAP	SNF2-RELATED CBP ACTIVATOR PROTEIN
6720	SREBF1	SREBF1	STEROL REGULATORY ELEMENT BINDING TRANSCRIPTION FACTOR 1
6721	SREBF2	SREBF2	STEROL REGULATORY ELEMENT BINDING TRANSCRIPTION FACTOR 2
6722	SRF	SRF	SERUM RESPONSE FACTOR (C-FOS SERUM RESPONSE ELEMENT-BINDING TRANSCRIPTION FACTOR)
10929	SFRS2B	SRP46	SPLICING FACTOR, ARGININE/SERINE-RICH, 46KD
10250	SRRM1	SRRM1	SERINE/ARGININE REPETITIVE MATRIX 1
6736	SRY	SRY	SEX DETERMINING REGION Y
26039	SS18L1	SS18L1	SYNOVIAL SARCOMA TRANSLOCATION GENE ON CHROMOSOME 18-LIKE 1
6741	SSB	SSB	SJOGREN SYNDROME ANTIGEN B (AUTOANTIGEN LA)
23635	SSBP2	SSBP2	SINGLE-STRANDED DNA BINDING PROTEIN 2
23648	SSBP3	SSBP3	SINGLE STRANDED DNA BINDING PROTEIN 3
170463	SSBP4	SSBP4	SINGLE STRANDED DNA BINDING PROTEIN 4
6749	SSRP1	SSRP1	STRUCTURE SPECIFIC RECOGNITION PROTEIN 1
6756	SSX1	SSX1	SYNOVIAL SARCOMA, X BREAKPOINT 1
6757	SSX2	SSX2	SYNOVIAL SARCOMA, X BREAKPOINT 2
10214	SSX3	SSX3	SYNOVIAL SARCOMA, X BREAKPOINT 3
6759	SSX4	SSX4	SYNOVIAL SARCOMA, X BREAKPOINT 4
6758	SSX5	SSX5	SYNOVIAL SARCOMA, X BREAKPOINT 5
9705	ST18	ST18	SUPPRESSION OF TUMORIGENICITY 18 (BREAST CARCINOMA) (ZINC FINGER PROTEIN)
6772	STAT1	STAT1	SIGNAL TRANSDUCER AND ACTIVATOR OF TRANSCRIPTION 1, 91KDA
6773	STAT2	STAT2	SIGNAL TRANSDUCER AND ACTIVATOR OF TRANSCRIPTION 2, 113KDA
6774	STAT3	STAT3	SIGNAL TRANSDUCER AND ACTIVATOR OF TRANSCRIPTION 3 (ACUTE-PHASE RESPONSE FACTOR)
6775	STAT4	STAT4	SIGNAL TRANSDUCER AND ACTIVATOR OF TRANSCRIPTION 4
6776	STAT5A	STAT5A	SIGNAL TRANSDUCER AND ACTIVATOR OF TRANSCRIPTION 5A
6777	STAT5B	STAT5B	SIGNAL TRANSDUCER AND ACTIVATOR OF TRANSCRIPTION 5B
6778	STAT6	STAT6	SIGNAL TRANSDUCER AND ACTIVATOR OF TRANSCRIPTION 6, INTERLEUKIN-4 INDUCED
10923	SUB1	SUB1	SUB1 HOMOLOG (S. CEREVISIAE)
51684	SUFU	SUFU	SUPPRESSOR OF FUSED HOMOLOG (DROSOPHILA)
129025	SUHW1	SUHW1	SUPPRESSOR OF HAIRY WING HOMOLOG 1 (DROSOPHILA)
140883	SUHW2	SUHW2	SUPPRESSOR OF HAIRY WING HOMOLOG 2 (DROSOPHILA)
11198	SUPT16H	SUPT16H	SUPPRESSOR OF TY 16 HOMOLOG (S. CEREVISIAE)
8464	SUPT3H	SUPT3H	SUPPRESSOR OF TY 3 HOMOLOG (S. CEREVISIAE)
6827	SUPT4H1	SUPT4H1	SUPPRESSOR OF TY 4 HOMOLOG 1 (S. CEREVISIAE)
6829	SUPT5H	SUPT5H	SUPPRESSOR OF TY 5 HOMOLOG (S. CEREVISIAE)
6830	SUPT6H	SUPT6H	SUPPRESSOR OF TY 6 HOMOLOG (S. CEREVISIAE)
9913	SUPT7L	SUPT7L	SUPPRESSOR OF TY 7 (S. CEREVISIAE)-LIKE
9412	SURB7	SURB7	SRB7 SUPPRESSOR OF RNA POLYMERASE B HOMOLOG (YEAST)
79723	SUV39H2	SUV39H2	SUPPRESSOR OF VARIEGATION 3-9 HOMOLOG 2 (DROSOPHILA)
23512	SUZ12	SUZ12	SUPPRESSOR OF ZESTE 12 HOMOLOG (DROSOPHILA)
6862	T	T	T, BRACHYURY HOMOLOG (MOUSE)
6871	TADA2L	TADA2L	TRANSCRIPTIONAL ADAPTOR 2 (ADA2 HOMOLOG, YEAST)-LIKE
10474	TADA3L	TADA3L	TRANSCRIPTIONAL ADAPTOR 3 (NGG1 HOMOLOG, YEAST)-LIKE
6872	TAF1	TAF1	TAF1 RNA POLYMERASE II, TATA BOX BINDING PROTEIN (TBP)-ASSOCIATED FACTOR, 250KDA
6881	TAF10	TAF10	TAF10 RNA POLYMERASE II, TATA BOX BINDING PROTEIN (TBP)-ASSOCIATED FACTOR, 30KDA
6882	TAF11	TAF11	TAF11 RNA POLYMERASE II, TATA BOX BINDING PROTEIN (TBP)-ASSOCIATED FACTOR, 28KDA
6883	TAF12	TAF12	TAF12 RNA POLYMERASE II, TATA BOX BINDING PROTEIN (TBP)-ASSOCIATED FACTOR, 20KDA
6884	TAF13	TAF13	TAF13 RNA POLYMERASE II, TATA BOX BINDING PROTEIN (TBP)-ASSOCIATED FACTOR, 18KDA
8148	TAF15	TAF15	TAF15 RNA POLYMERASE II, TATA BOX BINDING PROTEIN (TBP)-ASSOCIATED FACTOR, 68KDA
9015	TAF1A	TAF1A	TATA BOX BINDING PROTEIN (TBP)-ASSOCIATED FACTOR, RNA POLYMERASE I, A, 48KDA
9014	TAF1B	TAF1B	TATA BOX BINDING PROTEIN (TBP)-ASSOCIATED FACTOR, RNA POLYMERASE I, B, 63KDA
9013	TAF1C	TAF1C	TATA BOX BINDING PROTEIN (TBP)-ASSOCIATED FACTOR, RNA POLYMERASE I, C, 110KDA
138474	TAF1L	TAF1L	TAF1-LIKE RNA POLYMERASE II, TATA BOX BINDING PROTEIN (TBP)-ASSOCIATED FACTOR, 210KDA
6874	TAF4	TAF4	TAF4 RNA POLYMERASE II, TATA BOX BINDING PROTEIN (TBP)-ASSOCIATED FACTOR, 135KDA
6875	TAF4B	TAF4B	TAF4B RNA POLYMERASE II, TATA BOX BINDING PROTEIN (TBP)-ASSOCIATED FACTOR, 105KDA
6877	TAF5	TAF5	TAF5 RNA POLYMERASE II, TATA BOX BINDING PROTEIN (TBP)-ASSOCIATED FACTOR, 100KDA
27097	TAF5L	TAF5L	TAF5-LIKE RNA POLYMERASE II, P300/CBP-ASSOCIATED FACTOR (PCAF)-ASSOCIATED FACTOR, 65KDA
6878	TAF6	TAF6	TAF6 RNA POLYMERASE II, TATA BOX BINDING PROTEIN (TBP)-ASSOCIATED FACTOR, 80KDA
10629	TAF6L	TAF6L	TAF6-LIKE RNA POLYMERASE II, P300/CBP-ASSOCIATED FACTOR (PCAF)-ASSOCIATED FACTOR, 65KDA
6879	TAF7	TAF7	TAF7 RNA POLYMERASE II, TATA BOX BINDING PROTEIN (TBP)-ASSOCIATED FACTOR, 55KDA
6880	TAF9	TAF9	TAF9 RNA POLYMERASE II, TATA BOX BINDING PROTEIN (TBP)-ASSOCIATED FACTOR, 32KDA
51616	TAF9B	TAF9B	TAF9B RNA POLYMERASE II, TATA BOX BINDING PROTEIN (TBP)-ASSOCIATED FACTOR, 31KDA
6886	TAL1	TAL1	T-CELL ACUTE LYMPHOCYTIC LEUKEMIA 1
6887	TAL2	TAL2	T-CELL ACUTE LYMPHOCYTIC LEUKEMIA 2
6894	TARBP1	TARBP1	TAR (HIV-1) RNA BINDING PROTEIN 1
6895	TARBP2	TARBP2	TAR (HIV-1) RNA BINDING PROTEIN 2
23435	TARDBP	TARDBP	TAR DNA BINDING PROTEIN
30851	TAX1BP3	TAX1BP3	TAX1 (HUMAN T-CELL LEUKEMIA VIRUS TYPE I) BINDING PROTEIN 3
25771	TBC1D22A	TBC1D22A	TBC1 DOMAIN FAMILY, MEMBER 22A
23102	TBC1D2B	TBC1D2B	TBC1 DOMAIN FAMILY, MEMBER 2B
6907	TBL1X	TBL1X	TRANSDUCIN (BETA)-LIKE 1X-LINKED
6908	TBP	TBP	TATA BOX BINDING PROTEIN
9519	TBPL1	TBPL1	TBP-LIKE 1
10716	TBR1	TBR1	T-BOX, BRAIN, 1
6899	TBX1	TBX1	T-BOX 1
347853	TBX10	TBX10	T-BOX 10
6913	TBX15	TBX15	T-BOX 15
9096	TBX18	TBX18	T-BOX 18
9095	TBX19	TBX19	T-BOX 19
6909	TBX2	TBX2	T-BOX 2
57057	TBX20	TBX20	T-BOX 20
30009	TBX21	TBX21	TRANSCRIPTION FACTOR TBLYM
50945	TBX22	TBX22	T-BOX 22
6926	TBX3	TBX3	T-BOX 3 (ULNAR MAMMARY SYNDROME)
9496	TBX4	TBX4	T-BOX 4
6910	TBX5	TBX5	T-BOX 5
6911	TBX6	TBX6	T-BOX 6
6917	TCEA1	TCEA1	TRANSCRIPTION ELONGATION FACTOR A (SII), 1
6919	TCEA2	TCEA2	TRANSCRIPTION ELONGATION FACTOR A (SII), 2
6920	TCEA3	TCEA3	TRANSCRIPTION ELONGATION FACTOR A (SII), 3
9338	TCEAL1	TCEAL1	TRANSCRIPTION ELONGATION FACTOR A (SII)-LIKE 1
6921	TCEB1	TCEB1	TRANSCRIPTION ELONGATION FACTOR B (SIII), POLYPEPTIDE 1 (15KDA, ELONGIN C)

6923	TCEB2	TCEB2	TRANSCRIPTION ELONGATION FACTOR B (SIII), POLYPEPTIDE 2 (18KDA, ELONGIN B)
6924	TCEB3	TCEB3	TRANSCRIPTION ELONGATION FACTOR B (SIII), POLYPEPTIDE 3 (110KDA, ELONGIN A)
51224	TCEB3B	TCEB3B	TRANSCRIPTION ELONGATION FACTOR B POLYPEPTIDE 3B (ELONGIN A2)
162699	TCEB3C	TCEB3C	TRANSCRIPTION ELONGATION FACTOR B POLYPEPTIDE 3C (ELONGIN A3)
10915	TCERG1	TCERG1	TRANSCRIPTION ELONGATION REGULATOR 1
6927	TCF1	TCF1	TRANSCRIPTION FACTOR 1, HEPATIC; LF-B1, HEPATIC NUCLEAR FACTOR (HNF1), ALBUMIN PROXIMAL FACTOR
6938	TCF12	TCF12	TRANSCRIPTION FACTOR 12 (HTF4, HELIX-LOOP-HELIX TRANSCRIPTION FACTORS 4)
6939	TCF15	TCF15	TRANSCRIPTION FACTOR 15 (BASIC HELIX-LOOP-HELIX)
6941	TCF19	TCF19	TRANSCRIPTION FACTOR 19 (SC1)
6928	TCF2	TCF2	TRANSCRIPTION FACTOR 2, HEPATIC; LF-B3; VARIANT HEPATIC NUCLEAR FACTOR
6942	TCF20	TCF20	TRANSCRIPTION FACTOR 20 (AR1)
6943	TCF21	TCF21	TRANSCRIPTION FACTOR 21
6929	TCF3	TCF3	TRANSCRIPTION FACTOR 3 (E2A IMMUNOGLOBULIN ENHANCER BINDING FACTORS E12/E47)
6925	TCF4	TCF4	TRANSCRIPTION FACTOR 4
6932	TCF7	TCF7	TRANSCRIPTION FACTOR 7 (T-CELL SPECIFIC, HMG-BOX)
83439	TCF7L1	TCF7L1	TRANSCRIPTION FACTOR 7-LIKE 1 (T-CELL SPECIFIC, HMG-BOX)
6934	TCF7L2	TCF7L2	TRANSCRIPTION FACTOR 7-LIKE 2 (T-CELL SPECIFIC, HMG-BOX)
6935	TCF8	TCF8	TRANSCRIPTION FACTOR 8 (REPRESSES INTERLEUKIN 2 EXPRESSION)
10732	TCFL5	TCFL5	TRANSCRIPTION FACTOR-LIKE 5 (BASIC HELIX-LOOP-HELIX)
6949	TCOF1	TCOF1	TREACHER COLLINS-FRANCESCHETTI SYNDROME 1
7003	TEAD1	TEAD1	TEA DOMAIN FAMILY MEMBER 1 (SV40 TRANSCRIPTIONAL ENHANCER FACTOR)
8463	TEAD2	TEAD2	TEA DOMAIN FAMILY MEMBER 2
7005	TEAD3	TEAD3	TEA DOMAIN FAMILY MEMBER 3
7004	TEAD4	TEAD4	TEA DOMAIN FAMILY MEMBER 4
7008	TEF	TEF	THYROTROPHIC EMBRYONIC FACTOR
7020	TFAP2A	TFAP2A	TRANSCRIPTION FACTOR AP-2 ALPHA (ACTIVATING ENHANCER BINDING PROTEIN 2 ALPHA)
7021	TFAP2B	TFAP2B	TRANSCRIPTION FACTOR AP-2 BETA (ACTIVATING ENHANCER BINDING PROTEIN 2 BETA)
7022	TFAP2C	TFAP2C	TRANSCRIPTION FACTOR AP-2 GAMMA (ACTIVATING ENHANCER BINDING PROTEIN 2 GAMMA)
83741	TFAP2D	TFAP2D	TRANSCRIPTION FACTOR AP-2 DELTA (ACTIVATING ENHANCER BINDING PROTEIN 2 DELTA)
339488	TFAP2E	TFAP2E	TRANSCRIPTION FACTOR AP-2 EPSILON (ACTIVATING ENHANCER BINDING PROTEIN 2 EPSILON)
7023	TFAP4	TFAP4	TRANSCRIPTION FACTOR AP-4 (ACTIVATING ENHANCER BINDING PROTEIN 4)
7024	TFCP2	TFCP2	TRANSCRIPTION FACTOR CP2
29842	TFCP2L1	TFCP2L1	TRANSCRIPTION FACTOR CP2-LIKE 1
7027	TFDP1	TFDP1	TRANSCRIPTION FACTOR DP-1
7029	TFDP2	TFDP2	TRANSCRIPTION FACTOR DP-2 (E2F DIMERIZATION PARTNER 2)
51270	TFDP3	TFDP3	TRANSCRIPTION FACTOR DP FAMILY, MEMBER 3
7030	TFE3	TFE3	TRANSCRIPTION FACTOR BINDING TO IGHM ENHANCER 3
7942	TFEB	TFEB	TRANSCRIPTION FACTOR EB
22797	TFEC	TFEC	TRANSCRIPTION FACTOR EC
29844	TFPT	TFPT	TCF3 (E2A) FUSION PARTNER (IN CHILDHOOD LEUKEMIA)
7041	TGFB1I1	TGFB1I1	TRANSFORMING GROWTH FACTOR BETA 1 INDUCED TRANSCRIPT 1
7050	TGIF	TGIF	TGFB-INDUCED FACTOR (TALE FAMILY HOMEOBOX)
60436	TGIF2	TGIF2	TGFB-induced factor homeobox 2
90316	TGIF2LX	TGIF2LX	TGFB-INDUCED FACTOR 2-LIKE, X-LINKED
90655	TGIF2LY	TGIF2LY	TGFB-INDUCED FACTOR 2-LIKE, Y-LINKED
51497	TH1L	TH1L	TH1-LIKE (DROSOPHILA)
80764	THAP7	THAP7	THAP DOMAIN CONTAINING 7
10189	THOC4	THOC4	THO COMPLEX 4
7067	THRA	THRA	THYROID HORMONE RECEPTOR, ALPHA (ERYTHROBLASTIC LEUKEMIA VIRAL (V-ERB-A) ONCOGENE HOMOLOG, AVIAN)
9969	THRAP1	THRAP1	THYROID HORMONE RECEPTOR ASSOCIATED PROTEIN 1
9967	THRAP3	THRAP3	THYROID HORMONE RECEPTOR ASSOCIATED PROTEIN 3
10025	THRAP5	THRAP5	THYROID HORMONE RECEPTOR ASSOCIATED PROTEIN 5
90390	THRAP6	THRAP6	THYROID HORMONE RECEPTOR ASSOCIATED PROTEIN 6
7068	THRB	THRB	THYROID HORMONE RECEPTOR, BETA (ERYTHROBLASTIC LEUKEMIA VIRAL (V-ERB-A) ONCOGENE HOMOLOG 2, AVIAN)
7073	TIAL1	TIAL1	TIA1 CYTOTOXIC GRANULE-ASSOCIATED RNA BINDING PROTEIN-LIKE 1
201798	TIGD4	TIGD4	TIGGER TRANSPOSABLE ELEMENT DERIVED 4
8914	TIMELESS	TIMELESS	TIMELESS HOMOLOG (DROSOPHILA)
7080	TITF1	TITF1	THYROID TRANSCRIPTION FACTOR 1
7088	TLE1	TLE1	TRANSDUCIN-LIKE ENHANCER OF SPLIT 1 (E(SP1) HOMOLOG, DROSOPHILA)
7089	TLE2	TLE2	TRANSDUCIN-LIKE ENHANCER OF SPLIT 2 (E(SP1) HOMOLOG, DROSOPHILA)
7090	TLE3	TLE3	TRANSDUCIN-LIKE ENHANCER OF SPLIT 3 (E(SP1) HOMOLOG, DROSOPHILA)
79816	TLE6	TLE6	TRANSDUCIN-LIKE ENHANCER OF SPLIT 6 (E(SP1) HOMOLOG, DROSOPHILA)
3195	TLX1	TLX1	T-CELL LEUKEMIA HOMEOBOX 1
3196	TLX2	TLX2	T-CELL LEUKEMIA HOMEOBOX 2
30012	TLX3	TLX3	T-CELL LEUKEMIA HOMEOBOX 3
7110	TMF1	TMF1	TATA ELEMENT MODULATORY FACTOR 1
7128	TNFAIP3	TNFAIP3	TUMOR NECROSIS FACTOR, ALPHA-INDUCED PROTEIN 3
11189	TNRC4	TNRC4	TRINUCLEOTIDE REPEAT CONTAINING 4
27324	TNRC9	TNRC9	TRINUCLEOTIDE REPEAT CONTAINING 9
10140	TOB1	TOB1	TRANSDUCER OF ERBB2, 1
114034	TOE1	TOE1	target of EGR1, member 1 (nuclear)
7155	TOP2B	TOP2B	TOPOISOMERASE (DNA) II BETA 180KDA
10210	TOPORS	TOPORS	TOPOISOMERASE I BINDING, ARGININE/SERINE-RICH
9760	TOX	TOX	THYMUS HIGH MOBILITY GROUP BOX PROTEIN TOX
84969	C20orf100	TOX2	TOX high mobility group box family member 2
9878	C14orf92	TOX4	TOX high mobility group box family member 4
7157	TP53	TP53	TUMOR PROTEIN P53 (LI-FRAUMENI SYNDROME)
7158	TP53BP1	TP53BP1	TUMOR PROTEIN P53 BINDING PROTEIN, 1
7161	TP73	TP73	TUMOR PROTEIN P73
8626	TP73L	TP73L	TUMOR PROTEIN P73-LIKE
55809	TRERF1	TRERF1	TRANSCRIPTIONAL REGULATING FACTOR 1
9477	TRFP	TRFP	TRF (TATA BINDING PROTEIN-RELATED FACTOR)-PROXIMAL HOMOLOG (DROSOPHILA)
57761	TRIB3	TRIB3	TRIBBLES HOMOLOG 3 (DROSOPHILA)
89870	TRIM15	TRIM15	TRIPARTITE MOTIF-CONTAINING 15
10626	TRIM16	TRIM16	TRIPARTITE MOTIF-CONTAINING 16
6737	TRIM21	TRIM21	TRIPARTITE MOTIF-CONTAINING 21
10346	TRIM22	TRIM22	TRIPARTITE MOTIF-CONTAINING 22
8805	TRIM24	TRIM24	TRIPARTITE MOTIF-CONTAINING 24
7706	TRIM25	TRIM25	TRIPARTITE MOTIF-CONTAINING 25
10155	TRIM28	TRIM28	TRIPARTITE MOTIF-CONTAINING 28
23650	TRIM29	TRIM29	TRIPARTITE MOTIF-CONTAINING 29
11074	TRIM31	TRIM31	TRIPARTITE MOTIF-CONTAINING 31
22954	TRIM32	TRIM32	TRIPARTITE MOTIF-CONTAINING 32
51592	TRIM33	TRIM33	TRIPARTITE MOTIF-CONTAINING 33
85363	TRIM5	TRIM5	TRIPARTITE MOTIF-CONTAINING 5
9321	TRIP11	TRIP11	THYROID HORMONE RECEPTOR INTERACTOR 11
9319	TRIP13	TRIP13	THYROID HORMONE RECEPTOR INTERACTOR 13
9325	TRIP4	TRIP4	THYROID HORMONE RECEPTOR INTERACTOR 4
7205	TRIP6	TRIP6	THYROID HORMONE RECEPTOR INTERACTOR 6
7227	TRPS1	TRPS1	TRICHORHINOPHALANGEAL SYNDROME I
8295	TRRAP	TRRAP	TRANSFORMATION/TRANSCRIPTION DOMAIN-ASSOCIATED PROTEIN
8848	TSC22D1	TSC22D1	TSC22 DOMAIN FAMILY, MEMBER 1
9819	TSC22D2	TSC22D2	TSC22 DOMAIN FAMILY, MEMBER 2
1831	TSC22D3	TSC22D3	TSC22 DOMAIN FAMILY, MEMBER 3
81628	TSC22D4	TSC22D4	TSC22 DOMAIN FAMILY, MEMBER 4
7251	TSG101	TSG101	TUMOR SUSCEPTIBILITY GENE 101
10194	TSHZ1	TSHZ1	teashirt zinc finger homeobox 1
128553	TSHZ2	TSHZ2	teashirt zinc finger homeobox 2
57616	TSHZ3	TSHZ3	teashirt zinc finger homeobox 3
8458	TTF2	TTF2	TRANSCRIPTION TERMINATION FACTOR, RNA POLYMERASE II
7289	TULP3	TULP3	TUBBY LIKE PROTEIN 3
56995	TULP4	TULP4	TUBBY LIKE PROTEIN 4
7291	TWIST1	TWIST1	TWIST HOMOLOG 1 (ACROCEPHALOSYNDACTYLY 3; SAETHRE-CHOTZEN SYNDROME) (DROSOPHILA)
117581	TWIST2	TWIST2	TWIST HOMOLOG 2 (DROSOPHILA)
29855	UBN1	UBN1	UBINUCLEIN 1
7342	UBP1	UBP1	UPSTREAM BINDING PROTEIN 1 (LBP-1A)
7343	UBTF	UBTF	UPSTREAM BINDING TRANSCRIPTION FACTOR, RNA POLYMERASE I
5976	UPF1	UPF1	UPF1 REGULATOR OF NONSENSE TRANSCRIPTS HOMOLOG (YEAST)
7391	USF1	USF1	UPSTREAM TRANSCRIPTION FACTOR 1
7392	USF2	USF2	UPSTREAM TRANSCRIPTION FACTOR 2, C-FOS INTERACTING
8433	UTF1	UTF1	UNDIFFERENTIATED EMBRYONIC CELL TRANSCRIPTION FACTOR 1
11023	VAX1	VAX1	VENTRAL ANTERIOR HOMEOBOX 1
25806	VAX2	VAX2	VENTRAL ANTERIOR HOMEOBOX 2
9084	VCY	VCY	VARIABLE CHARGE, Y-LINKED
7421	VDR	VDR	VITAMIN D (1,25- DIHYDROXYVITAMIN D3) RECEPTOR
27287	VENTX	VENTX	VENT HOMEOBOX HOMOLOG (XENOPUS LAEVIS)
51442	VGLL1	VGLL1	VESTIGIAL LIKE 1 (DROSOPHILA)
245806	VGLL2	VGLL2	VESTIGIAL LIKE 2 (DROSOPHILA)
7428	VHL	VHL	VON HIPPEL-LINDAU TUMOR SUPPRESSOR

30813	VSX1	VSX1	VISUAL SYSTEM HOMEOBOX 1 HOMOLOG, CHX10-LIKE (ZEBRAFISH)
51729	WBP11	WBP11	WW DOMAIN BINDING PROTEIN 11
11169	WDHD1	WDHD1	WD REPEAT AND HMG-BOX DNA BINDING PROTEIN 1
7468	WHSC1	WHSC1	WOLF-HIRSCHHORN SYNDROME CANDIDATE 1
54904	WHSC1L1	WHSC1L1	WOLF-HIRSCHHORN SYNDROME CANDIDATE 1-LIKE 1
7469	WHSC2	WHSC2	WOLF-HIRSCHHORN SYNDROME CANDIDATE 2
7490	WT1	WT1	WILMS TUMOR 1
11059	WWP1	WWP1	WW DOMAIN CONTAINING E3 UBIQUITIN PROTEIN LIGASE 1
25937	WWTR1	WWTR1	WW DOMAIN CONTAINING TRANSCRIPTION REGULATOR 1
56949	XAB2	XAB2	XPA BINDING PROTEIN 2
7494	XBP1	XBP1	X-BOX BINDING PROTEIN 1
7508	XPC	XPC	XERODERMA PIGMENTOSUM, COMPLEMENTATION GROUP C
7517	XRCC3	XRCC3	X-RAY REPAIR COMPLEMENTING DEFECTIVE REPAIR IN CHINESE HAMSTER CELLS 3
7520	XRCC5	XRCC5	X-RAY REPAIR COMPLEMENTING DEFECTIVE REPAIR IN CHINESE HAMSTER CELLS 5 (DOUBLE-STRAND-BREAK REJOINING; KU AUTOANTIGEN, 80KDA)
2547	XRCC6	XRCC6	X-RAY REPAIR COMPLEMENTING DEFECTIVE REPAIR IN CHINESE HAMSTER CELLS 6 (KU AUTOANTIGEN, 70KDA)
10138	YAF2	YAF2	YY1 ASSOCIATED FACTOR 2
4904	YBX1	YBX1	Y BOX BINDING PROTEIN 1
51087	YBX2	YBX2	Y BOX BINDING PROTEIN 2
55689	YEATS2	YEATS2	YEATS DOMAIN CONTAINING 2
8089	YEATS4	YEATS4	YEATS DOMAIN CONTAINING 4
91746	YTHDC1	YTHDC1	YTH DOMAIN CONTAINING 1
7528	YY1	YY1	YY1 TRANSCRIPTION FACTOR
22890	ZBTB1	ZBTB1	ZINC FINGER AND BTB DOMAIN CONTAINING 1
65986	ZBTB10	ZBTB10	ZINC FINGER AND BTB DOMAIN CONTAINING 10
27107	ZBTB11	ZBTB11	ZINC FINGER AND BTB DOMAIN CONTAINING 11
221527	ZBTB12	ZBTB12	ZINC FINGER AND BTB DOMAIN CONTAINING 12
7704	ZBTB16	ZBTB16	ZINC FINGER AND BTB DOMAIN CONTAINING 16
7709	ZBTB17	ZBTB17	ZINC FINGER AND BTB DOMAIN CONTAINING 17
57621	ZBTB2	ZBTB2	ZINC FINGER AND BTB DOMAIN CONTAINING 2
26137	ZBTB20	ZBTB20	ZINC FINGER AND BTB DOMAIN CONTAINING 20
9841	ZBTB24	ZBTB24	ZINC FINGER AND BTB DOMAIN CONTAINING 24
7597	ZBTB25	ZBTB25	ZINC FINGER AND BTB DOMAIN CONTAINING 25
57684	ZBTB26	ZBTB26	ZINC FINGER AND BTB DOMAIN CONTAINING 26
79842	ZBTB3	ZBTB3	ZINC FINGER AND BTB DOMAIN CONTAINING 3
27033	ZBTB32	ZBTB32	ZINC FINGER AND BTB DOMAIN CONTAINING 32
10009	ZBTB33	ZBTB33	ZINC FINGER AND BTB DOMAIN CONTAINING 33
403341	ZBTB34	ZBTB34	ZINC FINGER AND BTB DOMAIN CONTAINING 34
84614	ZBTB37	ZBTB37	ZINC FINGER AND BTB DOMAIN CONTAINING 37
253461	ZBTB38	ZBTB38	ZINC FINGER AND BTB DOMAIN CONTAINING 38
9880	ZBTB39	ZBTB39	ZINC FINGER AND BTB DOMAIN CONTAINING 39
9923	ZBTB40	ZBTB40	ZINC FINGER AND BTB DOMAIN CONTAINING 40
9925	ZBTB5	ZBTB5	ZINC FINGER AND BTB DOMAIN CONTAINING 5
51341	ZBTB7A	ZBTB7A	ZINC FINGER AND BTB DOMAIN CONTAINING 7A
51043	ZBTB7B	ZBTB7B	ZINC FINGER AND BTB DOMAIN CONTAINING 7B
127557	ZBTB8	ZBTB8	ZINC FINGER AND BTB DOMAIN CONTAINING 8
221504	ZBTB9	ZBTB9	ZINC FINGER AND BTB DOMAIN CONTAINING 9
23211	C19orf7	ZC3H4	zinc finger CCCH-type containing 4
29800	ZDHHC1	ZDHHC1	ZINC FINGER, DHHC-TYPE CONTAINING 1
54503	ZDHHC13	ZDHHC13	ZINC FINGER, DHHC-TYPE CONTAINING 13
158866	ZDHHC15	ZDHHC15	ZINC FINGER, DHHC-TYPE CONTAINING 15
84287	ZDHHC16	ZDHHC16	ZINC FINGER, DHHC-TYPE CONTAINING 16
51201	ZDHHC2	ZDHHC2	ZINC FINGER, DHHC-TYPE CONTAINING 2
340481	ZDHHC21	ZDHHC21	ZINC FINGER, DHHC-TYPE CONTAINING 21
64429	ZDHHC6	ZDHHC6	ZINC FINGER, DHHC-TYPE CONTAINING 6
55625	ZDHHC7	ZDHHC7	ZINC FINGER, DHHC-TYPE CONTAINING 7
51114	ZDHHC9	ZDHHC9	ZINC FINGER, DHHC-TYPE CONTAINING 9
58487	ZF	ZF	HCF-BINDING TRANSCRIPTION FACTOR ZHANGFEI
9839	ZFHX1B	ZFHX1B	ZINC FINGER HOMEOBOX 1B
85446	ZFHX2	ZFHX2	ZINC FINGER HOMEOBOX 2
162239	ZFP1	ZFP1	ZINC FINGER PROTEIN 1 HOMOLOG (MOUSE)
57677	KIAA1559	ZFP14	MOUSE ZINC FINGER PROTEIN 14-LIKE
7541	ZFP161	ZFP161	ZINC FINGER PROTEIN 161 HOMOLOG (MOUSE)
80108	ZFP2	ZFP2	ZINC FINGER PROTEIN 2 HOMOLOG (MOUSE)
140612	ZFP28	ZFP28	ZINC FINGER PROTEIN 28
22835	ZFP30	ZFP30	ZINC FINGER PROTEIN 30 HOMOLOG (MOUSE)
7538	ZFP36	ZFP36	ZINC FINGER PROTEIN 36, C3H TYPE, HOMOLOG (MOUSE)
677	ZFP36L1	ZFP36L1	ZINC FINGER PROTEIN 36, C3H TYPE-LIKE 1
678	ZFP36L2	ZFP36L2	ZINC FINGER PROTEIN 36, C3H TYPE-LIKE 2
7539	ZFP37	ZFP37	ZINC FINGER PROTEIN 37 HOMOLOG (MOUSE)
286128	ZFP41	ZFP41	ZINC FINGER PROTEIN 41
132625	ZFP42	ZFP42	ZINC FINGER PROTEIN 42
346171	ZFP57	ZFP57	ZINC FINGER PROTEIN 57 HOMOLOG (MOUSE)
92379	ZFP62	ZFP62	ZINC FINGER PROTEIN 62 HOMOLOG (MOUSE)
55734	ZFP64	ZFP64	ZINC FINGER PROTEIN 64 HOMOLOG (MOUSE)
146198	ZFP90	ZFP90	ZINC FINGER PROTEIN 90 HOMOLOG (MOUSE)
80829	ZFP91	ZFP91	ZINC FINGER PROTEIN 91 HOMOLOG (MOUSE)
23660	ZFP95	ZFP95	ZINC FINGER PROTEIN 95 HOMOLOG (MOUSE)
7542	ZFPL1	ZFPL1	ZINC FINGER PROTEIN-LIKE 1
161882	ZFPM1	ZFPM1	ZINC FINGER PROTEIN, MULTITYPE 1
23414	ZFPM2	ZFPM2	ZINC FINGER PROTEIN, MULTITYPE 2
7543	ZFX	ZFX	ZINC FINGER PROTEIN, X-LINKED
7544	ZFY	ZFY	ZINC FINGER PROTEIN, Y-LINKED
11244	ZHX1	ZHX1	ZINC FINGERS AND HOMEOBOXES 1
22882	ZHX2	ZHX2	ZINC FINGERS AND HOMEOBOXES 2
23051	ZHX3	ZHX3	ZINC FINGERS AND HOMEOBOXES 3
7545	ZIC1	ZIC1	ZIC FAMILY MEMBER 1 (ODD-PAIRED HOMOLOG, DROSOPHILA)
7546	ZIC2	ZIC2	ZIC FAMILY MEMBER 2 (ODD-PAIRED HOMOLOG, DROSOPHILA)
7547	ZIC3	ZIC3	ZIC FAMILY MEMBER 3 HETEROTAXY 1 (ODD-PAIRED HOMOLOG, DROSOPHILA)
23619	ZIM2	ZIM2	ZINC FINGER, IMPRINTED 2
114026	ZIM3	ZIM3	ZINC FINGER, IMPRINTED 3
7586	ZKSCAN1	ZKSCAN1	ZINC FINGER WITH KRAB AND SCAN DOMAINS 1
10771	ZMYND11	ZMYND11	ZINC FINGER, MYND DOMAIN CONTAINING 11
7556	ZNF10	ZNF10	ZINC FINGER PROTEIN 10
163227	ZNF100	ZNF100	ZINC FINGER PROTEIN 100
94039	ZNF101	ZNF101	ZINC FINGER PROTEIN 101
163071	ZNF114	ZNF114	ZINC FINGER PROTEIN 114
7670	ZNF117	ZNF117	ZINC FINGER PROTEIN 117 (HPF9)
7558	ZNF11B	ZNF11B	ZINC FINGER PROTEIN 11B
7559	ZNF12	ZNF12	ZINC FINGER PROTEIN 12
7675	ZNF121	ZNF121	ZINC FINGER PROTEIN 121 (CLONE ZHC32)
7678	ZNF124	ZNF124	ZINC FINGER PROTEIN 124 (HZF-16)
7690	ZNF131	ZNF131	ZINC FINGER PROTEIN 131 (CLONE PHZ-10)
7691	ZNF132	ZNF132	ZINC FINGER PROTEIN 132 (CLONE PHZ-12)
7692	ZNF133	ZNF133	ZINC FINGER PROTEIN 133 (CLONE PHZ-13)
7693	ZNF134	ZNF134	ZINC FINGER PROTEIN 134 (CLONE PHZ-15)
7694	ZNF135	ZNF135	ZINC FINGER PROTEIN 135 (CLONE PHZ-17)
7695	ZNF136	ZNF136	ZINC FINGER PROTEIN 136 (CLONE PHZ-20)
7696	ZNF137	ZNF137	ZINC FINGER PROTEIN 137 (CLONE PHZ-30)
7697	ZNF138	ZNF138	ZINC FINGER PROTEIN 138
7561	ZNF14	ZNF14	ZINC FINGER PROTEIN 14 (KOX 6)
7699	ZNF140	ZNF140	ZINC FINGER PROTEIN 140 (CLONE PHZ-39)
7700	ZNF141	ZNF141	ZINC FINGER PROTEIN 141 (CLONE PHZ-44)
7701	ZNF142	ZNF142	ZINC FINGER PROTEIN 142 (CLONE PHZ-49)
7702	ZNF143	ZNF143	ZINC FINGER PROTEIN 143 (CLONE PHZ-1)
7705	ZNF146	ZNF146	ZINC FINGER PROTEIN 146
7707	ZNF148	ZNF148	ZINC FINGER PROTEIN 148 (PHZ-52)
7710	ZNF154	ZNF154	ZINC FINGER PROTEIN 154 (PHZ-92)
7711	ZNF155	ZNF155	ZINC FINGER PROTEIN 155 (PHZ-96)
7712	ZNF157	ZNF157	ZINC FINGER PROTEIN 157 (HZF22)
7564	ZNF16	ZNF16	ZINC FINGER PROTEIN 16 (KOX 9)
90338	ZNF160	ZNF160	ZINC FINGER PROTEIN 160
7716	ZNF161	ZNF161	ZINC FINGER PROTEIN 161
7718	ZNF165	ZNF165	ZINC FINGER PROTEIN 165
55888	ZNF167	ZNF167	ZINC FINGER PROTEIN 167
169841	ZNF169	ZNF169	ZINC FINGER PROTEIN 169
7565	ZNF17	ZNF17	ZINC FINGER PROTEIN 17 (HPF3, KOX 10)
7727	ZNF174	ZNF174	ZINC FINGER PROTEIN 174
7728	ZNF175	ZNF175	ZINC FINGER PROTEIN 175
7730	ZNF177	ZNF177	ZINC FINGER PROTEIN 177

7566	ZNF18	ZNF18	ZINC FINGER PROTEIN 18 (KOX 11)
7733	ZNF180	ZNF180	ZINC FINGER PROTEIN 180 (HHZ168)
7738	ZNF184	ZNF184	ZINC FINGER PROTEIN 184 (KRUPPEL-LIKE)
7741	ZNF187	ZNF187	ZINC FINGER PROTEIN 187
7743	ZNF189	ZNF189	ZINC FINGER PROTEIN 189
7567	ZNF19	ZNF19	ZINC FINGER PROTEIN 19 (KOX 12)
7571	ZNF23	ZNF19	ZINC FINGER PROTEIN 19 (KOX 12)
7745	ZNF192	ZNF192	ZINC FINGER PROTEIN 192
7746	ZNF193	ZNF193	ZINC FINGER PROTEIN 193
7748	ZNF195	ZNF195	ZINC FINGER PROTEIN 195
10168	ZNF197	ZNF197	ZINC FINGER PROTEIN 197
7750	ZNF198	ZNF198	ZINC FINGER PROTEIN 198
7549	ZNF2	ZNF2	ZINC FINGER PROTEIN 2
7568	ZNF20	ZNF20	ZINC FINGER PROTEIN 20 (KOX 13)
7752	ZNF200	ZNF200	ZINC FINGER PROTEIN 200
7753	ZNF202	ZNF202	ZINC FINGER PROTEIN 202
7755	ZNF205	ZNF205	ZINC FINGER PROTEIN 205
84891	ZNF206	ZNF206	ZINC FINGER PROTEIN 206
7756	ZNF207	ZNF207	ZINC FINGER PROTEIN 207
7757	ZNF208	ZNF208	ZINC FINGER PROTEIN 208
7569	ZNF21	ZNF21	ZINC FINGER PROTEIN 21 (KOX 14)
10520	ZNF211	ZNF211	ZINC FINGER PROTEIN 211
7988	ZNF212	ZNF212	ZINC FINGER PROTEIN 212
7760	ZNF213	ZNF213	ZINC FINGER PROTEIN 213
7761	ZNF214	ZNF214	ZINC FINGER PROTEIN 214
7762	ZNF215	ZNF215	ZINC FINGER PROTEIN 215
7764	ZNF217	ZNF217	ZINC FINGER PROTEIN 217
51222	ZNF219	ZNF219	ZINC FINGER PROTEIN 219
7570	ZNF22	ZNF22	ZINC FINGER PROTEIN 22 (KOX 15)
7638	ZNF221	ZNF221	ZINC FINGER PROTEIN 221
7673	ZNF222	ZNF222	ZINC FINGER PROTEIN 222
7766	ZNF223	ZNF223	ZINC FINGER PROTEIN 223
7767	ZNF224	ZNF224	ZINC FINGER PROTEIN 224
7768	ZNF225	ZNF225	ZINC FINGER PROTEIN 225
7769	ZNF226	ZNF226	ZINC FINGER PROTEIN 226
7770	ZNF227	ZNF227	ZINC FINGER PROTEIN 227
7771	ZNF228	ZNF228	ZINC FINGER PROTEIN 228
7772	ZNF229	ZNF229	ZINC FINGER PROTEIN 229
7773	ZNF230	ZNF230	ZINC FINGER PROTEIN 230
7775	ZNF232	ZNF232	ZINC FINGER PROTEIN 232
10780	ZNF234	ZNF234	ZINC FINGER PROTEIN 234
9310	ZNF235	ZNF235	ZINC FINGER PROTEIN 235
7776	ZNF236	ZNF236	ZINC FINGER PROTEIN 236
10472	ZNF238	ZNF238	ZINC FINGER PROTEIN 238
8187	ZNF239	ZNF239	ZINC FINGER PROTEIN 239
7572	ZNF24	ZNF24	ZINC FINGER PROTEIN 24 (KOX 17)
57209	ZNF248	ZNF248	ZINC FINGER PROTEIN 248
219749	ZNF25	ZNF25	ZINC FINGER PROTEIN 25 (KOX 19)
58500	ZNF250	ZNF250	ZINC FINGER PROTEIN 250
90987	ZNF251	ZNF251	ZINC FINGER PROTEIN 251
56242	ZNF253	ZNF253	ZINC FINGER PROTEIN 253
9534	ZNF254	ZNF254	ZINC FINGER PROTEIN 254
10172	ZNF256	ZNF256	ZINC FINGER PROTEIN 256
113835	ZNF257	ZNF257	ZINC FINGER PROTEIN 257
7574	ZNF26	ZNF26	ZINC FINGER PROTEIN 26 (KOX 20)
10127	ZNF263	ZNF263	ZINC FINGER PROTEIN 263
9422	ZNF264	ZNF264	ZINC FINGER PROTEIN 264
9406	ZNF265	ZNF265	ZINC FINGER PROTEIN 265
10781	ZNF266	ZNF266	ZINC FINGER PROTEIN 266
10308	ZNF267	ZNF267	ZINC FINGER PROTEIN 267
10795	ZNF268	ZNF268	ZINC FINGER PROTEIN 268
7575	ZNF27	ZNF27	ZINC FINGER PROTEIN 27 (KOX 22)
10778	ZNF271	ZNF271	ZINC FINGER PROTEIN 271
10794	ZNF272	ZNF272	ZINC FINGER PROTEIN 272
10793	ZNF273	ZNF273	ZINC FINGER PROTEIN 273
10782	ZNF274	ZNF274	ZINC FINGER PROTEIN 274
10838	ZNF275	ZNF275	ZINC FINGER PROTEIN 275
92822	ZNF276	ZNF276	ZINC FINGER PROTEIN 276
11179	ZNF277	ZNF277	ZINC FINGER PROTEIN 277
23598	ZNF278	ZNF278	ZINC FINGER PROTEIN 278
23528	ZNF281	ZNF281	ZINC FINGER PROTEIN 281
8427	ZNF282	ZNF282	ZINC FINGER PROTEIN 282
284349	ZNF283	ZNF283	ZINC FINGER PROTEIN 283
26974	ZNF285	ZNF285	ZINC FINGER PROTEIN 285
57335	ZNF286	ZNF286	ZINC FINGER PROTEIN 286
57336	ZNF287	ZNF287	ZINC FINGER PROTEIN 287
7577	ZNF29	ZNF29	ZINC FINGER PROTEIN 29 (KOX 26)
23036	ZNF292	ZNF292	ZINC FINGER PROTEIN 292
49854	ZNF295	ZNF295	ZINC FINGER PROTEIN 295
9278	ZBTB22	ZNF297	ZINC FINGER PROTEIN 297
23099	ZBTB43	ZNF297B	ZINC FINGER PROTEIN 297B
7551	ZNF3	ZNF3	ZINC FINGER PROTEIN 3 (A8-51)
90075	ZNF30	ZNF30	ZINC FINGER PROTEIN 30 (KOX 28)
91975	ZNF300	ZNF300	ZINC FINGER PROTEIN 300
55900	ZNF302	ZNF302	ZINC FINGER PROTEIN ZNF140-LIKE PROTEIN
57343	ZNF304	ZNF304	ZINC FINGER PROTEIN 304
80317	ZNF306	ZNF306	ZINC FINGER PROTEIN 306
387032	ZNF307	ZNF307	ZINC FINGER PROTEIN 307
282890	ZNF311	ZNF311	ZINC FINGER PROTEIN 311
57693	ZNF317	ZNF317	ZINC FINGER PROTEIN 317
57567	ZNF319	ZNF319	ZINC FINGER PROTEIN 319
7580	ZNF32	ZNF32	ZINC FINGER PROTEIN 32 (KOX 30)
64288	ZNF323	ZNF323	ZINC FINGER PROTEIN 323
25799	ZNF324	ZNF324	ZINC FINGER PROTEIN 324
284695	ZNF326	ZNF326	ZINC FINGER PROTEIN 326
55422	ZNF331	ZNF331	ZINC FINGER PROTEIN 331
84449	ZNF333	ZNF333	ZINC FINGER PROTEIN 333
55713	ZNF334	ZNF334	ZINC FINGER PROTEIN 334
63925	ZNF335	ZNF335	ZINC FINGER PROTEIN 335
64412	GZF1	ZNF336	ZINC FINGER PROTEIN 336
26152	ZNF337	ZNF337	ZINC FINGER PROTEIN 337
7581	ZNF33A	ZNF33A	ZINC FINGER PROTEIN 33A
80778	ZNF34	ZNF34	ZINC FINGER PROTEIN 34 (KOX 32)
84905	ZNF341	ZNF341	ZINC FINGER PROTEIN 341
162979	ZNF342	ZNF342	ZINC FINGER PROTEIN 342
79175	ZNF343	ZNF343	ZINC FINGER PROTEIN 343
25850	ZNF345	ZNF345	ZINC FINGER PROTEIN 345
84671	ZNF347	ZNF347	ZINC FINGER PROTEIN 347
7584	ZNF35	ZNF35	ZINC FINGER PROTEIN 35 (CLONE HF.10)
59348	ZNF350	ZNF350	ZINC FINGER PROTEIN 350
6940	ZNF354A	ZNF354A	ZINC FINGER PROTEIN 354A
117608	ZNF354B	ZNF354B	ZINC FINGER PROTEIN 354B
30832	ZNF354C	ZNF354C	ZINC FINGER PROTEIN 354C
140467	ZNF358	ZNF358	ZINC FINGER PROTEIN 358
167465	ZNF366	ZNF366	ZINC FINGER PROTEIN 366
7587	ZNF37A	ZNF37A	ZINC FINGER PROTEIN 37A (KOX 21)
7589	ZNF38	ZNF38	ZINC FINGER PROTEIN 38
84911	ZNF382	ZNF382	ZINC FINGER PROTEIN 382
163087	ZNF383	ZNF383	ZINC FINGER PROTEIN 383
171017	ZNF384	ZNF384	ZINC FINGER PROTEIN 384
25946	ZNF385	ZNF385	ZINC FINGER PROTEIN 385
222696	ZNF390	ZNF390	ZINC FINGER PROTEIN 390
84124	ZNF394	ZNF394	ZINC FINGER PROTEIN 394
55893	ZNF395	ZNF395	ZINC FINGER PROTEIN 395
252884	ZNF396	ZNF396	ZINC FINGER PROTEIN 396
84307	ZNF397	ZNF397	ZINC FINGER PROTEIN 397
57541	ZNF398	ZNF398	ZINC FINGER PROTEIN 398
342908	ZNF404	ZNF404	ZINC FINGER PROTEIN 404
55628	ZNF407	ZNF407	ZINC FINGER PROTEIN 407

79797	ZNF408	ZNF408	ZINC FINGER PROTEIN 408
22830	ZNF409	ZNF409	ZINC FINGER PROTEIN 409
7592	ZNF41	ZNF41	ZINC FINGER PROTEIN 41
57862	ZNF410	ZNF410	ZINC FINGER PROTEIN 410
55659	ZNF416	ZNF416	ZINC FINGER PROTEIN 416
147687	ZNF417	ZNF417	ZINC FINGER PROTEIN 417
147686	ZNF418	ZNF418	ZINC FINGER PROTEIN 418
79744	ZNF419	ZNF419	ZINC FINGER PROTEIN 419
7593	ZNF42	ZNF42	ZINC FINGER PROTEIN 42 (MYELOID-SPECIFIC RETINOIC ACID-RESPONSIVE)
147923	ZNF420	ZNF420	ZINC FINGER PROTEIN 420
23090	ZNF423	ZNF423	ZINC FINGER PROTEIN 423
79088	ZNF426	ZNF426	ZINC FINGER PROTEIN 426
353088	ZNF429	ZNF429	ZINC FINGER PROTEIN 429
7594	ZNF43	ZNF43	ZINC FINGER PROTEIN 43 (HTF6)
80264	ZNF430	ZNF430	ZINC FINGER PROTEIN 430
170959	ZNF431	ZNF431	ZINC FINGER PROTEIN 431
9668	ZNF432	ZNF432	ZINC FINGER PROTEIN 432
163059	ZNF433	ZNF433	ZINC FINGER PROTEIN 433
54925	ZNF434	ZNF434	ZINC FINGER PROTEIN 434
80345	ZNF435	ZNF435	ZINC FINGER PROTEIN 435
80818	ZNF436	ZNF436	ZINC FINGER PROTEIN 436
90594	ZNF439	ZNF439	ZINC FINGER PROTEIN 439
51710	ZNF44	ZNF44	ZINC FINGER PROTEIN 44 (KOX 7)
126070	ZNF440	ZNF440	ZINC FINGER PROTEIN 440
126068	ZNF441	ZNF441	ZINC FINGER PROTEIN 441
79973	ZNF442	ZNF442	ZINC FINGER PROTEIN 442
10224	ZNF443	ZNF443	ZINC FINGER PROTEIN 443
55311	ZNF444	ZNF444	ZINC FINGER PROTEIN 444
353274	ZNF445	ZNF445	ZINC FINGER PROTEIN 445
55663	ZNF446	ZNF446	ZINC FINGER PROTEIN 446
65982	ZNF447	ZNF447	ZINC FINGER PROTEIN 447
203523	ZNF449	ZNF449	ZINC FINGER PROTEIN 449
7596	ZNF45	ZNF45	ZINC FINGER PROTEIN 45
26036	ZNF451	ZNF451	ZINC FINGER PROTEIN 451
285676	ZNF454	ZNF454	ZINC FINGER PROTEIN 454
58499	ZNF462	ZNF462	ZINC FINGER PROTEIN 462
168544	ZNF467	ZNF467	ZINC FINGER PROTEIN 467
84627	ZNF469	ZNF469	ZINC FINGER PROTEIN 469
57573	ZNF471	ZNF471	ZINC FINGER PROTEIN 471
25888	ZNF473	ZNF473	ZINC FINGER PROTEIN 473
90827	ZNF479	ZNF479	ZINC FINGER PROTEIN 479
147657	ZNF480	ZNF480	ZINC FINGER PROTEIN 480
10773	ZBTB6	ZNF482	ZINC FINGER PROTEIN 482
158399	ZNF483	ZNF483	ZINC FINGER PROTEIN 483
90649	ZNF486	ZNF486	ZINC FINGER PROTEIN 486
118738	ZNF488	ZNF488	ZINC FINGER PROTEIN 488
57474	ZNF490	ZNF490	ZINC FINGER PROTEIN 490
126069	ZNF491	ZNF491	ZINC FINGER PROTEIN 491
284443	LOC115648	ZNF493	zinc finger protein 493
284443	ZNF493	ZNF493	ZINC FINGER PROTEIN 493
84838	ZNF496	ZNF496	ZINC FINGER PROTEIN 496
84878	ZNF499	ZNF499	ZINC FINGER PROTEIN 499
26048	ZNF500	ZNF500	ZINC FINGER PROTEIN 500
115560	ZNF501	ZNF501	ZINC FINGER PROTEIN 501
91392	ZNF502	ZNF502	ZINC FINGER PROTEIN 502
22847	ZNF507	ZNF507	ZINC FINGER PROTEIN 507
22869	ZNF510	ZNF510	ZINC FINGER PROTEIN 510
84450	ZNF512	ZNF512	ZINC FINGER PROTEIN 512
57473	GM632	ZNF512B	zinc finger protein 512B
130557	ZNF513	ZNF513	ZINC FINGER PROTEIN 513
84874	ZNF514	ZNF514	ZINC FINGER PROTEIN 514
162655	ZNF519	ZNF519	ZINC FINGER PROTEIN 519
147807	ZNF524	ZNF524	ZINC FINGER PROTEIN 524
163255	ZNF540	ZNF540	ZINC FINGER PROTEIN 540
27300	ZNF544	ZNF544	ZINC FINGER PROTEIN 544
284406	ZNF545	ZNF545	ZINC FINGER PROTEIN 545
339327	ZNF546	ZNF546	ZINC FINGER PROTEIN 546
284306	ZNF547	ZNF547	ZINC FINGER PROTEIN 547
256051	ZNF549	ZNF549	ZINC FINGER PROTEIN 549
162972	ZNF550	ZNF550	ZINC FINGER PROTEIN 550
90233	ZNF551	ZNF551	ZINC FINGER PROTEIN 551
197407	ZNF553	ZNF553	ZINC FINGER PROTEIN 553
115196	ZNF554	ZNF554	ZINC FINGER PROTEIN 554
148254	ZNF555	ZNF555	ZINC FINGER PROTEIN 555
80032	ZNF556	ZNF556	ZINC FINGER PROTEIN 556
79230	ZNF557	ZNF557	ZINC FINGER PROTEIN 557
148156	ZNF558	ZNF558	ZINC FINGER PROTEIN 558
84527	ZNF559	ZNF559	ZINC FINGER PROTEIN 559
147741	ZNF560	ZNF560	ZINC FINGER PROTEIN 560
147837	ZNF563	ZNF563	ZINC FINGER PROTEIN 563
163050	ZNF564	ZNF564	ZINC FINGER PROTEIN 564
147929	ZNF565	ZNF565	ZINC FINGER PROTEIN 565
84924	ZNF566	ZNF566	ZINC FINGER PROTEIN 566
163081	ZNF567	ZNF567	ZINC FINGER PROTEIN 567
374900	ZNF568	ZNF568	ZINC FINGER PROTEIN 568
126295	LOC126295	ZNF57	zinc finger protein 47
148268	ZNF570	ZNF570	ZINC FINGER PROTEIN 570
51276	ZNF571	ZNF571	ZINC FINGER PROTEIN 571
126231	ZNF573	ZNF573	ZINC FINGER PROTEIN 573
84765	ZNF577	ZNF577	ZINC FINGER PROTEIN 577
147660	ZNF578	ZNF578	ZINC FINGER PROTEIN 578
51157	ZNF580	ZNF580	ZINC FINGER PROTEIN 580
51545	ZNF581	ZNF581	ZINC FINGER PROTEIN 581
147948	ZNF582	ZNF582	ZINC FINGER PROTEIN 582
147949	ZNF583	ZNF583	ZINC FINGER PROTEIN 583
201514	ZNF584	ZNF584	ZINC FINGER PROTEIN 584
199704	ZNF585A	ZNF585A	ZINC FINGER PROTEIN 585A
92285	ZNF585B	ZNF585B	ZINC FINGER PROTEIN 585B
54807	ZNF586	ZNF586	ZINC FINGER PROTEIN 586
84914	ZNF587	ZNF587	ZINC FINGER PROTEIN 587
51427	ZNF588	ZNF588	ZINC FINGER PROTEIN 588
51385	ZNF589	ZNF589	ZINC FINGER PROTEIN 589
9640	ZNF592	ZNF592	ZINC FINGER PROTEIN 592
51042	ZNF593	ZNF593	ZINC FINGER PROTEIN 593
152687	ZNF595	ZNF595	ZINC FINGER PROTEIN 595
169270	ZNF596	ZNF596	ZINC FINGER PROTEIN 596
146434	ZNF597	ZNF597	ZINC FINGER PROTEIN 597
148103	ZNF599	ZNF599	ZINC FINGER PROTEIN 599
7552	ZNF6	ZNF6	ZINC FINGER PROTEIN 6 (CMPX1)
80095	ZNF606	ZNF606	ZINC FINGER PROTEIN 606
162963	ZNF610	ZNF610	ZINC FINGER PROTEIN 610
81856	ZNF611	ZNF611	ZINC FINGER PROTEIN 611
79898	ZNF613	ZNF613	ZINC FINGER PROTEIN 613
284370	ZNF615	ZNF615	ZINC FINGER PROTEIN 615
9831	ZNF623	ZNF623	ZINC FINGER PROTEIN 623
57547	ZNF624	ZNF624	ZINC FINGER PROTEIN 624
199777	ZNF626	ZNF626	ZINC FINGER PROTEIN 626
199692	ZNF627	ZNF627	ZINC FINGER PROTEIN 627
27332	ZNF638	ZNF638	ZINC FINGER PROTEIN 638
51193	ZNF639	ZNF639	ZINC FINGER PROTEIN 639
121274	ZNF641	ZNF641	ZINC FINGER PROTEIN 641
84146	ZNF644	ZNF644	ZINC FINGER PROTEIN 644
9726	ZNF646	ZNF646	ZINC FINGER PROTEIN 646
65251	ZNF649	ZNF649	ZINC FINGER PROTEIN 649
115950	ZNF653	ZNF653	ZINC FINGER PROTEIN 653
79027	ZNF655	ZNF655	ZINC FINGER PROTEIN 655
79862	ZNF669	ZNF669	ZINC FINGER PROTEIN 669
93474	ZNF670	ZNF670	ZINC FINGER PROTEIN 670
79891	ZNF671	ZNF671	ZINC FINGER PROTEIN 671

55634	ZNF673	ZNF673	ZINC FINGER PROTEIN 673
171392	ZNF675	ZNF675	ZINC FINGER PROTEIN 675
342926	ZNF677	ZNF677	ZINC FINGER PROTEIN 677
168417	ZNF679	ZNF679	ZINC FINGER PROTEIN 679
146542	ZNF688	ZNF688	ZINC FINGER PROTEIN 688
115509	ZNF689	ZNF689	ZINC FINGER PROTEIN 689
57116	ZNF695	ZNF695	ZINC FINGER PROTEIN 695
7553	ZNF7	ZNF7	ZINC FINGER PROTEIN 7 (KOX 4, CLONE HF.16)
7621	ZNF70	ZNF70	ZINC FINGER PROTEIN 70 (COS17)
90592	ZNF700	ZNF700	ZINC FINGER PROTEIN 700
286075	ZNF707	ZNF707	ZINC FINGER PROTEIN 707
7562	ZNF708	ZNF708	ZINC FINGER PROTEIN 708 (KOX8)
163051	ZNF709	ZNF709	ZINC FINGER PROTEIN 709
58491	ZNF71	ZNF71	ZINC FINGER PROTEIN 71 (COS26)
349075	ZNF713	ZNF713	ZINC FINGER PROTEIN 713
148206	ZNF714	ZNF714	ZINC FINGER PROTEIN 714
148203	LOC148203	ZNF738	zinc finger protein 738
7625	ZNF74	ZNF74	ZINC FINGER PROTEIN 74 (COS52)
7626	ZNF75	ZNF75	ZINC FINGER PROTEIN 75 (D8C6)
7627	ZNF75A	ZNF75A	ZINC FINGER PROTEIN 75A
7629	ZNF76	ZNF76	ZINC FINGER PROTEIN 76 (EXPRESSED IN TESTIS)
92595	MGC13138	ZNF764	zinc finger protein 764
91661	LOC91661	ZNF765	zinc finger protein 765
90321	LOC90321	ZNF766	zinc finger protein 766
79724	FLJ23436	ZNF768	zinc finger protein 768
58492	ZNF77	ZNF77	ZINC FINGER PROTEIN 77 (PT1)
197320	FLJ31875	ZNF778	zinc finger protein 778
146540	FLJ32130	ZNF785	zinc finger protein 785
136051	DKFZp762I137	ZNF786	zinc finger protein 786
7633	ZNF79	ZNF79	ZINC FINGER PROTEIN 79 (PT7)
7554	ZNF8	ZNF8	ZINC FINGER PROTEIN 8 (CLONE HF.18)
7634	ZNF80	ZNF80	ZINC FINGER PROTEIN 80 (PT17)
347344	ZNF81	ZNF81	ZINC FINGER PROTEIN 81 (HFZ20)
55769	ZNF83	ZNF83	ZINC FINGER PROTEIN 83 (HPF1)
7637	ZNF84	ZNF84	ZINC FINGER PROTEIN 84 (HPF2)
7639	ZNF85	ZNF85	ZINC FINGER PROTEIN 85 (HPF4, HTF1)
7555	ZNF9	ZNF9	ZINC FINGER PROTEIN 9 (A CELLULAR RETROVIRAL NUCLEIC ACID BINDING PROTEIN)
7643	ZNF90	ZNF90	ZINC FINGER PROTEIN 90
7644	ZNF91	ZNF91	ZINC FINGER PROTEIN 91 (HPF7, HTF10)
168374	ZNF92	ZNF92	ZINC FINGER PROTEIN 92 (HTF12)
81931	ZNF93	ZNF93	ZINC FINGER PROTEIN 93 (HTF34)
9753	ZNF96	ZNF96	ZINC FINGER PROTEIN 96
10320	ZNFN1A1	ZNFN1A1	ZINC FINGER PROTEIN, SUBFAMILY 1A, 1 (IKAROS)
22807	ZNFN1A2	ZNFN1A2	ZINC FINGER PROTEIN, SUBFAMILY 1A, 2 (HELIOS)
22806	ZNFN1A3	ZNFN1A3	ZINC FINGER PROTEIN, SUBFAMILY 1A, 3 (AIOLOS)
57169	ZNFX1	ZNFX1	ZINC FINGER, NFX1-TYPE CONTAINING 1
9326	ZNHIT3	ZNHIT3	ZINC FINGER, HIT TYPE 3
30834	ZNRD1	ZNRD1	ZINC RIBBON DOMAIN CONTAINING, 1
27000	ZRF1	ZRF1	ZUOTIN RELATED FACTOR 1
284312	ZSCAN1	ZSCAN1	ZINC FINGER AND SCAN DOMAIN CONTAINING 1
54993	ZSCAN2	ZSCAN2	ZINC FINGER AND SCAN DOMAIN CONTAINING 2
201516	ZSCAN4	ZSCAN4	ZINC FINGER AND SCAN DOMAIN CONTAINING 4
79149	ZSCAN5	ZSCAN5	ZINC FINGER AND SCAN DOMAIN CONTAINING 5
65249	ZSWIM4	ZSWIM4	ZINC FINGER, SWIM-TYPE CONTAINING 4
26009	ZZZ3	ZZZ3	ZINC FINGER, ZZ-TYPE CONTAINING 3
