# Supplementary material for: Reconstruction of Monocyte Transcriptional Regulatory Network Accompanies Monocytic Functions in Human Fibroblasts
Source: PLoS One. 2012 Mar 13;7(3):e33474. doi: 10.1371/journal.pone.0033474 (PMC3302774; doi:10.1371/journal.pone.0033474)
Supplement: Table S3 — Primer sequences for qRT-PCR. (PDF) [file pone.0033474.s005.pdf]

Table S3 Primer sequences for qRT-PCR	
Primer ID	Sequence
Matrix over expression analysis	
FOS_UTR_F	TCTGTGCGTGAAACACACCA
FOS_UTR_R	ACTCCATGCGTTTTGCTACATC
FOSB_UTR_F	CTTTTCTCCTCCGCCTGTGT
FOSB_UTR_R	CTCATGTCCCCAACGAACC
IRF8 UTR_F	GGCATTCTCGGAGGAGTAGA
IRF8 UTR_R	GTTACAGCATCCAGGCCATC
SPI1 UTR_F	CCGCTGGCCATAGCATTA
SPI1 UTR_R	CGCCACAGTCCTGCCTCT
MAFB_UTR_F	TCCTGGCTTTCTGAACTTTGC
MAFB_UTR_R	TCTCCTTTCCTCGTTGCTCTCT
JUNB_UTR2_F	GAAACGACGCCAGGAAAGC
JUNB_UTR2_R	TGTGCGCAAAAGCCCTGT
EGR2_UTR_F	GGCTCAGAAGGAGGTGGTGT
EGR2_UTR_R	CAAAATAAGGGGAAGTGGGGTAG
MAF_UTR_F	TCATGCTTCATTGTTTCTCTGG
MAF_UTR_R	CTCTGCCCGTGGATTTGTT
CEBPA UTR_F	CAGAGGGACCGGAGTTATGA
CEBPA UTRR	TTCACATTGCACAAGGCACT
HCLS1_UTR__F	CAGCAGCTCAGTTTCTCACTCC
HCLS1_UTR__R	CACCTGTGCAAGCCTCTGTT
BTG2_UTR_F	GAGGGTCTGGAGGAAAACTGG
BTG2_UTR_R	CCCAAGGAGAGCAGGAGAGA
MXD1_UTR_F	CATCCCTAGAATTGGTGCTCTTG
MXD1_UTR_R	TCATCTGCTGCTCCCCTGT
STAT5A_UTR_F	CCTTCTTTGCTTGCCTCTCTGT
STAT5A_UTR_R	CCTGTCCACCCACCATATCC
ZFP36_UTR_F	CAAACCCCACCCATAAATCAA
ZFP36_UTR_R	AAAATACAAGGGAAGCAGACGA
CREG1_UTR_F	TTCCTCAACTGCCCCAAA
CREG1_UTR_R	AATGCCATGTTCTCTTCCCTCT
LYL1_UTR_F	GGACCAGTGAAGACGTCAGG
LYL1_UTR_R	CAGAGGGTTGTGGGTGATTTT
LMO2_UTR_F	TGACGCTTGCGAACTAGAGA
LMO2_UTR_R	AAGCTTAAGGCCTTGGGAAG
MNDA_UTR_F	TGGCTCTAACAAGTGCCATT
MNDA_UTR_R	AATGGATGCTTCTTCCACAA
NR4A2_UTR_F	CTTCGGCAGAGTTGAATGAATG
NR4A2_UTR_R	AAAAGCAATGGGGAGTCCAG
LPS response	
TNF_F	GCCTGCTGCACTTTGGAGT
TNF_R	CTCGGGGTTCGAGAAGATG
IL6_F	CACCTCTTCAGAACGAATTGACA
IL6_R	CCTCTTTGCTGCTTTCACACA
IL1A_F	TGAAGAAGACAGTTCCTCCATTGAT
IL1A_R	CATGGAGTGGGCCATAGCTT
IL1B_F	TTTGAAGCTGATGGCCCTAAA
IL1B_R	GTGGTGGTCGGAGATTCGTAG
IL8_F	CACACTGCGCCAACACAGA
IL8_R	CAACCCTCTGCACCCAGTTT
CCL2_F	CAGCAGCAAGTGTCCCAAA
CCL2_R	ATGGAATCCTGAACCCACTTCT
CXCL10_F	TTCCTGCAAGCCAATTTTGT
CXCL10_R	TGATGGCCTTCGATTCTGG
IFNB1_F	ATCTAGCACTGGCTGGAATGAG
IFNB1_R	CCAGGACTGTCTTCAGATGGTTT
